# Electroacupuncture modulates gut-lung microbiota and lung EMT to attenuate airway remodeling in COPD

**DOI:** 10.3389/fmicb.2026.1747151

**Published:** 2026-04-01

**Authors:** Daohong Chen, Zijing Zhou, Ziyang Zhou, Ziwen Wang, Ling Zhao, Ying Chen

**Affiliations:** School of Acupuncture and Tuina, Chengdu University of Traditional Chinese Medicine, Chengdu, Sichuan, China

**Keywords:** COPD, EA, EMT, gut microbiota, lung microbiota

## Abstract

**Background:**

Chronic obstructive pulmonary disease (COPD) airway remodeling is primarily driven by epithelial-mesenchymal transition (EMT), which is exacerbated by gut-lung axis (the bidirectional communication between gut and lung microbiota) dysbiosis and systemic inflammation. Although electroacupuncture (EA) demonstrates therapeutic potential in COPD, its mechanisms in modulating the gut-lung axis to alleviate inflammation and EMT remain unclear.

**Methods:**

In cigarette smoke and lipopolysaccharide (LPS)-induced COPD rats, we evaluated lung function, airway collagen deposition, pro-inflammatory and anti-inflammatory cytokines in serum, bronchoalveolar lavage fluid (BALF), and colon tissue, EMT markers in lung tissue, serum LPS levels, and 16S rRNA sequencing of lung and gut microbiota. Interventions comprised authentic EA at bilateral “Feishu” (BL13) and “Zusanli” (ST36) acupoints versus sham acupuncture at non-acupoint.

**Results:**

Electroacupuncture significantly attenuated airway remodeling, as evidenced by improved lung function and reduced collagen deposition. EA modulated gut-lung microbiota by suppressing pro-inflammatory pathogens and enriching immunoregulatory taxa. These changes correlated with reduced serum endotoxemia and inflammation, marked by decreased pro-inflammatory cytokines and increased IL-10 in serum, BALF, and colon tissues. The ameliorated inflammatory environment was further linked to inhibition of EMT in airways, shown by upregulated epithelial markers and downregulated mesenchymal markers. Correlative analyses supported these associations. *Ligilactobacillus* enrichment negatively correlated with serum LPS, while *Mycoplasmopsis* positively associated with inflammation and EMT markers. Sham acupuncture failed to achieve these effects.

**Conclusion:**

Electroacupuncture ameliorates airway remodeling in COPD by modulating gut and lung microbiotareducing inflammation and inhibits EMT, suggesting microbiota regulation as a potential contributor to its therapeutic effects.

## 1 Introduction

Chronic obstructive pulmonary disease (COPD) is a chronic inflammatory airway disorder characterized by persistent airflow limitation and progressive airway remodeling (Christenson et al., 2022; Wei J. et al., 2024). Globally affecting approximately 392 million individuals in 2019 and ranked as the third leading cause of death worldwide, with the mortality burden especially pronounced in South and East Asia (Adeloye et al., 2022). The number of individuals with COPD is projected to approach 600 million by 2050 (Boers et al., 2023). It also imposes a substantial economic burden, with annual medical expenditures for patients being 2.7 times higher than for non-COPD individuals, and global costs are projected to nearly double by 2030 compared to 2010 levels (Naeem et al., 2025). Its pathological features include chronic inflammation with infiltration of neutrophils, macrophages, and lymphocytes, epithelial hyperplasia, goblet cell metaplasia, and subepithelial collagen deposition, ultimately leading to small airway obstruction and fibrosis (Osei et al., 2016). Airway remodeling plays a pivotal role in COPD progression, contributing to irreversible airflow limitation and disease severity (Lu et al., 2021). Central to airway remodeling is epithelial-mesenchymal transition (EMT), a process where airway epithelial cells downregulate junctional proteins (E-cadherin, ZO-1, and occludin) and concomitantly upregulate mesenchymal markers (N-cadherin, vimentin,α-smooth muscle actin, and fibronectin) (Su et al., 2022). This transformation enhances cell migration and invasion, and exacerbates extracellular matrix deposition that culminates in subepithelial fibrosis and small airway obstruction (Wang Y. et al., 2024). In COPD, EMT drives airway remodeling through mechanisms involving cigarette smoke extract and oxidative stress, which activate EMT-related signaling pathways and inducing transcription factors (Snail, Twist), thereby sustaining EMT activation, disrupting the epithelial barrier, promoting airway wall thickening, and influencing airflow limitation and disease severity (Mottais et al., 2023; Wang Y. et al., 2024).

Emerging evidence underscores the critical role of microbial dysbiosis in COPD pathogenesis (Liang et al., 2023; Li R. et al., 2024). For example, COPD patients exhibit a dysbiotic lung microbiome, marked by increased Proteobacteria, compared to healthy individuals (Ramsheh et al., 2021). Similarly, COPD is linked to gut microbiota dysbiosis, with altered composition and reduced abundance of beneficial taxa (Chiu et al., 2021). In contrast, health-associated taxa is less abundant in COPD, whereas pro-inflammatory microbes are enriched (Bowerman et al., 2020). Cigarette smoke (CS), a primary etiological factor, compromises airway integrity, leading to an enrichment of pathogenic Proteobacteria (e.g., *Moraxella*) in the lung microbiota (Li R. et al., 2024). This shift promotes chronic inflammation through inflammatory mediators. Lung diseases often coincide with gut microbiota dysbiosis and associated inflammatory reactions (Ma et al., 2022). Recent studies indicate that the gut microbiota significantly influences CS-induced COPD development (Liu Y. et al., 2024), while the commensal bacterium *Parabacteroides goldsteinii*, isolated from the gut microbiota, ameliorates COPD-related lung inflammation (Lai et al., 2022). Furthermore, gut dysbiosis exacerbates lung inflammation via the gut-lung axis through endotoxin (e.g., LPS) translocation (Lai et al., 2022). Microbiota-derived metabolites, such as short-chain fatty acids (SCFAs), can modulate inflammation and EMT-related pathways in preclinical models (Li et al., 2021). Notably, although EMT exhibits inflammation-dependence and dysbiosis signatures correlate significantly with disease severity (He et al., 2021), the causal mechanisms linking dynamic gut-lung microbiota changes to EMT activation remain unexplored.

Current mainstay therapies, including bronchodilators and corticosteroids, effectively reduce exacerbation frequency, improve lung function, and alleviate dyspnea. However, their efficacy is limited, and they carry potential systemic side effects (e.g., tachycardia, tremor) without significantly impacting mortality (Labaki and Rosenberg, 2020). Critically, these treatments fail to halt or reverse EMT-driven airway remodeling (Liang et al., 2023), and strategies targeting EMT are hampered by poor tissue specificity and the systemic toxicity of molecular inhibitors (Hogg et al., 2017; Higham et al., 2019). This therapeutic impasse highlights the urgent need for novel strategies targeting the microbiota-EMT axis.

Clinically, acupuncture, particularly electroacupuncture (EA), demonstrates promising benefits in managing COPD by improving lung function, reducing dyspnea, alleviating inflammation, and enhancing quality of life (Shi et al., 2024; Li et al., 2025). Recent randomized controlled trials confirm that acupuncture provides not only short-term but also long-term relief for COPD patients, improving dyspnea and suggesting potential for broader effects, including modulation of anti-inflammatory responses, nutritional status, respiratory function, and muscle strength (Suzuki et al., 2025). Moreover, clinical studies indicate that acupuncture can modulate gut microbiota composition, potentially contributing to reduced systemic inflammation in COPD patients (Xu H. et al., 2024; Liu et al., 2025). Preclinical studies further indicate that EA can mitigate airway inflammation and remodeling in COPD models through mechanisms involving the cholinergic anti-inflammatory pathway and dopamine D2 receptor signaling (Liu L. et al., 2024; Shi et al., 2024). The selection of BL13 (Feishu) and ST36 (Zusanli) are supported by neuroanatomical and autonomic evidence implicating somato-autonomic reflex regulation relevant to the gut–lung axis. BL13, located in the upper thoracic paraspinal region, can engage spinal autonomic circuits; experimental data indicate that EA at BL13 recruits a somatosensory–sympathetic pathway relayed via the intermediolateral spinal cord and sympathetic trunk to the lung, attenuating airway inflammation and mucus hypersecretion in a preclinical model (Ma et al., 2025). ST36 has been shown to modulate vagus-dependent anti-inflammatory responses, supporting its role in autonomic - immune regulation (Torres-Rosas et al., 2014). Consistently, animal studies report that EA at these points can partially restore lung and gut microbiota homeostasis and reduce pathogenic taxa in lung disease models. Additionally, EA has been demonstrated to suppress EMT in various models by downregulating mesenchymal markers like vimentin and α-SMA while upregulating epithelial markers, potentially through anti-inflammatory pathways (Liu J. et al., 2023; Yue et al., 2024). Despite these findings, the precise mechanisms by which EA coordinates the complex interplay between gut-lung microbiota imbalance, systemic inflammation, and airway EMT remain undefined.

To address this challenge, this study proposes the central hypothesis that EA alleviates airway remodeling by restoring gut-lung microbiota homeostasis, thereby suppressing EMT and inflammation. We established a COPD rat model induced by CS and LPS, and employed a comprehensive approach integrating assessments of lung function, quantitative analysis of airway collagen deposition, measurement of inflammatory cytokines in multiple compartments (serum, bronchoalveolar lavage fluid, colon tissue), detection of key EMT markers in lung tissue, evaluation of systemic endotoxemia (serum LPS), and 16S rRNA sequencing of lung and gut microbiota combined with PICRUSt2 functional prediction. By characterizing the compartment-specific modulatory effects of EA on microbiota and assessing its impact on the gut-lung microbiota-EMT axis, this study provides evidence suggesting a link between EA-associated modulation of the gut–lung microbiota and changes in lung EMT in COPD airway remodeling. By exploring the interconnections among gut-lung microbiota, lung EMT, lung function, and inflammation, this research aims to clarify the therapeutic potential of EA in COPD airway remodeling.

## 2 Materials and methods

### 2.1 Animals and groups

Twenty-four male healthy specific-pathogen-free (SPF) grade Sprague Dawley (SD) rats (6 weeks old, 230–250 g) were procured from Chengdu Dashuo Laboratory Animal Co., Ltd., [Sichuan, China; Laboratory Animal Production License No. SCXK (chuan) 2020-0030]. The animals were housed in the specific-pathogen-free facility of Chengdu University of Traditional Chinese Medicine [Institutional Animal Use Permit No. SYXK (chuan) 2020-124] under controlled environmental conditions: 12-h light/dark cycle, ambient temperature maintained at 22 °C ± 1 °C, relative humidity 50% ± 5%, with *ad libitum* access to standard rodent chow and filtered water. Following a 7-days acclimatization period, rats were randomly assigned to four experimental groups (*n* = 6/group): Control (Con), COPD, EA, and sham-acupuncture (SA). All procedures were conducted in accordance with the Guidelines for the Care and Use of Laboratory Animals and approved by the Laboratory Animal Welfare Ethics Committee of Chengdu University of Traditional Chinese Medicine (Ethics Review No. 2024006).

### 2.2 Preparation of COPD model rats

The COPD rat model was established using combined CS exposure and LPS induction, as previously described (Liu Y. et al., 2024). All rats except those in the Con group were placed in the smoke chamber of an Animal Fumigation Modeling System (CSM-100C, Tow-Int Tech, Shanghai, China) and exposed to smoke from 15 cigarettes (Jiaozi brand: tar 11 mg, nicotine 1.1 mg, carbon monoxide 11 mg; Sichuan Tobacco Industry Co., China) per session. The CS exposure protocol consisted of 1-h sessions twice daily, 6 days per week for 84 days. On days 1, 31, and 61, rats received intratracheal injections of 200 μL LPS (1 mg/mL, L2880, Sigma, St. Louis, USA) without subsequent CS exposure on those days. Rats in the Con group were exposed to CS-free air for 84 days and administered intratracheal injections of saline (equal volume) at corresponding time points. Model validation was based on assessment of lung function parameters and histopathological changes in lung tissues.

### 2.3 EA and SA treatment

Electroacupuncture procedures adhered to internationally recognized guidelines for animal acupuncture (Liu S. et al., 2021), ensuring minimal physiological perturbation and rapid post-procedural recovery. During EA and SA procedures, rats were anesthetized using inhaled isoflurane delivered via a small-animal inhalation anesthesia machine (R500, RWD Life Science, China). Anesthesia was induced with 2%–3% isoflurane in oxygen through an induction chamber and maintained at 1%–1.5% using a nose cone connected to the vaporizer. The depth of anesthesia was monitored by respiratory patterns and the absence of pedal withdrawal reflex. All groups underwent identical anesthesia procedures to minimize physiological variability. Following our validated protoco (Liu L. et al., 2024), bilateral “Feishu” (BL13: 6 mm lateral to the dorsal midline at the third thoracic intercostal space) and “Zusanli” (ST36: 3 mm inferior to the fibular head on the posterolateral knee) were selected as primary acupoints. To enhance stimulation efficacy, an auxiliary needle (identical specifications: 0.30 mm × 25 mm, Suzhou Medical Supplies Co., Ltd., China) was inserted 2 mm lateral to each primary acupoint (BL13 and ST36) at matching depths (6 mm for BL13; 7 mm for ST36). Electrical stimulation was applied between primary and auxiliary needles within the same acupoint using an EA device (SDZ-V, Huatuo, Suzhou, China), with the anode connected to the primary needle and cathode to the auxiliary needle (Liu L. et al., 2024). Alternating daily between contralateral acupoints, dense-disperse waves (4/20 Hz frequency, 1–3 mA intensity) were applied for 20 min/day over 14 days, adjusted to induce mild hindlimb tremor without vocalization. SA procedures followed Professor Liu’s established protocol (Yang et al., 2020): two subcutaneous insertions of identical needles were performed at a non-acupoint (midpoint above the tail base), angled 15° toward the tail tip, with no electrical stimulation and no manual manipulation. All Con and COPD group rats underwent equivalent anesthesia protocols without needle intervention.

Rationale for stimulation parameters: The dense-sparse waveform (4/20 Hz) was selected because mixed-frequency stimulation may produce broader neurochemical engagement and synergistic endogenous opioid release than single-frequency protocols (Xu J. et al., 2024). This choice was supported by our prior COPD rat study using the same acupoints (BL13 and ST36), which applied 4/20 Hz for 20 min/day and compared 1 vs. 3 mA, demonstrating feasibility and reproducibility of this parameter set in a cigarette smoke-induced COPD model (Liu L. et al., 2024). Neuroanatomical evidence further supports stabilizing frequency/duration while strictly controlling current intensity to ensure cross-experiment comparability (Liu et al., 2020). Accordingly, we used the validated frequency/duration and titrated the current (1–3 mA) to evoke mild local twitching (hindlimb tremor) without distress, balancing efficacy and tolerability.

### 2.4 Euthanasia

At the experimental endpoint, rats were deeply anesthetized with sodium pentobarbital (150 mg/kg, intraperitoneal) (Mohamed et al., 2020). After the complete loss of pedal withdrawal reflex was confirmed, euthanasia was performed by exsanguination during tissue collection.

### 2.5 Lung function measurements

Lung function was assessed using the AniRes2005 Animal Lung Function System (Beijing Beilanbo Technology Co., Ltd., Beijing, China). Rats were anesthetized intraperitoneally with 1% sodium pentobarbital (40 mg/kg, P3761, Sigma-Aldrich, St. Louis, MO, USA) and secured in supine position. Following blunt dissection of cervical tissues, the trachea was exposed and cannulated with a Y-shaped endotracheal tube connected to the system. Key spirometric parameters were recorded, including the ratio of forceful lung volume (FVC) to 0.1-s forceful expiratory volume (FEV_0_._1_) (FEV_0_._1_/FVC), and FVC was measured against 0.3-s forceful expiratory volume (FEV_0_._3_) (FEV_0_._3_/FVC) for comparative analysis (Liu L. et al., 2024).

### 2.6 Hematoxylin and eosin staining and Masson staining

Following thoracic and abdominal cavity dissection, lung and colon tissues were harvested. Colon specimens were flushed with 0.9% saline to remove luminal contents. All tissues were immediately fixed in 4% paraformaldehyde for ≥24 h. Fixed lung tissues underwent standardized processing: trimming, graded ethanol dehydration, xylene clearing, paraffin embedding, and sectioning (4–6 μm thickness). Sections were deparaffinized for hematoxylin and eosin (H&E) staining and Masson’s trichrome staining following manufacturer protocols. Histopathological evaluation was performed under light microscopy (×100, ×200 and ×400 magnification) with quantitative analysis of collagen volume fraction via Masson-stained sections using image analysis software (Ding et al., 2024; Wang J. et al., 2024).

### 2.7 Enzyme-linked immunosorbent assay

Following lung function assessment, approximately 5 mL of blood was collected first via abdominal aortic puncture using heparinized syringes and centrifuged (3,000 rpm, 10 min, 4 °C) to obtain serum. Subsequently, bronchoalveolar lavage was performed by slowly infusing 3 mL phosphate-buffered saline (PBS) through the tracheal cannula. The lavage fluid was collected in sterile microcentrifuge tubes and centrifuged (1,000 rpm, 15 min, 4 °C) to obtain supernatants. Colon tissues were gently flushed with 0.9% saline to remove luminal contents. Cytokine concentrations of IFN-γ (Cat# ZC-36294), IL-10 (Cat# ZC-36379), and IL-1β (Cat# ZC-36391) in serum, BALF, and colon homogenates, along with serum LPS levels (Cat# ZC-37600), were quantified using species-specific ELISA kits (Zhuocai Biotechnology Co., Ltd., Shanghai, China) following the manufacturer’s instructions (Wang J. et al., 2024).

### 2.8 Western blotting

Lung tissues were homogenized in RIPA lysis buffer, and supernatants were collected following centrifugation. Protein concentrations were determined using BCA assay, with subsequent denaturation at 95 °C for 5 min. Equal protein loads (30 μg/lane) were separated by SDS-PAGE (12% gel) and transferred to PVDF membranes (0.45 μm pore size). Membranes were blocked with 5% non-fat milk in TBST for 2 h at room temperature, then incubated with primary antibodies at 4 °C overnight: E-cadherin (1:2,000, ServiceBio, Cat# GB11082), N-cadherin (1:2,000, BIOSS, Cat# bs-1172R), Vimentin (1:20,000, Proteintech, Cat# 10366-1-AP), ZO-1 (1:1,000, ServiceBio, Cat# GB111402), and β-actin (1:50,000, Abclonal, Cat# AC026) as loading control. After three 10-min TBST washes, membranes were probed with HRP-conjugated secondary antibodies: goat anti-mouse (1:8,000, Abclonal, Cat# AS003) and goat anti-rabbit (1:8,000, Affinity, Cat# S0001) for 1.5 h at room temperature. Following three additional TBST washes, protein signals were developed using ECL Ultra-sensitive substrate (Biosharp, Cat# BL520B) and quantified through densitometric analysis with ImageJ software (Mohamed et al., 2020).

### 2.9 Quantitative reverse transcription polymerase chain reaction

Lung tissues were rapidly harvested after euthanasia, flash-frozen in liquid nitrogen, and stored at −80 °C. Total RNA was isolated from ∼15 mg of lung tissue using RNAiso Plus Kit (YEASEN, #19221ES50) following tissue homogenization. RNA purity (A260/A280 > 1.8) were verified by NanoDrop (Thermo Fisher). 1 μg total RNA was reverse-transcribed using PrimeScript RT Master Mix (Takara Bio, #RP047A) in 20 μL reactions. Quantitative PCR was performed on a QuantStudio 3 System (Thermo Fisher Scientific) using TB Green Premix Ex Taq II (Takara Bio, Cat#RR820A) in 20 μL reactions containing 3 μL cDNA, 10 μL SYBR Green mix, 0.8 μL each of forward/reverse primers (10 μM), and 5.4 μL nuclease-free water. Primer sequences (5′→3′) were: β-actin (F: GGGAAATCGTGCGTGACATT, R: GCGGCAGTGGCCATCTC), E-cadherin (F: GTCAGATCAGGACCAGGACTACG, R: TCTTCGCCGCCACCATAC), N-cadherin (F: AGATACCGTGG AGCTTGATGC, R: TGCGGATCGGACTGGATACTG), Vimentin (F: GCAGGACTCCGTGGACTTCTC, R: GTAGT TGGCGAAGCGGTCATTC), ZO-1 (F: GCATGATGATCGT CTGTCCATCC, R: CCGCCTTCTGTATCTGTGTCTTC). Relative mRNA expression was normalized to β-actin using the 2^–ΔΔCt^ method (Mrkvicova et al., 2019).

### 2.10 Lung and gut microbiota analysis

Total DNA was extracted from bronchoalveolar lavage fluid (BALF) and fecal samples of all groups. The V3–V4 hypervariable regions of the bacterial 16S rRNA gene were amplified by PCR (primers 338F: ACTCCTACGGGAGGCAGCAG, 806R: GGACTACHVGGGTWTCTAAT) and verified by 2% agarose gel electrophoresis. Paired-end sequencing was performed on the Illumina NovaSeq 6000 platform (Illumina, San Diego, USA) with 250 bp read length. Raw data were processed through QIIME2 for quality filtering, denoising, and operational taxonomic unit (OTU) clustering (97% similarity threshold). Alpha diversity and beta diversity analyses were conducted. Taxonomic composition at phylum and genus levels was compared using ANCOM-BC. Functional prediction of microbiota was performed via PICRUSt2 with KEGG pathway annotation, and differentially enriched pathways [Linear discriminant analysis Effect Size (LEfSe), linear discriminant analysis (LDA) score > 3.5] were identified. Spearman’s rank correlation analysis (FDR-corrected *p* < 0.05) was applied to assess associations between lung and gut microbial taxa (Li et al., 2020).

### 2.11 Data and statistical analysis

Statistical analyses were performed using IBM SPSS Statistics 26.0 (Armonk, NY, USA) and visualized with GraphPad Prism 9.0 (San Diego, CA, USA). Continuous variables were expressed as mean ± standard deviation (SD). Normality and homogeneity of variance were assessed through Shapiro-Wilk and Levene’s tests, respectively. Parametrically distributed data were analyzed by one-way ANOVA with Tukey’s *post-hoc* test, while non-parametric datasets underwent Kruskal-Wallis test with Dunn’s correction. Gut microbiota analyses were conducted on the BMKCloud platform (Biomarker Technologies, Beijing, China).^1^ Spearman rank correlations (two-tailed, false discovery rate adjusted) were used to assess associations between lung function, inflammation, airway EMT indices and different microbial taxa. Statistical significance was defined as adjusted *P* < 0.05.

## 3 Results

### 3.1 EA attenuates lung dysfunction and airway remodeling in COPD

**FIGURE 1:**
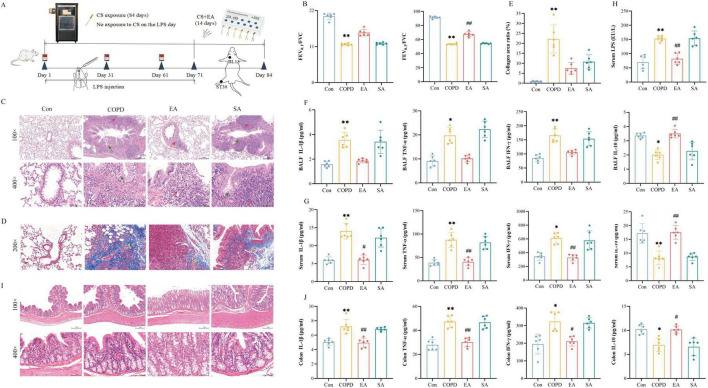
Electroacupuncture (EA) ameliorates CS/LPS-induced lung dysfunction, tissue injury, and systemic inflammation in COPD rats. **(A)** Schematic of experimental modeling and intervention timeline. **(B)** Spirometric parameters (FEV_0.1_/FVC and FEV_0.3_/FVC ratios). **(C)** Representative H&E-stained lung sections: green arrows indicate peribronchial lymphoid hyperplasia, red arrows denote goblet cell metaplasia. **(D,E)** Masson trichrome staining of lung tissue (yellow colored arrows represent collagen deposition) and quantitative collagen volume fraction. **(F–H)** Pro-/anti-inflammatory cytokine levels in BALF and serum (IL-1β, TNF-α, IFN-γ, IL-10) and serum LPS concentrations. **(I,J)** H&E-stained colon sections (no pathological damage observed) and colonic cytokine profiles. Data expressed as mean ± SD; *P < 0.05, ^**^P < 0.01 vs. Con; ^#^*P* < 0.05, ^##^*P* < 0.01 vs. COPD; *n* = 6/group.

Electroacupuncture significantly improved lung function and attenuated airway remodeling in CS/LPS-induced COPD rat (Figure 1A).

Compared to control group, COPD rats exhibited severe airflow limitation evidenced by decreased FEV_0_._1_/FVC (11.67 ± 0.13 vs. 20.25 ± 0.40, *P* < 0.01) and FEV_0_._3_/FVC (53.71 ± 0.45 vs. 91.44 ± 0.66, *P* < 0.01) in COPD versus Con groups, indicating severe airflow limitation (Figure 1B). Histopathological analysis confirmed characteristic COPD pathology including peribronchial lymphoid hyperplasia, epithelial metaplasia with goblet cell proliferation (Figure 1C), and elevated collagen deposition (22.19% ± 2.92% vs. 0.92% ± 0.10%, *P* < 0.01) (Figures 1D,E). Following EA intervention, significant improvements were observed: FEV_0_._1_/FVC (15.25 ± 0.41) and FEV_0_._3_/FVC (67.56 ± 1.43) ratios (*P* < 0.01 vs. COPD), reducing inflammatory infiltrates to near baseline levels, and decreasing fibrosis (7.53% ± 1.18%, *P* < 0.01 vs. COPD). In contrast, SA group showed no significant therapeutic effect, with FEV_0_._1_/FVC at 11.93 ± 1.14, FEV_0_._3_/FVC at 54.46 ± 0.27, and fibrosis at 10.76% ± 1.45% (*P* > 0.05 vs. COPD). These findings demonstrate that EA significantly attenuates COPD-associated functional impairment and structural remodeling, with effects superior to sham acupuncture.

### 3.2 EA regulates lung, intestinal, and serum inflammation in COPD

Electroacupuncture significantly attenuated inflammatory responses across lung, systemic, and intestinal compartments in COPD rats (Figures 1F,G). In BALF (Figure 1F), the COPD group exhibited elevated pro-inflammatory cytokines compared to controls: IL-1β (3.56 ± 0.25 vs. 1.58 ± 0.97 pg/mL, *P* < 0.01), TNF-α (19.74 ± 1.33 vs. 9.12 ± 0.88 pg/mL, *P* < 0.05), and IFN-γ (165.65 ± 9.62 vs. 85.77 ± 5.04 pg/mL, *P* < 0.01), alongside reduced anti-inflammatory IL-10 (2.02 ± 0.14 vs. 3.36 ± 0.08 pg/mL, *P* < 0.05). EA intervention significantly reduced these pro-inflammatory mediators (IL-1β: 1.87 ± 0.07; TNF-α: 10.17 ± 0.56; IFN-γ: 105.59 ± 3.32) and increased IL-10 (3.51 ± 0.12, *P* < 0.01), whereas SA showed no significant changes.

Systemically, serum analysis revealed parallel alterations: COPD rats demonstrated increased IL-1β (14.03 ± 0.89 vs. 5.95 ± 0.39 pg/mL in controls, *P* < 0.01), TNF-α (87.60 ± 6.57 vs. 38.76 ± 2.67 pg/mL, *P* < 0.01), IFN-γ (610.53 ± 31.46 vs. 345.80 ± 22.92 pg/mL, *P* < 0.05), and decreased IL-10 (8.28 ± 1.03 vs. 17.24 ± 1.47 pg/mL, *P* < 0.01). EA treatment effectively normalized these perturbations (IL-1β: 6.09 ± 0.53; TNF-α: 40.60 ± 3.48; IFN-γ: 327.95 ± 19.48; IL-10: 17.54 ± 1.01; all *P* < 0.01 vs. COPD) (Figure 1G). Consistent with cytokine profiles, serum LPS levels were markedly elevated in COPD rats (152.94 ± 3.89 vs. 70.69 ± 9.84 EU/mL in controls, *P* < 0.01), which EA significantly attenuated (82.05 ± 7.70 EU/mL, *P* < 0.01) while SA had no effect (154.41 ± 10.42 EU/mL, *P* > 0.05) (Figure 1H).

Notably in colon tissue, despite absence of histopathological damage (Figure 1I), pro-inflammatory cytokines were significantly elevated in COPD rats versus controls: IL-1β (7.21 ± 0.40 vs. 5.05 ± 0.18 pg/mL, *P* < 0.01), TNF-α (47.22 ± 1.94 vs. 27.87 ± 1.82 pg/mL, *P* < 0.01), IFN-γ (323.02 ± 22.30 vs. 195.55 ± 23.19 pg/mL, *P* < 0.05), and reduced IL-10 (6.96 ± 0.57 vs. 10.24 ± 0.41 pg/mL, *P* < 0.05). EA treatment effectively reversed these alterations (IL-1β: 4.96 ± 0.26, *P* < 0.01; TNF-α: 30.18 ± 1.53, *P* < 0.01; IFN-γ: 211.84 ± 11.26, *P* < 0.05; IL-10: 10.18 ± 0.31 pg/mL, *P* < 0.05; vs. COPD) (Figure 1J).

### 3.3 EA suppresses EMT in COPD airways

**FIGURE 2:**
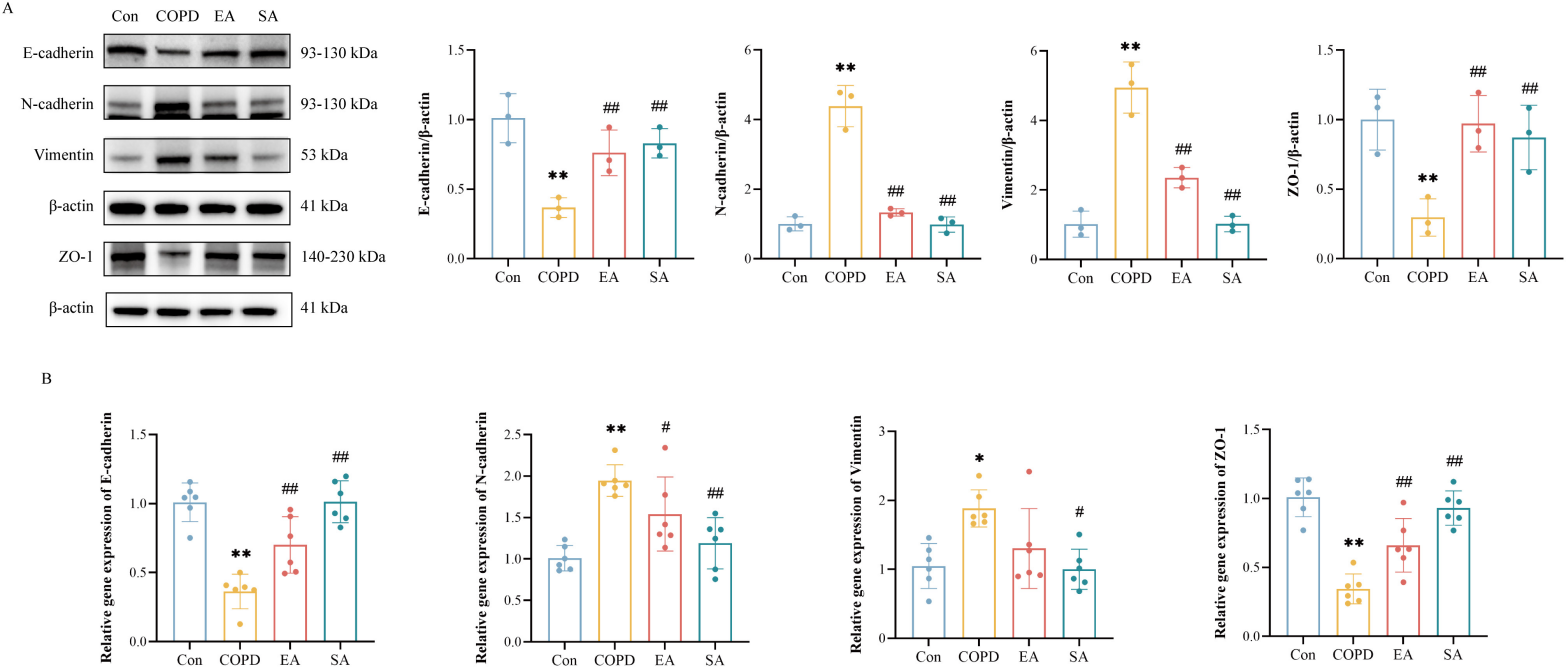
Electroacupuncture (EA) suppresses airway epithelial-mesenchymal transition (EMT) in COPD rats. **(A)** Western blot analysis of EMT markers in lung tissues: epithelial markers (E-cadherin, ZO-1) and mesenchymal markers (N-cadherin, vimentin). Representative bands and quantified protein expression normalized, with band intensities normalized to β-actin. **(B)** qPCR analysis of EMT-related gene expression in lung tissues. Data expressed as mean ± SD; *P < 0.05, ^**^P < 0.01 vs. Con; ^#^*P* < 0.05, ^##^*P* < 0.01 vs. COPD; *n* = 6/group.

As shown in Figure 2, COPD rats exhibited significantly downregulated epithelial markers versus controls: E-cadherin protein (0.37 ± 0.04 vs. 1.01 ± 0.10, *P* < 0.01) and mRNA (0.36 ± 0.05 vs. 1.00 ± 0.06, *P* < 0.01), ZO-1 protein (0.30 ± 0.08 vs. 1.00 ± 0.13, *P* < 0.01) and mRNA (0.34 ± 0.04 vs. 1.00 ± 0.06, *P* < 0.01). Both EA and SA partially restored these markers (EA: E-cadherin protein 0.76 ± 0.09, mRNA 0.70 ± 0.08; ZO-1 protein 0.97 ± 0.12, mRNA 0.66 ± 0.08; all *P* < 0.01 vs. COPD. SA: E-cadherin protein 0.83 ± 0.06, mRNA 1.01 ± 0.06; ZO-1 protein 0.87 ± 0.13, mRNA 0.93 ± 0.05, all *P* < 0.01 vs. COPD). Conversely, mesenchymal markers were upregulated in COPD: N-cadherin protein (4.38 ± 0.34 vs. 1.00 ± 0.12, *P* < 0.01) and mRNA (1.94 ± 0.08 vs. 1.00 ± 0.06, *P* < 0.01), vimentin protein (4.95 ± 0.43 vs. 1.02 ± 0.22, *P* < 0.01) and mRNA (1.88 ± 0.11 vs. 1.05 ± 0.13, *P* < 0.05). EA demonstrated superior efficacy in normalizing these alterations (N-cadherin protein 1.33 ± 0.06, *P* < 0.01, mRNA 1.54 ± 0.18, *P* < 0.05; vimentin protein 2.35 ± 0.17, *P* < 0.01, mRNA 1.30 ± 0.24; vs. COPD) compared to SA (N-cadherin protein 0.98 ± 0.13, *P* < 0.01, mRNA 1.19 ± 0.13, *P* < 0.01; vimentin protein 1.02 ± 0.13, *P* < 0.01, mRNA 1.00 ± 0.12, *P* < 0.05; vs. COPD).

These findings demonstrate that EA mitigates COPD-associated EMT progression, as evidenced by restoration of epithelial markers (E-cadherin, ZO-1) and suppression of mesenchymal markers (N-cadherin, vimentin), thereby attenuating airway remodeling.

### 3.4 EA modulates microbial diversity and composition in lung and gut microbiota

#### 3.4.1 Alpha and beta diversity of lung and gut microbiota

**FIGURE 3:**
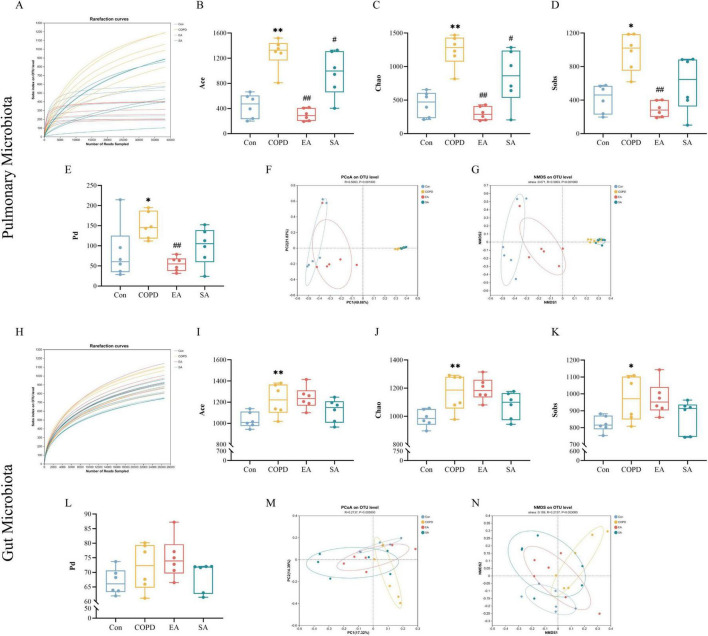
Electroacupuncture (EA) modulates microbial diversity in lung and gut microbiota of COPD rats. **(A)** Rarefaction curves of lung microbiota based on observed species (Sobs), indicating adequate sequencing depth. **(B–E)** α-Diversity indices of lung microbiota: ACE **(B)**, Chao1 **(C)**, Sobs **(D)**, and phylogenetic diversity (Pd) **(E)**. **(F,G)** β-Diversity analysis of lung microbiota via Bray-Curtis distance-based principal coordinates analysis (PCoA, **F**) and non-metric multidimensional scaling (NMDS, **G**). **(H)** Rarefaction curves of gut microbiota (Sobs-based). **(I–L)** Gut microbiota α-diversity indices: ACE **(I)**, Chao1 **(J)**, Sobs **(K)**, and Pd **(L)**. **(M,N)** β-Diversity ordination of gut microbiota using PCoA **(M)** and NMDS **(N)**. Data expressed as mean ± SD; *P < 0.05, ^**^P < 0.01 vs. Con; ^#^*P* < 0.05, ^##^*P* < 0.01 vs. COPD; *n* = 6/group.

All 24 lung and 24 gut microbiota samples (6 per group) passed quality control, with rarefaction curves indicating adequate sequencing depth (Figures 3A,H).

In lung microbiota, COPD rats showed higher α-diversity than controls: ACE (1280.83 ± 100.59 vs. 440.12 ± 78.20, *P* < 0.01), Chao1 (1238.94 ± 96.51 vs. 440.88 ± 76.67, *P* < 0.01), observed species (Sobs) (972.17 ± 92.31 vs. 417.00 ± 68.58, *P* < 0.05), and phylogenetic diversity (Pd) (150.48 ± 13.73 vs. 82.81 ± 28.07, *P* < 0.05). EA intervention decreased these indices (ACE: 299.78 ± 39.83; Chao1: 301.88 ± 41.13; Sobs: 293.17 ± 38.28; Pd: 54.28 ± 7.31; all *P* < 0.01 vs. COPD), while SA induced no significant changes (Figures 3B–E). β-Diversity analysis revealed that EA shifted lung microbiota composition toward control-like patterns, whereas COPD and SA groups clustered together (Figures 3F,G).

Gut microbiota analysis demonstrated parallel trends (Figures 3I–L): COPD-induced α-diversity elevation (ACE: 1221.60 ± 60.61 vs. 1030.33 ± 30.23, *P* < 0.01; Chao1: 1167.09 ± 54.13 vs. 988.07 ± 24.93, *P* < 0.01; Sobs: 970.17 ± 54.98 vs. 822.67 ± 19.13, *P* < 0.05; Pd: 71.84 ± 3.25 vs. 66.87 ± 1.83) was attenuated by both EA (ACE: 1241.22 ± 43.06; Chao1: 1192.79 ± 33.37; Sobs: 971.83 ± 39.91; Pd: 74.89 ± 2.89) and SA (ACE: 1121 ± 44.30; Chao1: 1077.68 ± 38.90; Sobs: 867.83 ± 39.73; Pd: 68.69 ± 2.05; *P* < 0.05). However, β-diversity ordination (PCoA: Figure 3M; NMDS: Figure 3N) demonstrated that only EA restructured gut microbiota toward control distributions, with SA showing minimal alterations.

#### 3.4.2 Taxonomic composition analysis of lung and gut microbiota

**FIGURE 4:**
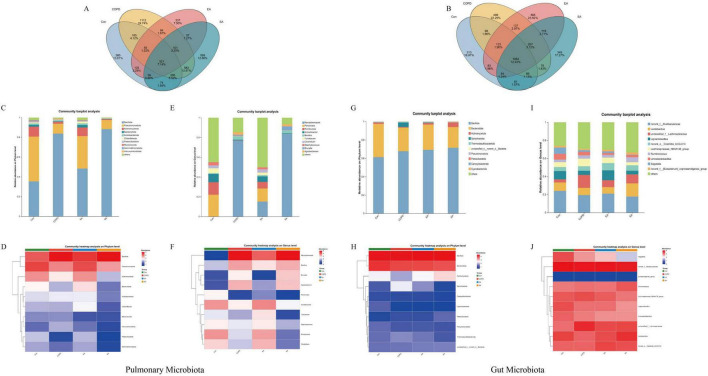
Electroacupuncture (EA) modulates taxonomic composition of lung and gut microbiota in COPD rats. **(A)** Venn diagram of lung microbiota showing shared operational taxonomic units (OTUs) among groups. **(B)** Venn diagram of gut microbiota OTU distribution. **(C,D)** Relative abundance of lung microbiota at phylum level: stacked bar chart **(C)** and heatmap **(D)**. **(E,F)** Genus-level composition of lung microbiota: stacked bar chart **(E)** and heatmap **(F)**. **(G,H)** Gut microbiota phylum-level abundance: stacked bar chart **(G)** and heatmap **(H)**. **(I,J)** Gut microbiota genus-level distribution: stacked bar chart **(I)** and heatmap **(J)**. Data are presented as relative abundance (%); *n* = 6 per group.

Venn diagrams revealed intersample similarities in microbial composition. For lung microbiota (Figure 4A), EA shared 103 operational taxonomic units (OTUs) with Con and 84 with COPD, whereas SA shared 74 OTUs with Con and 583 with COPD, indicating greater compositional similarity between EA-Con and SA-COPD pairs. In gut microbiota (Figure 4B), EA shared 83 OTUs with Con and 137 with COPD, compared to SA’s 61 shared with Con and 78 with COPD, demonstrating EA’s stronger structural resemblance to healthy controls.

Dominant taxa were analyzed at phylum and genus levels. Lung microbiota: The top five phyla (Figure 4C)–Bacillota, Pseudomonadota, Actinomycetota, Bacteroidota and Acidobacteriota–accounted for >96% of sequences. Key genera (Figure 4E) included *Mycoplasmopsis*, *Pandoraea*, *Romboutsia*, *Acinetobacter* and *Bacillus*, constituting >97% abundance. EA treatment significantly increased Pseudomonadota, Actinomycetota, Bacteroidota, Acidobacteriota, *Pandoraea*, *Romboutsia* and *Acinetobacter*, while reducing Bacillota, *Mycoplasmopsis* and *Bacillus* versus COPD (Figures 4D,F). Gut microbiota: Dominant phyla (Figure 4G) were Bacillota, Bacteroidota, Actinomycetota, Spirochaetota, and Thermodesulfobacteriota, representing >99% sequences. Major genera (Figure 4I) included *norank_f_Muribaculaceae*, *Lactobacillus*, unclassified_f_Lachnospiraceae, *Ligilactobacillus*, and *norank_o_Clostridia_UCG-014*, collectively >50% abundance. EA upregulated Bacteroidota, *norank_f_Muribaculaceae* and *Ligilactobacillus*, while suppressing Spirochaetota and unclassified_f_Lachnospiraceae compared to COPD (Figures 4H,J).

#### 3.4.3 Differential microbial analysis of lung and gut microbiota

**FIGURE 5:**
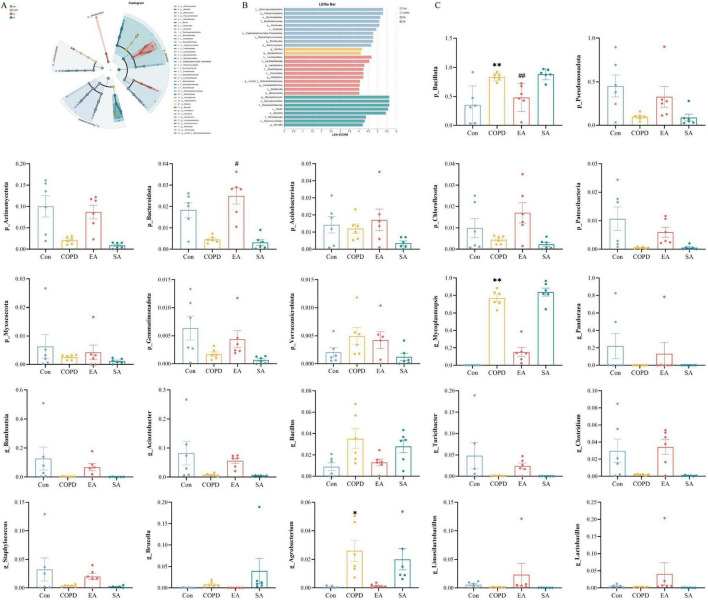
Effects of EA on lung microbiota through LEfSe analysis and differential taxa at phylum/genus levels. **(A)** Cladogram showing phylogenetic distribution of discriminant taxa. **(B)** Histogram of LDA scores for significantly enriched taxa (LDA > 4.0). **(C)** Comparative analysis of differentially abundant phyla and genera among groups. Data expressed as mean ± SD; *P < 0.05, ^**^P < 0.01 vs. Con; ^#^*P* < 0.05, ^##^*P* < 0.01 vs. COPD; *n* = 6/group.

**FIGURE 6:**
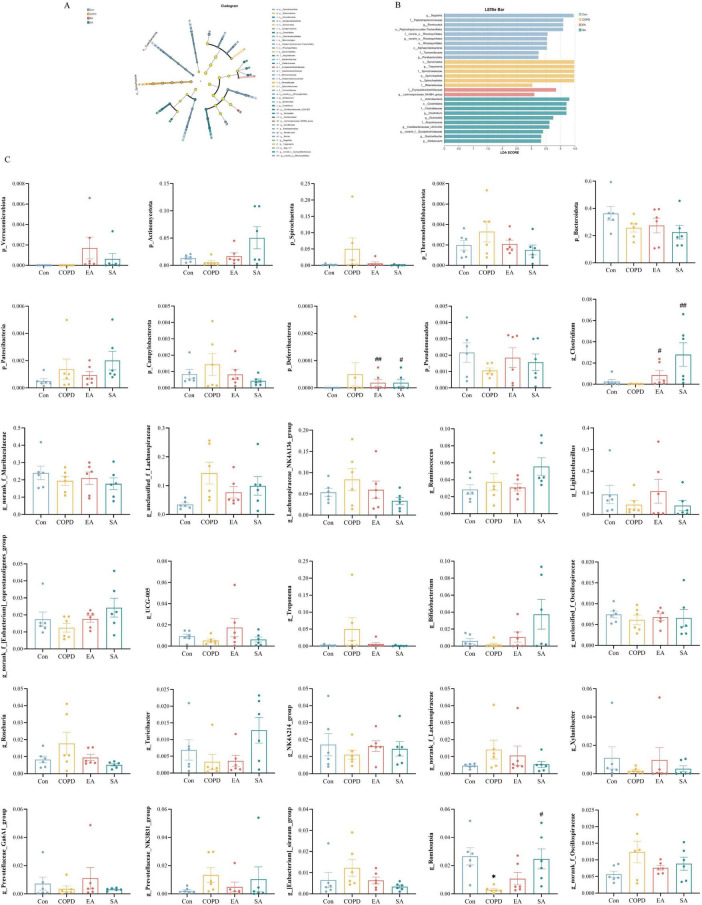
Effects of EA on gut microbiota through LEfSe analysis and differential taxa at phylum/genus levels. **(A)** Cladogram of gut microbial phylogenetic features. **(B)** LDA score distribution of gut microbial biomarkers (LDA > 2.0). **(C)** Group-wise comparison of differentially abundant phyla and genera. Data expressed as mean ± SD; *P < 0.05 vs. Con; ^#^*P* < 0.05, ^##^*P* < 0.01 vs. COPD; *n* = 6/group.

Linear discriminant analysis Effect Size analysis with compartment-specific stringency (lung LDA > 4.0, gut LDA > 2.0) identified distinct microbial biomarkers. In lung microbiota, Pseudomonadota characterized controls while *Bacillus* dominated COPD, with Lactobacillales and *Mycoplasmopsis* marking EA and SA groups respectively (Figures 5A,B). Gut microbiota featured *Segatella* in controls, *Treponema*/Spirochaetaceae in COPD, *Lachnospiraceae_NK4B4_group* in EA, and *Clostridium* in SA (Figures 6A,B).

Electroacupuncture induced distinct microbial restructuring across lung and intestinal compartments in COPD rats. In lung microbiota at the phylum level (Figure 5C), COPD rats showed significantly increased abundance of Bacillota (*P* < 0.01) and elevated Verrucomicrobiota (*P* < 0.05) compared to controls, alterations that were significantly attenuated by EA intervention. For gut microbiota at the phylum level (Figure 6C), while no statistically significant differences were observed (*P* > 0.05), COPD rats exhibited numerical increases in Spirochaetota and Thermodesulfobacteriota that showed partial reversal following EA treatment.

Genus-level analysis further demonstrated COPD-associated increases in lung *Mycoplasmopsis* (*P* < 0.01) and *Agrobacterium* (*P* < 0.05), contrasted with decreased *Romboutsia* (*P* > 0.05). In parallel, gut microbiota displayed elevated *Treponema* (*P* > 0.05) and reduced *Ligilactobacillus* (*P* > 0.05). EA consistently counteracted these pathological shifts, significantly reducing the elevated genera (*Mycoplasmopsis*, *Agrobacterium*, *Treponema*) while restoring depleted taxa (*Romboutsia*, *Ligilactobacillus*) toward control levels, demonstrating compartment-specific modulation of key microbiota (Figures 5C, 6C).

#### 3.4.4 Functional prediction of microbiota metabolic pathways

Functional prediction analysis of lung and gut microbiota was conducted using Phylogenetic Investigation of Communities by Reconstruction of Unobserved States (PICRUSt) to infer Kyoto Encyclopedia of Genes and Genomes (KEGG) and Clusters of Orthologous Groups (COG) pathways from sequenced genomes, aiming to identify potential functional alterations in microbial communities of the lungs and intestines.

**FIGURE 7:**
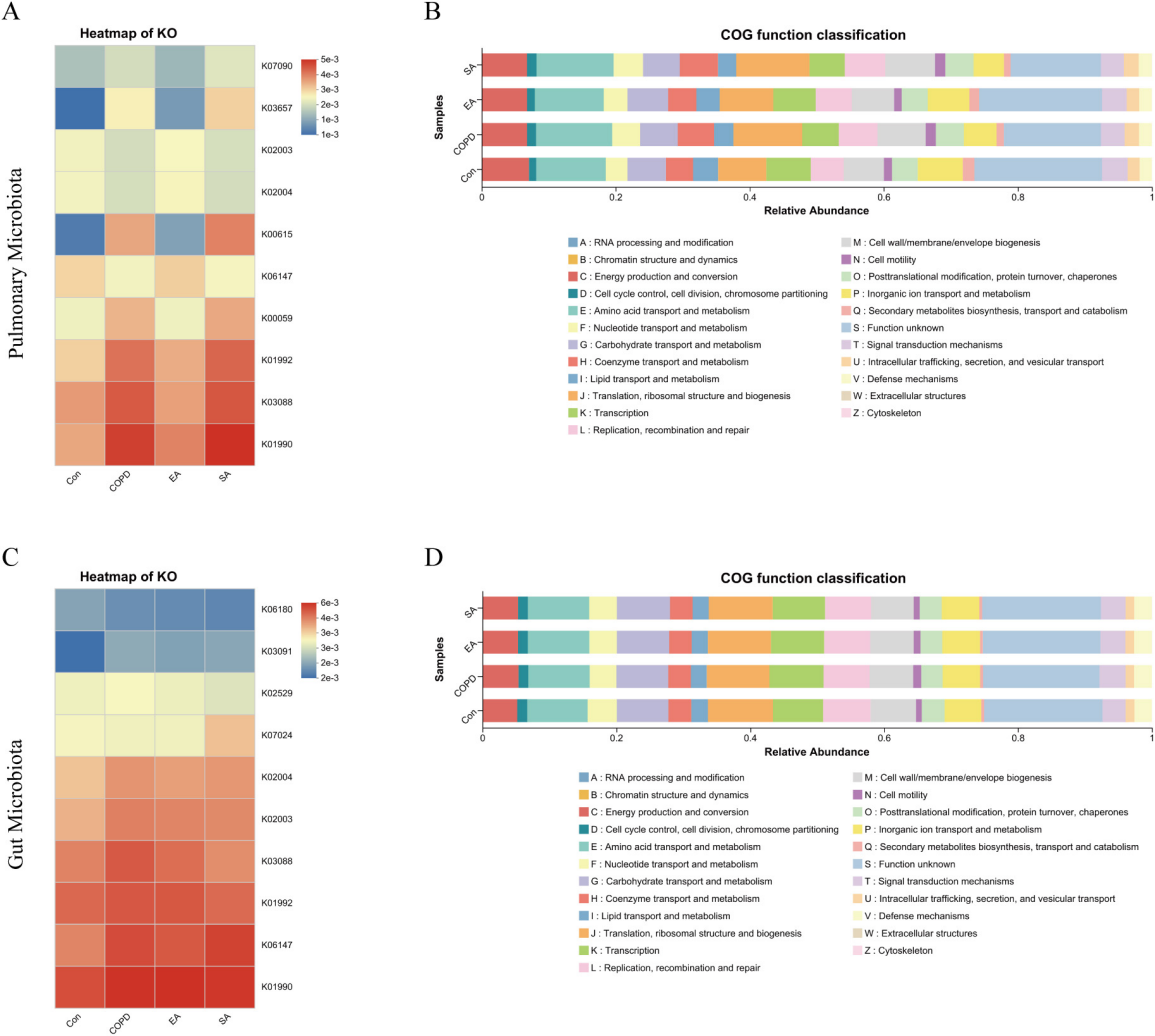
Functional prediction of lung and gut microbiota based on KEGG and COG analyses. **(A)** KEGG functional prediction of lung microbiota. **(B)** COG functional prediction of lung microbiota. **(C)** KEGG functional prediction of gut microbiota. **(D)** COG functional prediction of gut microbiota.

In lung microbiota, KEGG analysis demonstrated significant COPD-associated upregulation of pathogenic pathways including K01990 (ABC transporter ATP-binding protein), K03088 (DNA-directed RNA polymerase subunit), K01992 (lipid A biosynthesis), K00059 (glutathione reductase), K03657 (exonuclease), and K07090 (peptidase) versus controls, all of which were significantly downregulated by EA intervention but unaffected by SA. Conversely, pathways associated with K06147 (fatty acid β-oxidation), K00615 (acetyl-CoA carboxylase), K02004 (iron ABC transporter permease), and K02003 (manganese transporter) were suppressed in the COPD but restored by EA (Figure 7A). In gut microbiota, COPD showed elevated K06147 (fatty acid β-oxidation), K03088 (DNA-directed RNA polymerase subunit), K02004 (iron ABC transporter permease), and K03091 (DNA gyrase subunit B), which EA significantly reduced to control levels while SA induced no significant changes (Figure 7C).

Functional prediction of lung microbiota based on the COG database demonstrated significant alterations in defense-related genes (V), signal transduction mechanisms (T), cell motility (N), and secretion systems (U) in the COPD group (Figure 7B). For gut microbiota, COG-based functional prediction revealed significant changes in carbohydrate (G), amino acid (E), coenzyme (H), and lipid (I) metabolism, ribosomal structure (J), transcription (K), cell wall biogenesis (M), and secondary metabolite (Q) biosynthesis in the COPD group (Figure 7D). EA restored these pathways, contrasting with SA’s limited efficacy.

### 3.5 EA modulates gut-lung microbiota correlations with pathological indices in COPD

#### 3.5.1 Dysregulated gut-lung microbiome correlations in COPD pathogenesis

**FIGURE 8:**
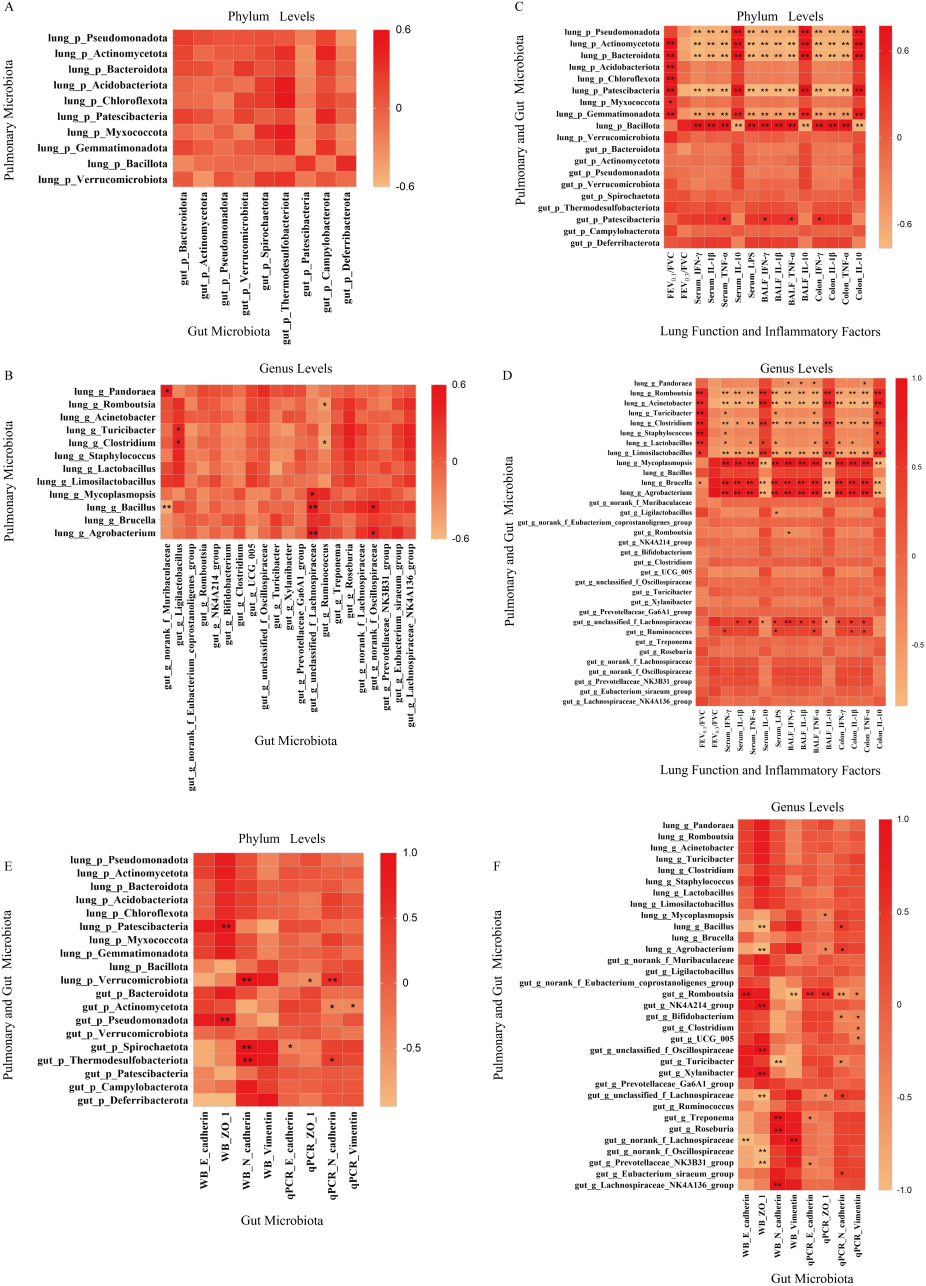
Analysis of gut-lung microbiome cross tissue associations and their correlation with lung function, inflammation and airway EMT markers. **(A,B)** Cross-tissue correlations of dominant phyla and genera between lung and gut ecosystems. **(C,D)** Correlations between microbiota (phyla, genera) and lung function (FEV0.1/FVC), inflammatory markers across serum, BALF, and colon compartments. **(E,F)** Microbiota-EMT correlations involving airway EMT biomarkers (E-cadherin, N-cadherin, ZO-1, Vimentin). Red and yellow shades denote positive and negative correlations, respectively, with color intensity reflecting correlation strength. Statistical significance: *p < 0.05, **p < 0.01.

To elucidate the microbial correlations between gut and lung in COPD pathogenesis, we analyzed cross-system correlations of dominant phyla and genera (Figures 8A,B).

Five phyla were co-expressed in both ecosystems: Pseudomonadota, Actinomycetota, Bacteroidota, Patescibacteria, and Verrucomicrobiota. Cross-tissue correlation analyses at the phylum level revealed concordant abundance trends between lung and gut ecosystems: positive correlations were observed for Pseudomonadota (*r* = 0.07, *P* > 0.05) and Bacteroidota (*r* = 0.14, *P* > 0.05), whereas negative correlations characterized Patescibacteria (*r* = −0.33, *P* > 0.05) and Verrucomicrobiota (*r* = −0.125, *P* > 0.05), though none reached statistical significance.

At the genus level, three taxa–*Romboutsia*, *Turicibacter*, and *Clostridium*–were co-expressed in both sites, with *Romboutsia* demonstrating a positive inter-system correlation (*r* = 0.07, *P* > 0.05). Critically, genus-level analyses revealed significant positive correlations between gut *Ligilactobacillus* and lung *Turicibacter* (*r* = 0.41, *P* > 0.05) or *Clostridium* (*r* = 0.41, *P* < 0.05). Additionally, gut unclassified_f_Lachnospiraceae displayed positive associations with lung *Mycoplasmopsis* (*r* = 0.44, *P* < 0.05), *Bacillus* (*r* = 0.52, *P* < 0.01), and *Agrobacterium* (*r* = 0.60, *P* < 0.01), while gut *norank_f_Oscillospiraceae* correlated positively with lung *Bacillus* (*r* = 0.50, *P* < 0.05) and *Agrobacterium* (*r* = 0.45, *P* < 0.05). Conversely, gut *Ruminococcus* showed negative correlations with lung *Romboutsia* (*r* = −0.41, *P* < 0.05) and *Clostridium* (*r* = −0.47, *P* < 0.05). Notably, gut *g_norank_f_Muribaculaceae* exhibited a positive correlation with lung *Pandoraea* (*r* = 0.44, *P* < 0.05) and a negative correlation with lung *Bacillus* (*r* = −0.52, *P* < 0.01). These findings collectively indicate compartmentalized crosstalk between lung and gut microbiota during COPD progression, with both statistically robust and trend-level interactions informing disease mechanisms.

#### 3.5.2 EA-mediated microbial shifts associate with improved lung function and reduced inflammation

Significant correlations were identified between lung and gut microbiota with lung function and inflammatory markers (Figures 8C,D).

For lung microbiota, at the phylum level, EA-upregulated taxa including Actinomycetota (*P* < 0.01), Acidobacteriota (*P* < 0.01), Chloroflexota (*P* < 0.01), Patescibacteria (*P* < 0.01), Myxococcota (*P* < 0.05), and Gemmatimonadota (*P* < 0.01) showed positive correlations with FEV0.1/FVC. EA-upregulated Pseudomonadota, Actinomycetota, Bacteroidota, Patescibacteria, and Gemmatimonadota positively correlated with IL-10 in serum, BALF, and colon, but inversely correlated with IFN-γ, IL-1β, TNF-α (serum, BALF, colon), and serum LPS (*P* < 0.01). Conversely, EA-downregulated Bacillota (*P* < 0.01) exhibited negative associations with IL-10 (serum, BALF, colon) and positive correlations with pro-inflammatory mediators across compartments (*P* < 0.01). At genus level, EA-upregulated *Romboutsia* (*P* < 0.01), *Acinetobacter* (*P* < 0.01), *Turicibacter* (*P* < 0.01), *Clostridium* (*P* < 0.01), *Staphylococcus* (*P* < 0.01), *Lactobacillus* (*P* < 0.01), and *Limosilactobacillus* (*P* < 0.05) positively correlated with FEV0.1/FVC but negatively with *Brucella* (*P* < 0.05). These genera demonstrated positive associations with IL-10 and inverse relationships with IFN-γ, IL-1β, TNF-α, and LPS (*P* < 0.01). Lung *Pandoraea* inversely correlated with BALF IFN-γ, IL-1β, TNF-α and colon TNF-α (*P* < 0.05), while *Turicibacter* showed negative correlations with serum IFN-γ, LPS and BALF TNF-α but positive with colon IL-10 (*P* < 0.05). EA-upregulated *Lactobacillus* exhibited positive correlations with IL-10 (serum, BALF, colon) and negative associations with IFN-γ, TNF-α (serum, BALF), colon IFN-γ, IL-1β, and serum LPS (*P* < 0.05). EA-downregulated *Mycoplasmopsis*, *Brucella*, and *Agrobacterium* displayed negative associations with IL-10 and positive with IFN-γ, IL-1β, TNF-α, LPS (*P* < 0.01).

For gut microbiota, EA-downregulated Patescibacteria displayed positive correlations with TNF-α (serum, BALF) and IFN-γ (BALF, colon) (*P* < 0.05). EA-upregulated *Ligilactobacillus* and *Romboutsia* inversely correlated with serum LPS (*P* < 0.05) and BALF IFN-γ (*P* < 0.05). Gut unclassified_f_Lachnospiraceae showed inverse correlations with IL-10 and positive links to IL-1β, TNF-α, LPS (serum) and IFN-γ, IL-1β, TNF-α (BALF, colon) (*P* < 0.05). Gut Ruminococcus exhibited positive associations with serum IFN-γ, LPS, BALF TNF-α, IL-1β, and colon TNF-α (*P* < 0.05). Notably, convergent regulatory patterns emerged. EA-enriched taxa with consistently associated with reduced systemic LPS and elevated IL-10, while EA-suppressed pro-inflammatory genera (lung *Mycoplasmopsis* and gut unclassified_f_Lachnospiraceae) uniformly correlated with elevated TNF-α and IFN-γ. Synchronized functional links were observed for lung function improvement, correlating with both lung Actinomycetota enrichment and gut Patescibacteria depletion. These findings collectively demonstrate compartmentalized microbiota-functional interactions linking lung and gut microbial shifts to lung function and inflammatory responses across multiple compartments in COPD.

#### 3.5.3 EA-induced microbial changes inversely correlate with airway EMT progression

As shown in Figures 8E,F, significant correlations were observed between lung and gut microbiota and airway EMT biomarkers.

For lung microbiota, at the phylum level, EA-upregulated Patescibacteria positively correlated with ZO-1 protein expression (*P* < 0.01) while EA-downregulated Verrucomicrobiota showed positive associations with N-cadherin protein (*P* < 0.01) and mRNA (*P* < 0.01) plus inverse correlation with ZO-1 mRNA (*P* < 0.05). Genus-level examination demonstrated EA-downregulated *Mycoplasmopsis* and *Agrobacterium* inversely correlated with ZO-1 mRNA (both *P* < 0.05), while *Agrobacterium* positively associating with N-cadherin mRNA (*P* < 0.05) with *Bacillus* reduction linking to suppressed ZO-1 protein (*P* < 0.01) and elevated N-cadherin mRNA (*P* < 0.05).

For gut microbiota, phylum-level analysis showed EA-upregulated Pseudomonadota exhibited positive ZO-1 protein correlation (*P* < 0.01) whereas EA-downregulated Spirochaetota positively correlated with N-cadherin protein (*P* < 0.01) but negatively with E-cadherin mRNA (*P* < 0.05) and EA-downregulated Thermodesulfobacteriota displayed positive N-cadherin protein (*P* < 0.01) and mRNA correlations (*P* < 0.05). At genus level EA-enhanced *Romboutsia* positively correlated with E-cadherin protein (*P* < 0.01) while negatively associating with Vimentin protein (*P* < 0.01) and mRNA (*P* < 0.05) plus positive ZO-1 mRNA correlation (*P* < 0.01) whereas EA-downregulated unclassified_f_Lachnospiraceae inversely correlated with E-cadherin protein (*P* < 0.01) and ZO-1 protein (*P* < 0.01). Notably EA-upregulated *Turicibacter* negatively correlated with N-cadherin protein (*P* < 0.01).

These microbial shifts in lung and gut compartments collectively demonstrate that electroacupuncture attenuates airway EMT progression through convergent mechanisms. In both lung and gut ecosystems, EA-enriched taxa (lung Patescibacteria, gut *Romboutsia*) consistently associate with epithelial marker preservation, while EA-suppressed taxa (lung Verrucomicrobiota, gut Spirochaetota) uniformly correlate with mesenchymal marker reduction, revealing EA’s integrated regulation of gut-lung axis homeostasis via modulation of microbiota dysbiosis and EMT pathways.

## 4 Discussion

This study explores whether EA-associated modulation of gut and lung microbiota is linked to reduced systemic inflammation and EMT inhibition, and thereby may contribute to attenuation of airway remodeling in COPD. Across outcomes, EA (but not sham acupuncture) was consistently associated with improved lung function and airway remodeling indices, accompanied by coordinated shifts in key taxa, lower serum LPS, and partial normalization of EMT markers. This suggests a link between EA’s modulation of the microbial microenvironment and the attenuation of EMT, although causality remains to be confirmed.

Notably, the improvement in core lung function indices reflects the alleviation of small airway obstruction and restoration of lung tissue elasticity (Brake et al., 2023; Xu J. et al., 2024). While the degree of lung function improvement achieved by EA in this study was comparable to that reported in previous acupuncture studies on COPD, their underlying mechanisms are fundamentally distinct (Zhang et al., 2018). Previous acupuncture studies have primarily focused on direct anti-inflammatory effects or modulation of respiratory muscle function (Liu Q. et al., 2021). In contrast, EA modulates the functional state of the gut microbiota, enhancing intestinal motility and microbial metabolic activity to promote beneficial metabolites like SCFAs, thereby indirectly optimizing the mechanical properties of lung tissue (Zhang et al., 2022; Wang L. et al., 2023; Wang et al., 2025). The observed reduction in circulating endotoxin levels was closely linked to the enrichment of beneficial gut bacteria, further supporting the “gut microbiota dysbiosis-endotoxin translocation-lung inflammation” regulatory theory (Lai et al., 2022; Wang L. et al., 2023; Song X. et al., 2024). A critical finding is that the reversal of EMT–a direct driver of airway fibrosis (Eapen and Sohal, 2020; Ding et al., 2024)–was highly correlated with the decline in endotoxin. This suggests that microbial metabolites (e.g., butyrate) may interfere with pro-fibrotic signaling pathways (e.g., TGF-β signaling) via epigenetic regulation, synergizing with endotoxin reduction to suppress the EMT process (Zhao et al., 2023; Wei J. et al., 2024; Sangamesh et al., 2025). Our results accord with prior reports that acupuncture can beneficially modulate inflammation and microbiota in COPD. A multicenter trial reported improved lung function and symptoms in COPD patients receiving acupuncture, alongside hints of reduced systemic inflammation (Liu et al., 2025). Similarly, lectroacupuncture enhanced beneficial gut bacteria and SCFA levels in a perinatal lung injury model, leading to improved pulmonary outcomes. Our findings corroborate these studies, while providing new mechanistic insight into the microbiota-EMT link in COPD (Xie B. et al., 2024). However, unlike previous works, our study uniquely connects these microbial changes to the reversal of airway EMT, highlighting a novel therapeutic intersection between microbiota and tissue remodeling.

The profound value of EA regulation may lies in its ability to induce a coordinated functional synergy between the gut and lung microbiota (Bowerman et al., 2020; Wang L. et al., 2023). Prior evidence suggests that EA at ST36 can enrich beneficial intestinal taxa and strengthen barrier-related functions, which may reduce endotoxin (LPS) release/translocation and thereby attenuate downstream inflammatory signaling (Chang et al., 2024; Wang J. et al., 2024; Wang Y. et al., 2024), while EA at BL13 may reshape the pulmonary microbial and immune microenvironment toward an anti-inflammatory/anti-fibrotic profile (Jacobson et al., 2018; Yu et al., 2024). SCFAs (e.g., butyrate) have also been reported to modulate epithelial integrity and inflammation, and our PICRUSt2 results (e.g., K06147) suggest shifts in lipid-metabolic functional potential that could be related to SCFA-associated functions (Mrkvicova et al., 2019; Esteves et al., 2021; Ikeda et al., 2022; Zhou et al., 2025). Taken together, a plausible hypothesis is that reduced LPS-related inflammatory pressure together with enhanced SCFA-/butyrate-related functional potential may converge on dampening pro-fibrotic signaling (e.g., TGF-β-related pathways) and thereby suppress EMT-like remodeling (Li et al., 2020; Ducret and Grangeasse, 2021; Kawai et al., 2024). Furthermore, EA intervention concurrently promoted the proliferation of Bacteroidota and *Romboutsia* phyla in both gut and lung, albeit through distinct mechanisms. The increase in Bacteroidota was primarily associated with enhanced SCFA synthesis (e.g., propionate, butyrate), which mitigated systemic inflammation by modulating the AMPK/NF-κB/NLRP3 signaling axis (Wang Z. et al., 2023). In contrast, the expansion of *Romboutsia* suppressed airway fibrosis by balancing Treg/Th17 immune responses (Song et al., 2022; Sun et al., 2024). Notably, EA demonstrated organ-specific regulation tightly linked to COPD pathology. Suppression of Verrucomicrobiota in the lungs may alleviate MMP-9-mediated basement membrane degradation (Du et al., 2024), while enrichment of the same phylum in the gut, potentially via *Akkermansia muciniphila*, could strengthen the mucus barrier function (Ansaldo et al., 2019; Wu et al., 2023). Similarly, EA’s modulation of Patescibacteria exhibited organ-specificity: its depletion in the gut might reduce COPD susceptibility (Liu Y. et al., 2023), whereas its enrichment in the lungs could promote immune tolerance via tryptophan metabolic reprogramming (Xie B. et al., 2024). Crucially, this entire synergistic effect was completely absent in the sham acupuncture group, confirming its dependence on the specific acupoint stimulation by EA. We note that the sham-treated group exhibited only minor, non-specific changes, without significant improvement in lung pathology. This suggests that any small effects of sham treatment likely stem from non-specific factors, and that effective therapy requires specific acupoint stimulation by EA.

In this study, we observed a paradoxical elevation of lung microbiota α-diversity in the COPD model, which contrasts with the classical paradigm associating dysbiosis with reduced diversity. We propose that this represents a state of functional depletion disguised by high diversity, arising from competitive expansion of both pathogens and commensals within the mucus-hypersecretory niche induced by cigarette smoke exposure, ultimately leading to functional dissipation (Hart et al., 2014; Du et al., 2022). Crucially, the breakthrough significance of EA lies in its ability to counteract this by enriching functionally important bacteria (e.g., butyrate producers), thereby driving the microbiota away from quantitative expansion toward a restructuring of functional homeostasis (Yan et al., 2023; Li S. et al., 2024). Our findings align with recent evidence that acupuncture can modulate the gut-lung axis to restore microbial balance in respiratory diseases. This provides mechanistic insights into acupuncture in modulating microecology. Leveraging this functional synergy, EA demonstrates significant translational potential for COPD management. Unlike bronchodilators, which offer only limited symptomatic relief, or corticosteroids that often exacerbate microbial dysbiosis, EA achieves bidirectional regulation via the gut-lung axis (Dang and Marsland, 2019; Song X. -L. et al., 2024; Song Z. et al., 2024). It simultaneously optimizes microbiota homeostasis and inhibits the core EMT process. Consequently, circulating endotoxin levels combined with the abundance of specific gut bacterial taxa may serve as non-invasive biomarkers for early COPD diagnosis. Furthermore, combining EA with probiotics targeted at enhancing butyrate production holds promise for synergistic improvement of mucosal immunity and metabolic function, potentially elevating COPD therapeutic efficacy.

It is important to acknowledge several limitations of this study. The current animal model cannot fully capture the heterogeneity of human COPD, particularly regarding genetic variability and nuances in smoking history. Although our data support a gut–lung axis–related association (microbiota shifts accompanied by changes in serum endotoxin and EMT-related markers), intestinal barrier integrity and key mechanistic steps were not directly interrogated. Therefore, the proposed microbiota-endotoxemia-EMT framework should be interpreted as associative rather than causal. Establishing causality will require microbiota-manipulation approaches (e.g., antibiotics, fecal microbiota transplantation, or germ-free models), ideally integrated with targeted metabolite measurements (including SCFAs) and pathway-level readouts. In addition, PICRUSt2-based functional predictions and literature-informed signaling hypotheses (e.g., LPS–TLR4/NF-κB and TGF-β/Smad) were used to contextualize our findings but were not validated experimentally in this study. Finally, the relatively small sample size (*n* = 6 per group) may limit statistical power and generalizability, future work will incorporate larger cohorts and/or prospective power calculations to confirm and extend these observations.

## 5 Conclusion

In summary, our study demonstrates that electroacupuncture significantly ameliorates COPD-related airway remodeling by modulating the gut–lung microbiota axis to reduce systemic inflammation and inhibit airway EMT. These findings suggest that targeting microbiota-driven EMT processes could be a promising therapeutic strategy for COPD. Our work provides proof-of-concept that a non-pharmacological intervention like EA can beneficially reshape host-microbiome dynamics and thereby attenuate chronic lung pathology.

## Data Availability

The original contributions presented in the study are publicly available. This data can be found in the NCBI SRA repository under accession number PRJNA1426206.

## Footnotes

1
www.biocloud.net
