# Supplementary figures and images for: Electroacupuncture modulates gut-lung microbiota and lung EMT to attenuate airway remodeling in COPD
Source: Front Microbiol. 2026 Apr 1;17:1747151. doi: 10.3389/fmicb.2026.1747151 (PMC13079601; doi:10.3389/fmicb.2026.1747151)

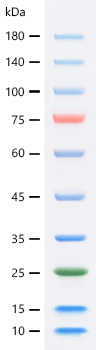

Supplement: Supplementary file 1 [file Data_Sheet_1.zip › the full uncropped Gels and Blots images/Group 1 n=3/ColorMixed Protein Marker 180 (10-180 kDa).jpg]

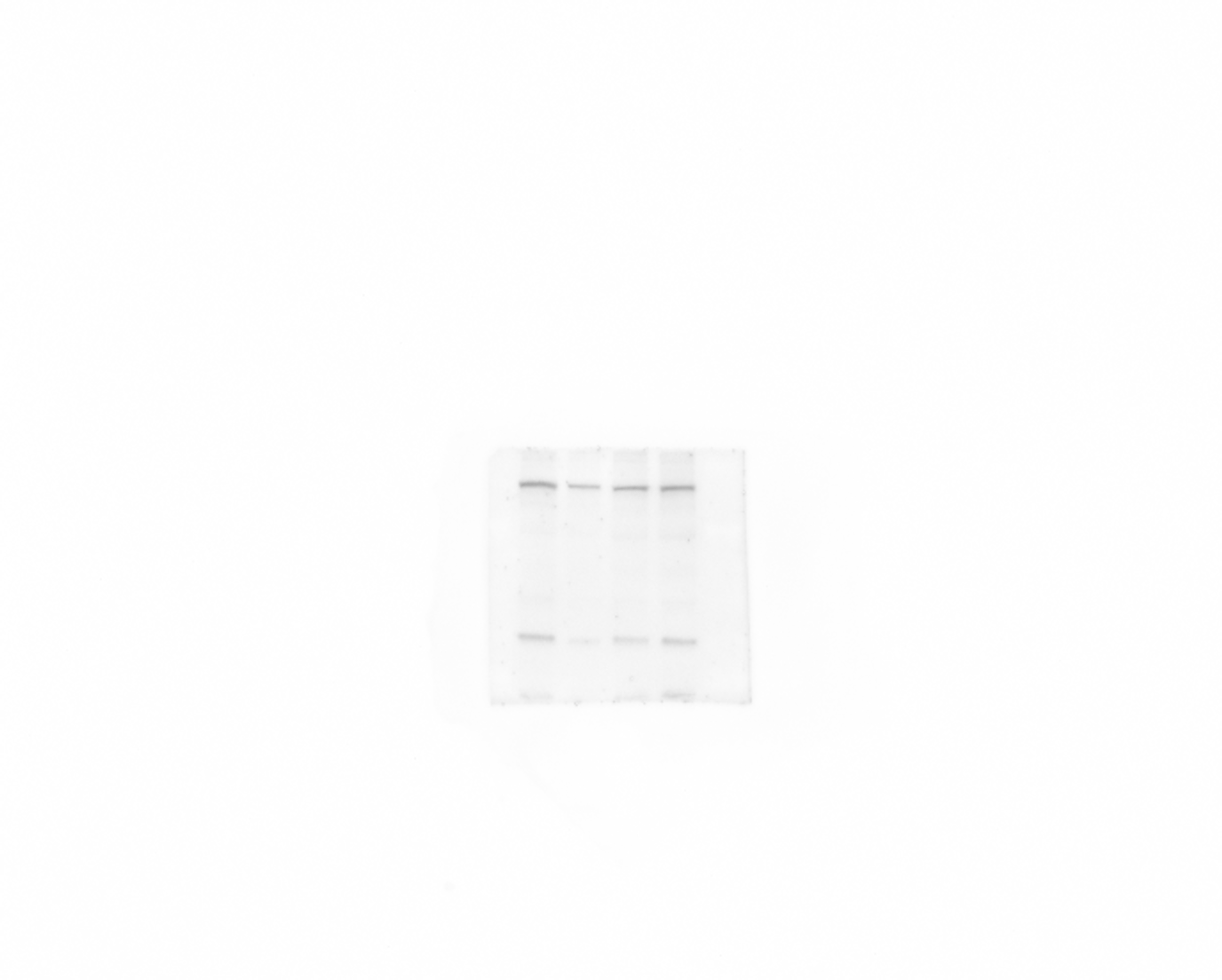

Supplement: Supplementary file 1 [file Data_Sheet_1.zip › the full uncropped Gels and Blots images/Group 1 n=3/E-cadherin/1-2s.Tif]

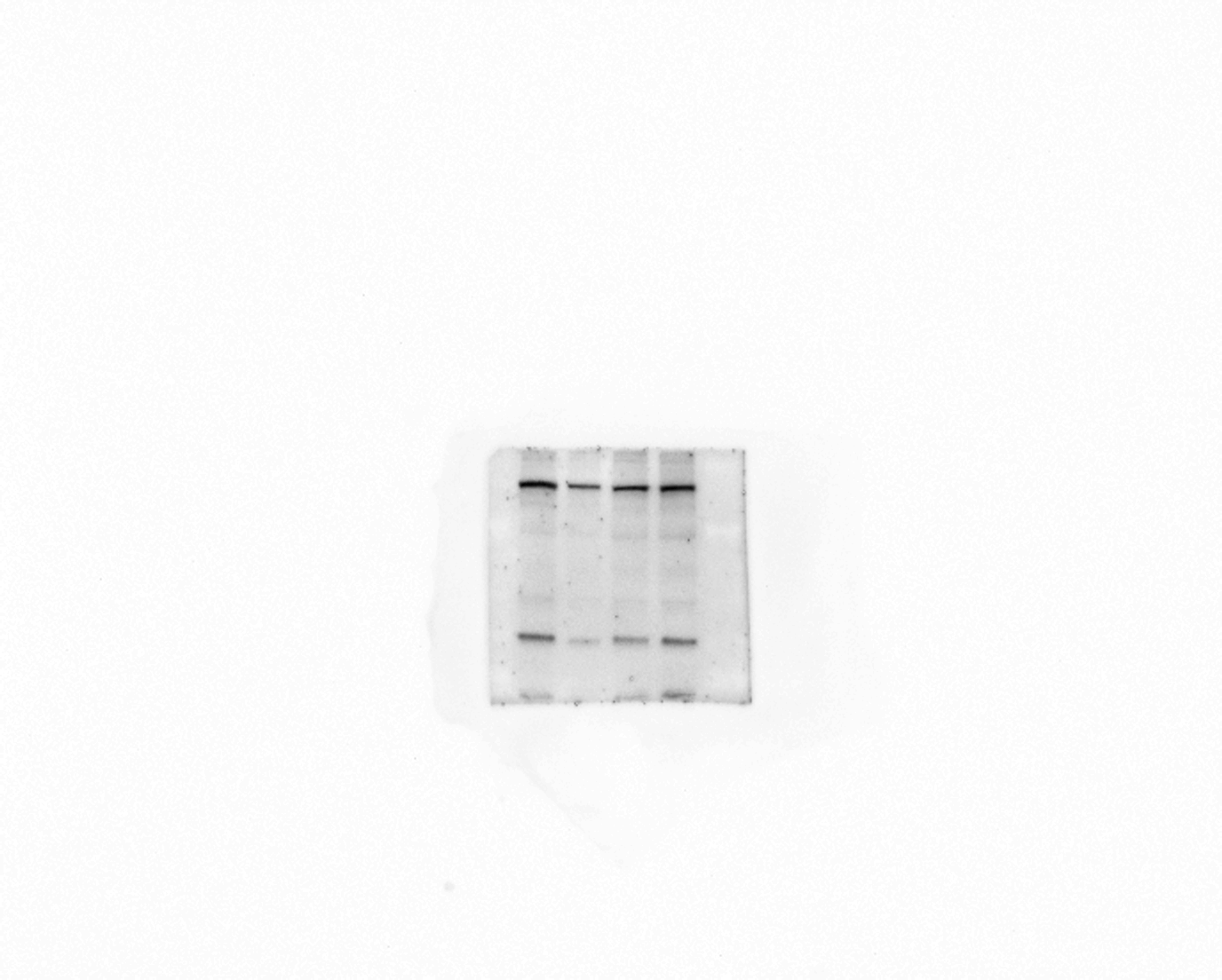

Supplement: Supplementary file 1 [file Data_Sheet_1.zip › the full uncropped Gels and Blots images/Group 1 n=3/E-cadherin/1-6s.Tif]

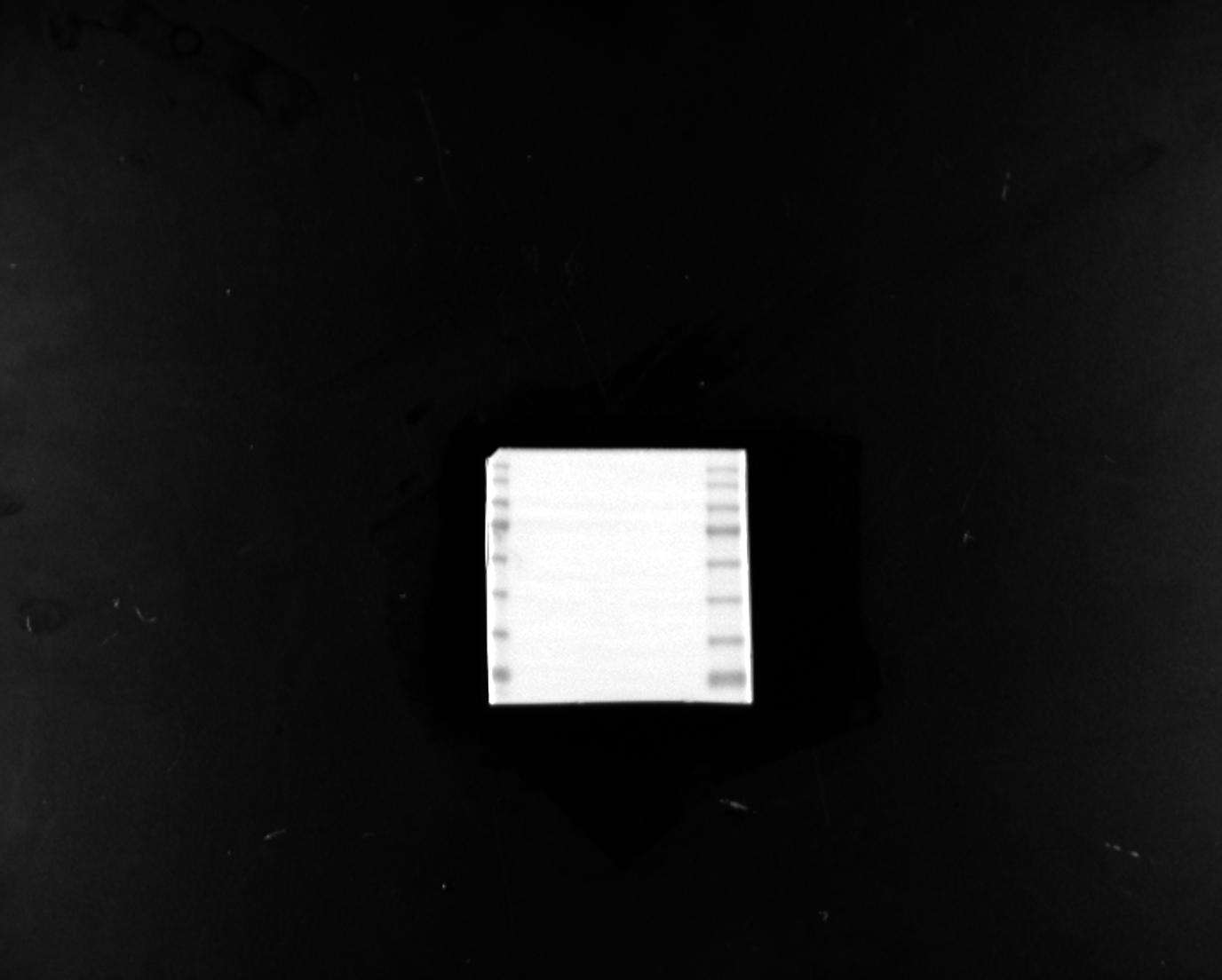

Supplement: Supplementary file 1 [file Data_Sheet_1.zip › the full uncropped Gels and Blots images/Group 1 n=3/E-cadherin/1-t.Tif]

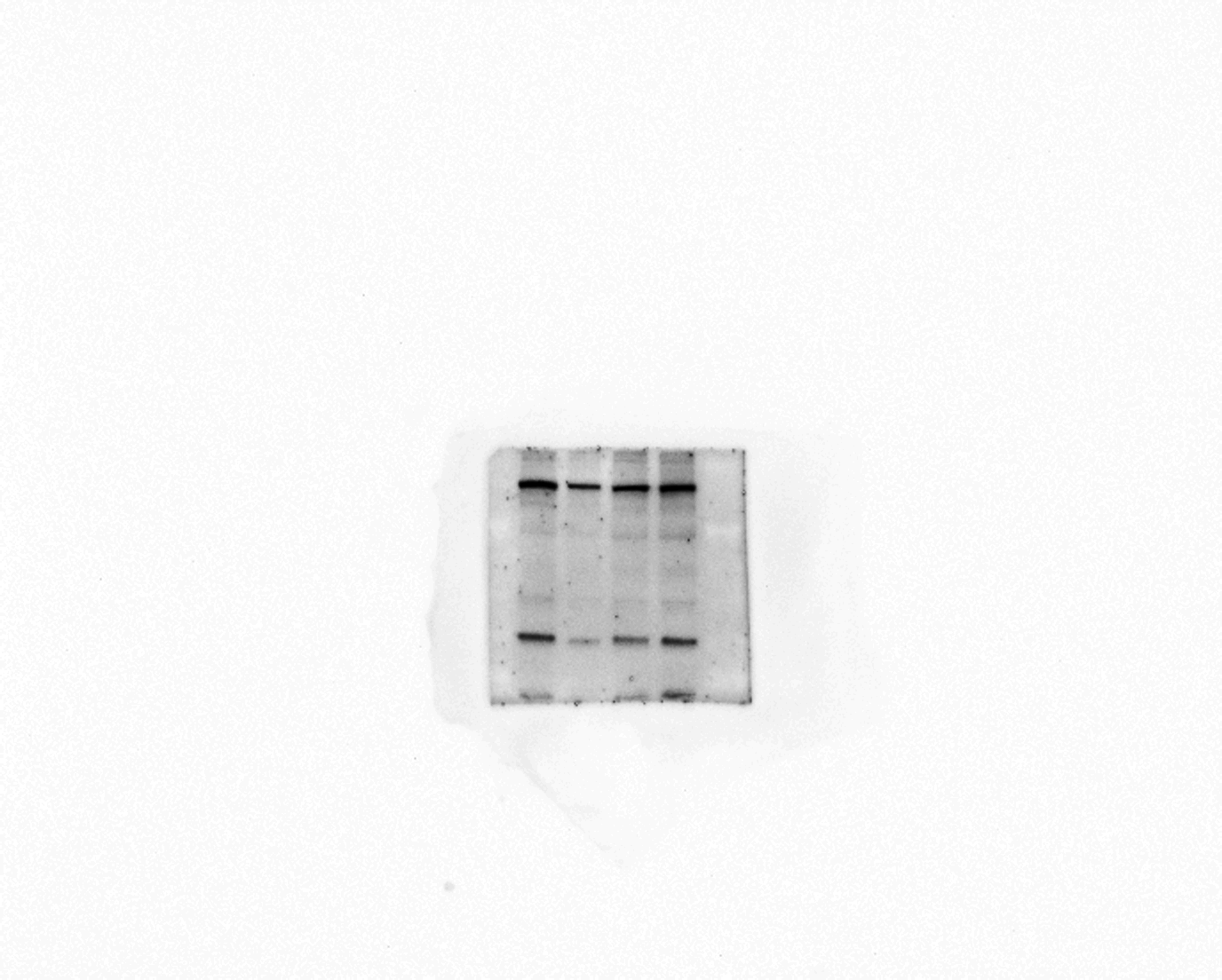

Supplement: Supplementary file 1 [file Data_Sheet_1.zip › the full uncropped Gels and Blots images/Group 1 n=3/E-cadherin/1.Tif]

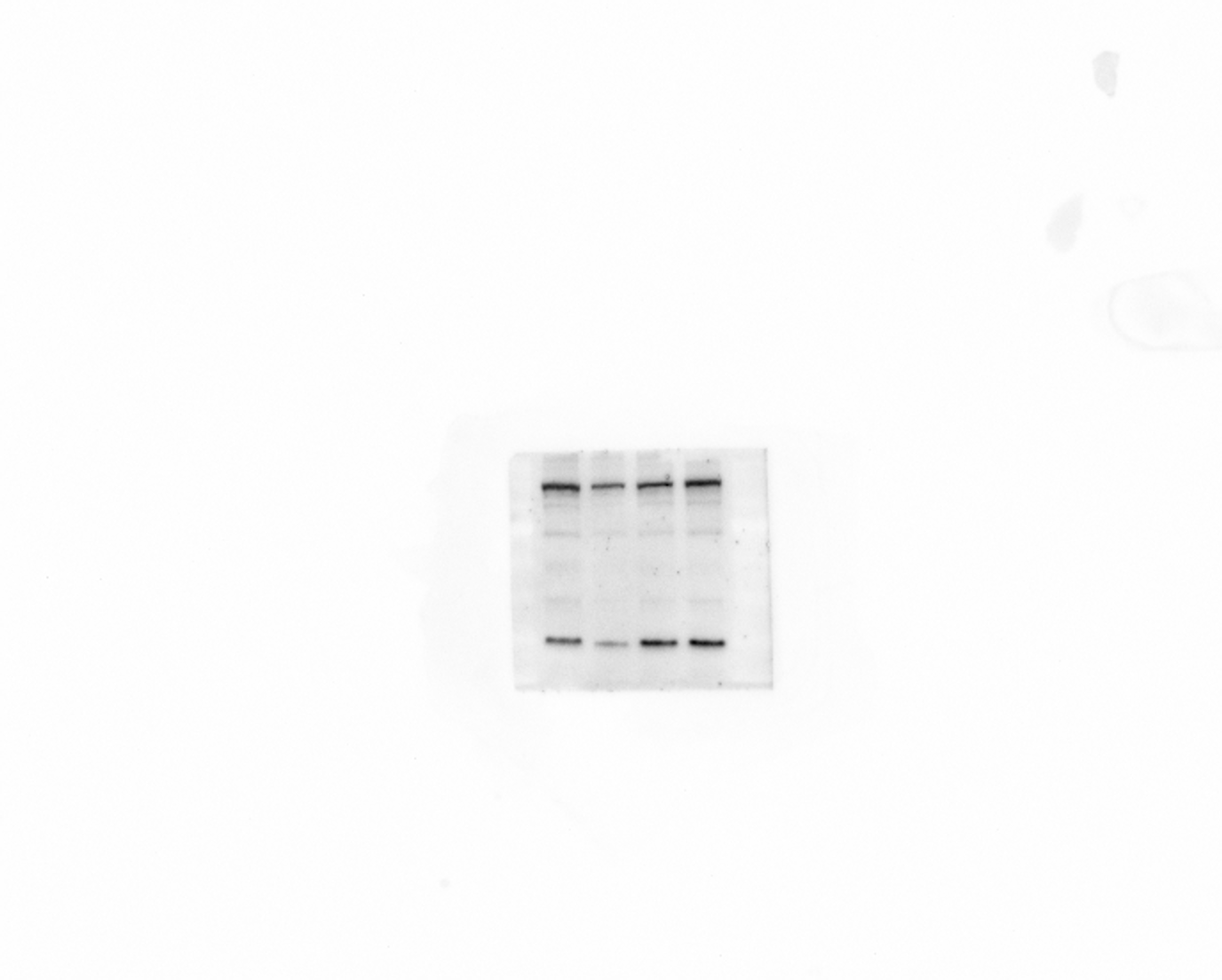

Supplement: Supplementary file 1 [file Data_Sheet_1.zip › the full uncropped Gels and Blots images/Group 1 n=3/E-cadherin/2-3s.Tif]

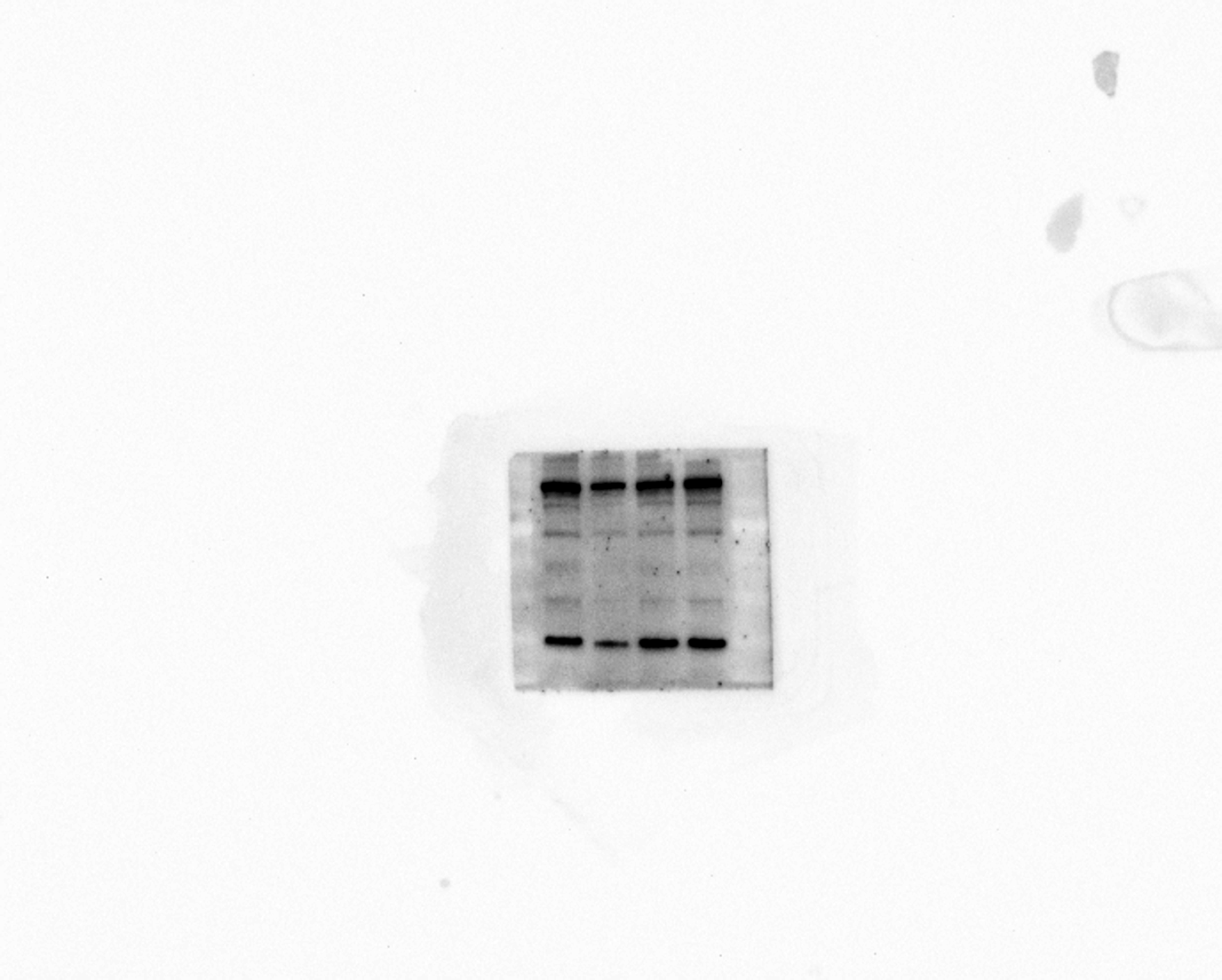

Supplement: Supplementary file 1 [file Data_Sheet_1.zip › the full uncropped Gels and Blots images/Group 1 n=3/E-cadherin/2-8s.tif]

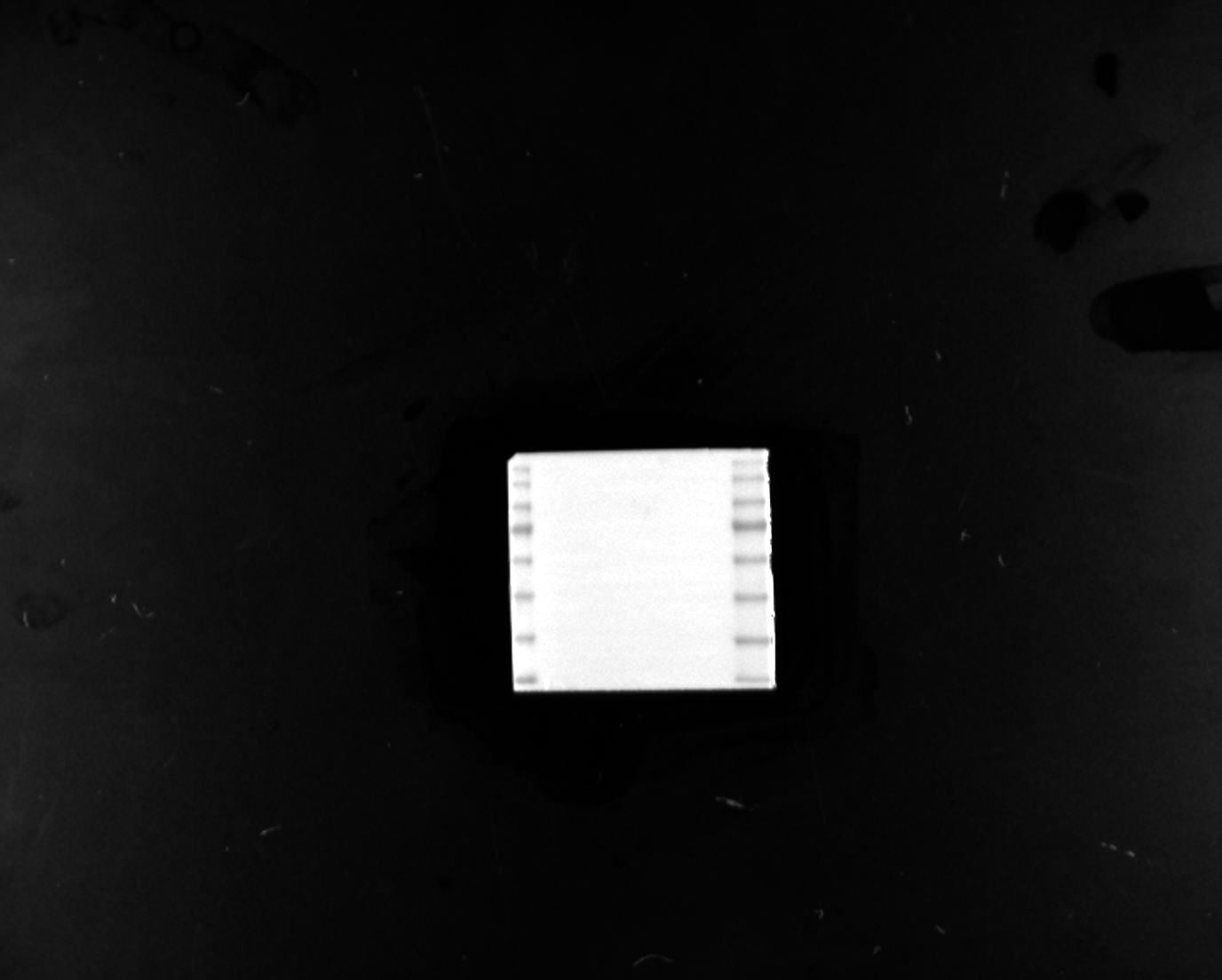

Supplement: Supplementary file 1 [file Data_Sheet_1.zip › the full uncropped Gels and Blots images/Group 1 n=3/E-cadherin/2-t.Tif]

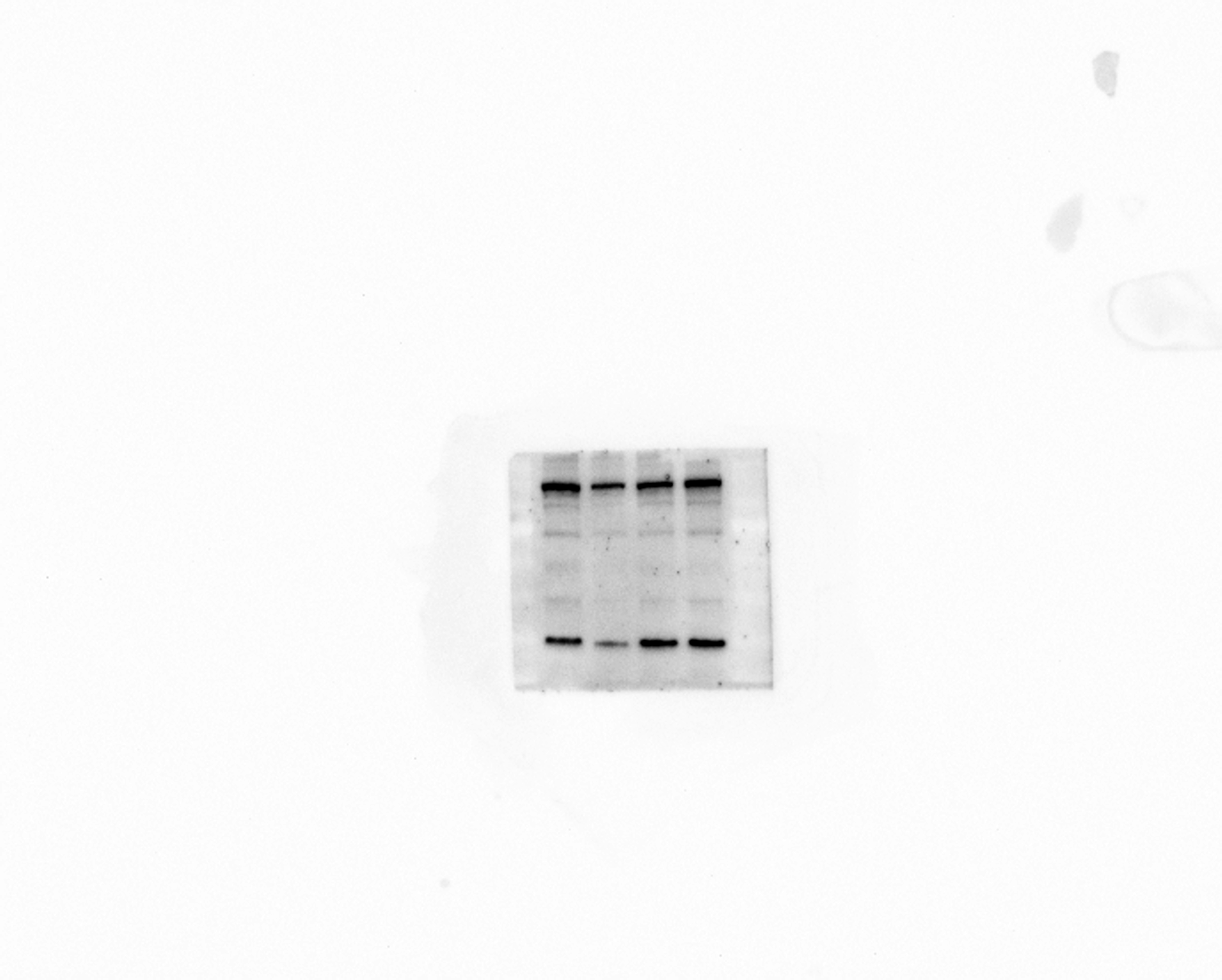

Supplement: Supplementary file 1 [file Data_Sheet_1.zip › the full uncropped Gels and Blots images/Group 1 n=3/E-cadherin/2.Tif]

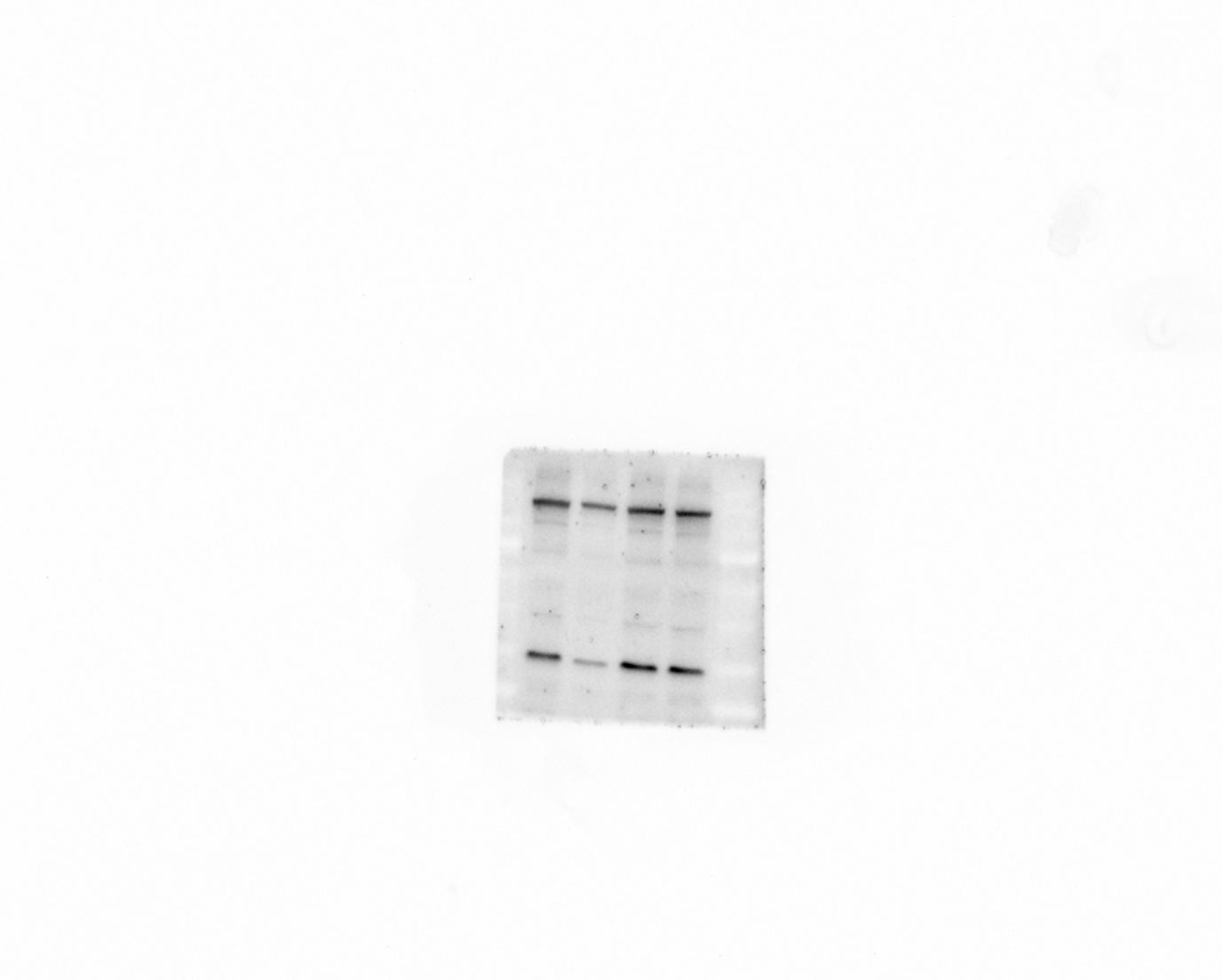

Supplement: Supplementary file 1 [file Data_Sheet_1.zip › the full uncropped Gels and Blots images/Group 1 n=3/E-cadherin/3-4s.Tif]

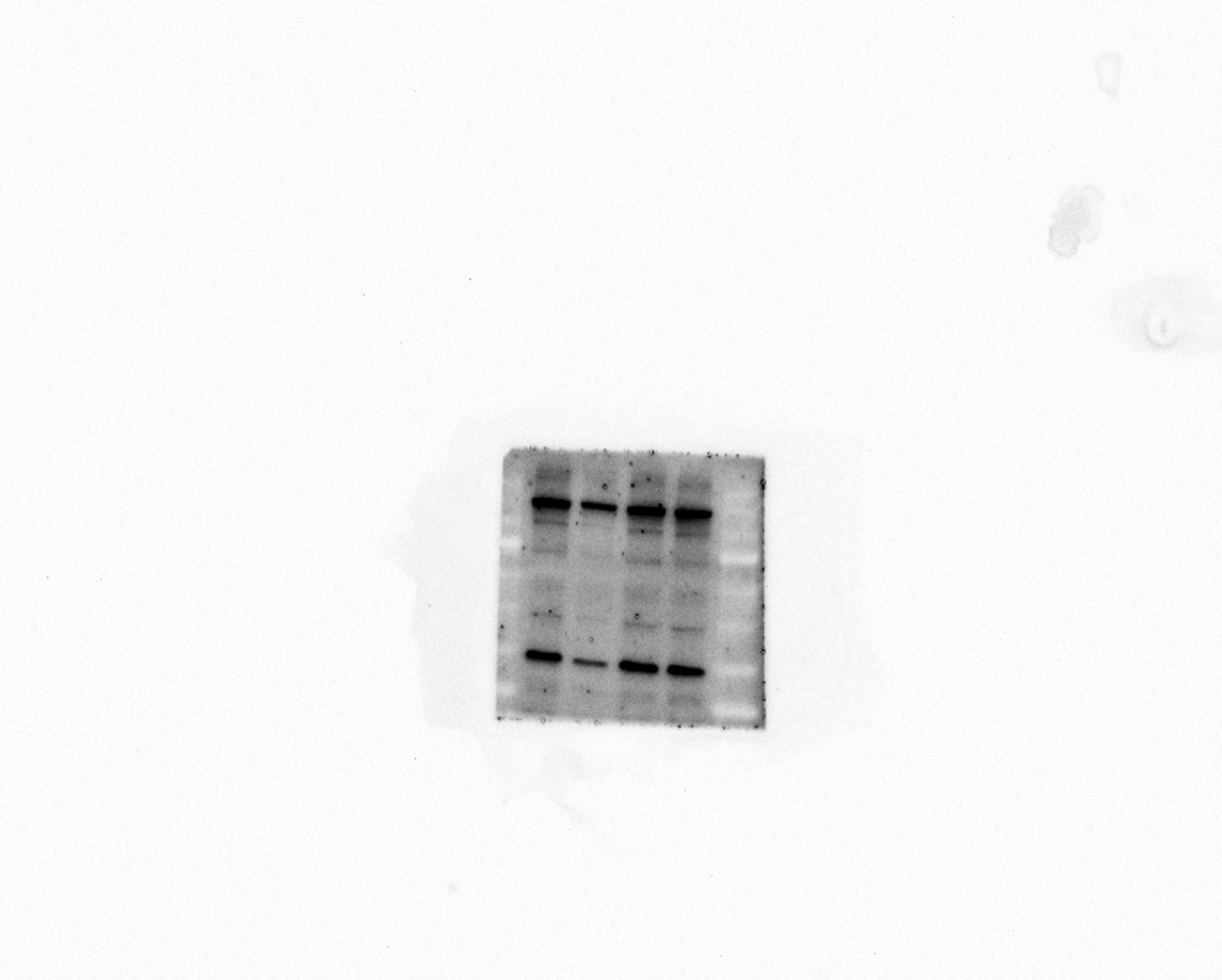

Supplement: Supplementary file 1 [file Data_Sheet_1.zip › the full uncropped Gels and Blots images/Group 1 n=3/E-cadherin/3-8s.tif]

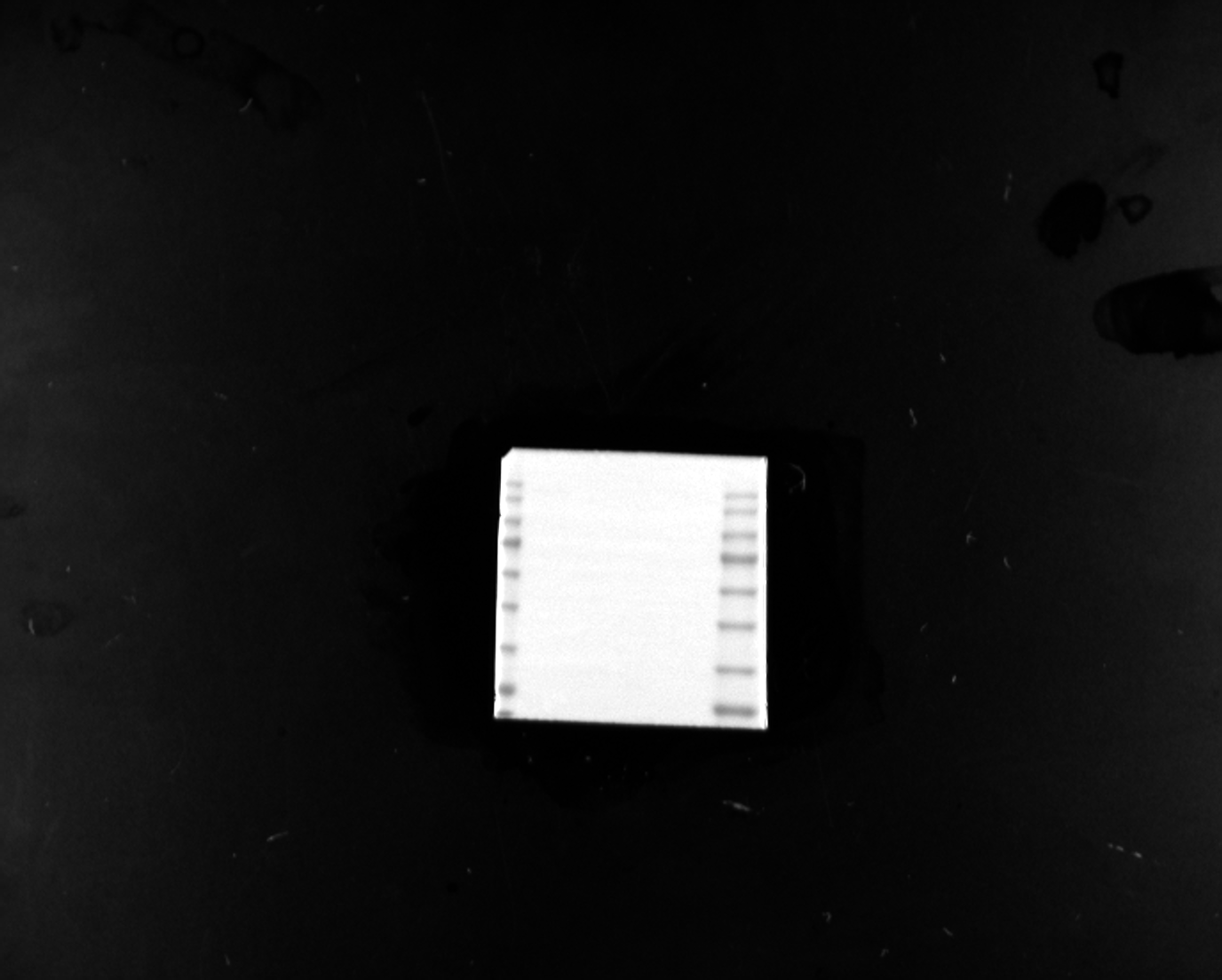

Supplement: Supplementary file 1 [file Data_Sheet_1.zip › the full uncropped Gels and Blots images/Group 1 n=3/E-cadherin/3-t.Tif]

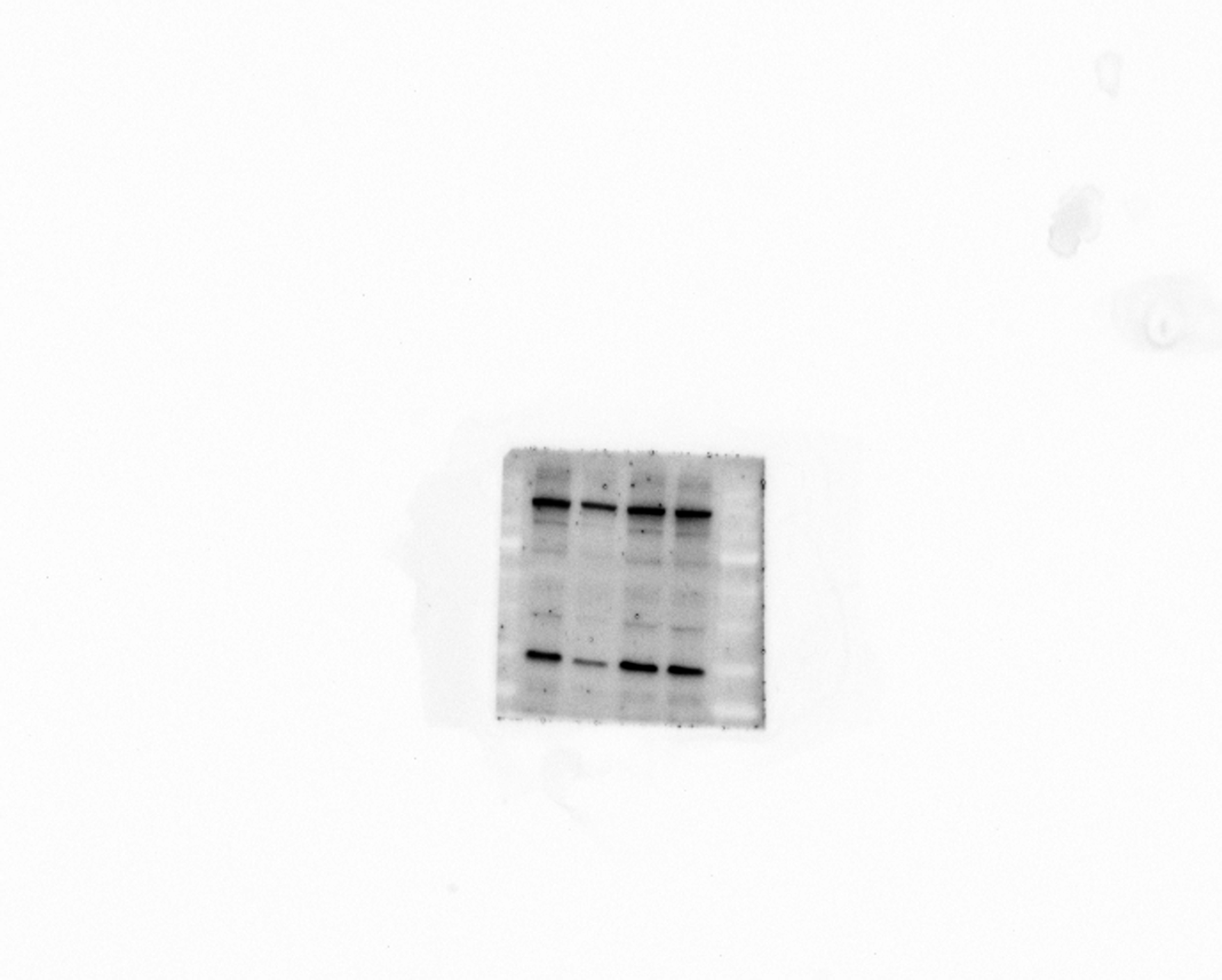

Supplement: Supplementary file 1 [file Data_Sheet_1.zip › the full uncropped Gels and Blots images/Group 1 n=3/E-cadherin/3.Tif]

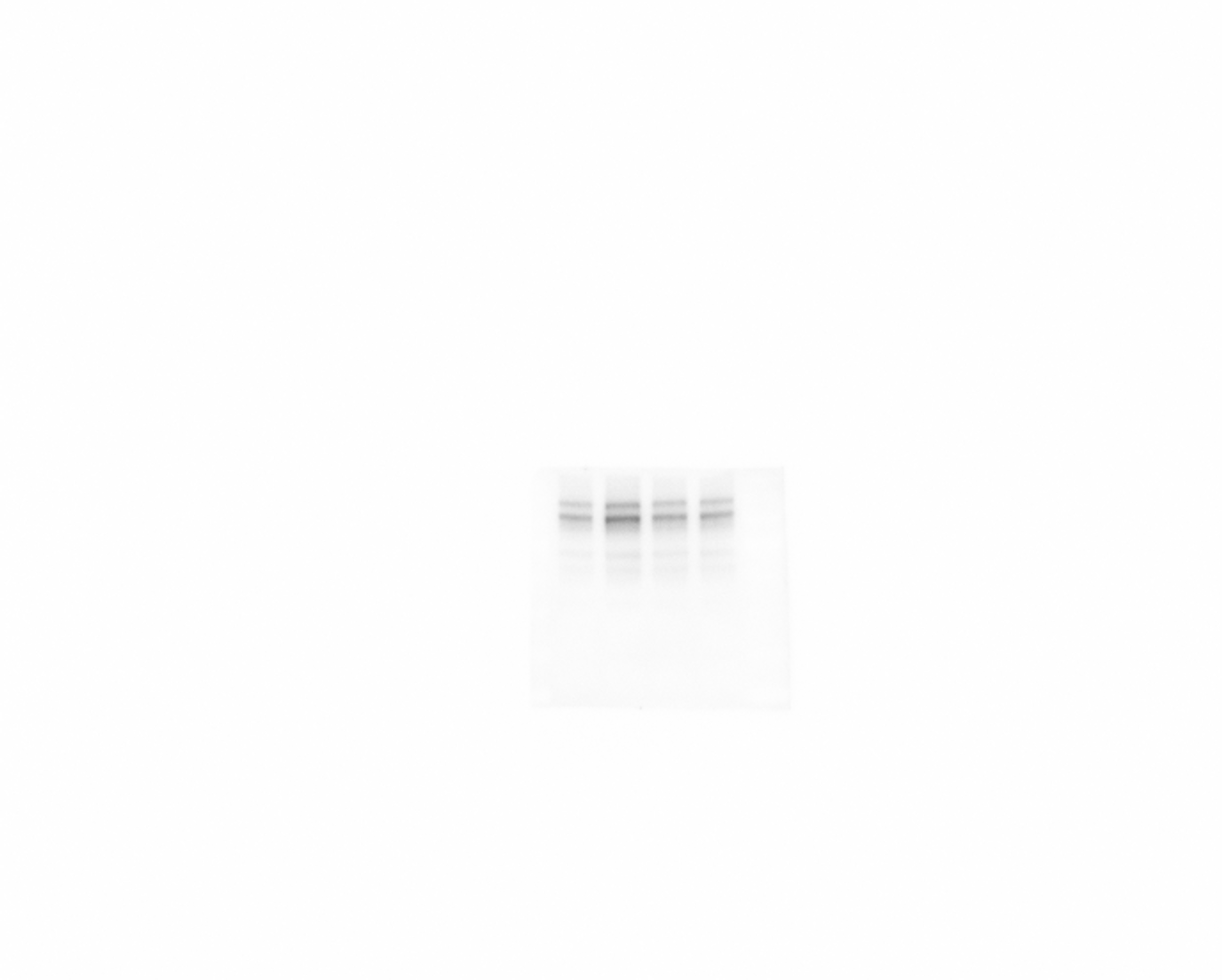

Supplement: Supplementary file 1 [file Data_Sheet_1.zip › the full uncropped Gels and Blots images/Group 1 n=3/N-cadherin/1-3s.Tif]

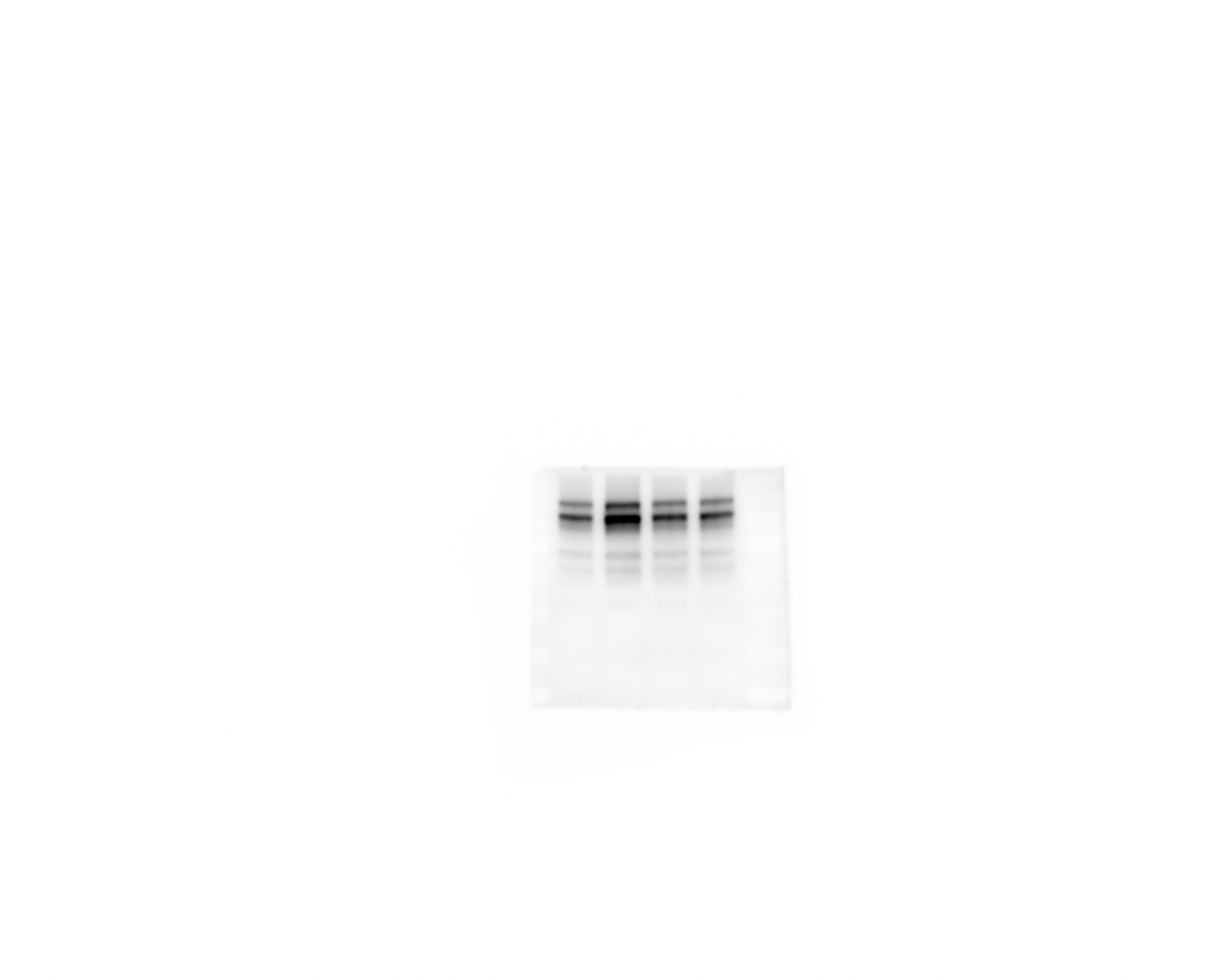

Supplement: Supplementary file 1 [file Data_Sheet_1.zip › the full uncropped Gels and Blots images/Group 1 n=3/N-cadherin/1-5s.Tif]

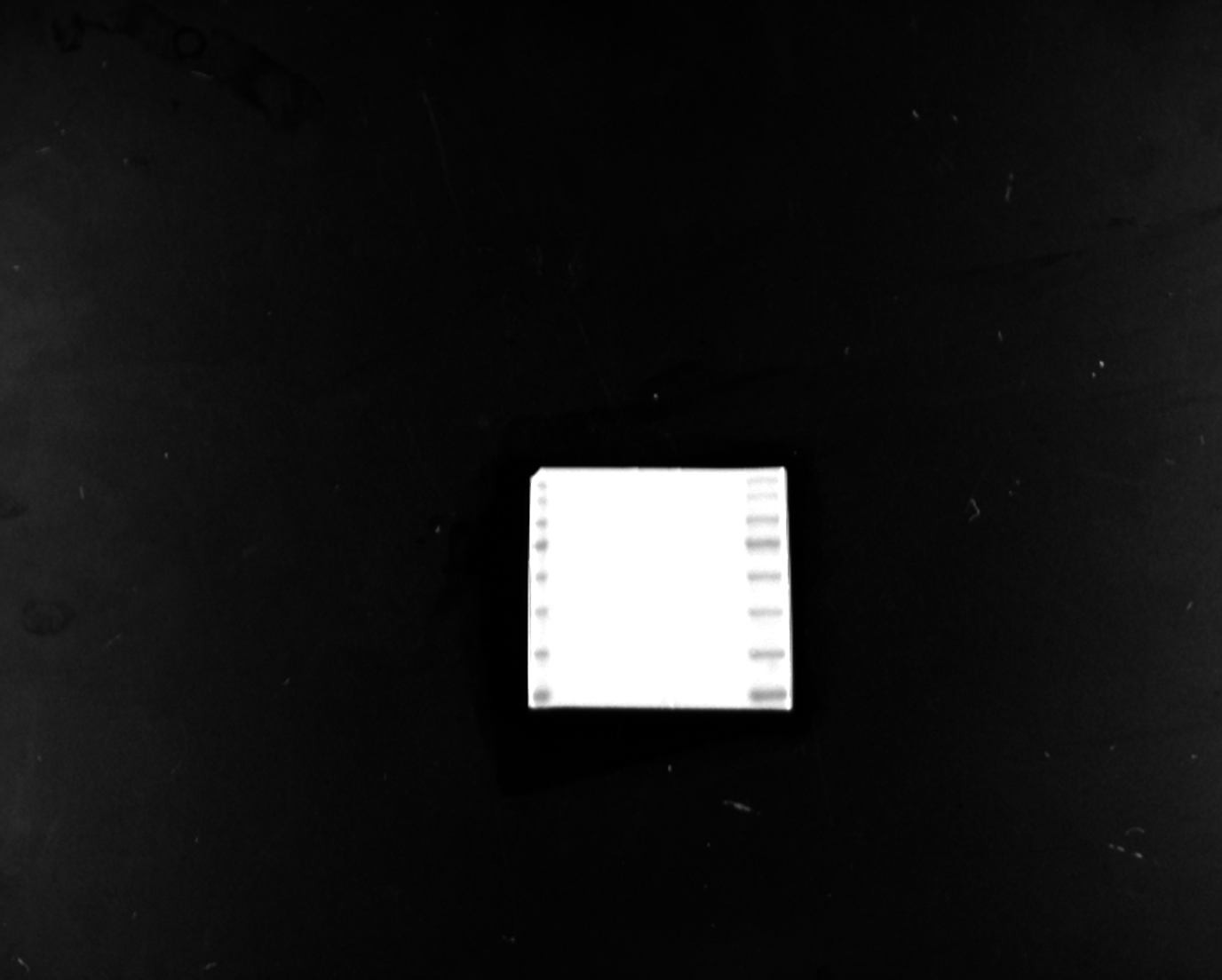

Supplement: Supplementary file 1 [file Data_Sheet_1.zip › the full uncropped Gels and Blots images/Group 1 n=3/N-cadherin/1-t.Tif]

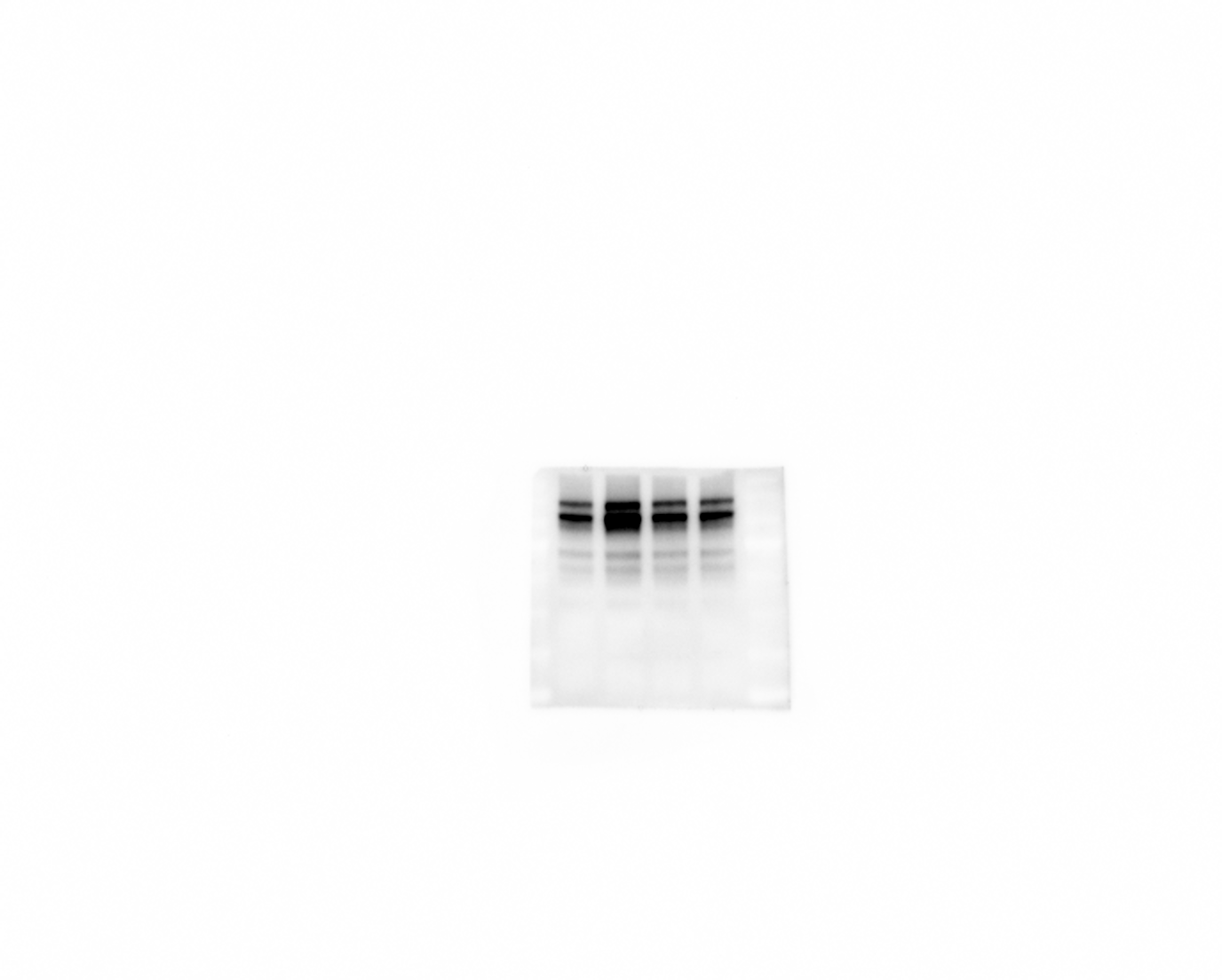

Supplement: Supplementary file 1 [file Data_Sheet_1.zip › the full uncropped Gels and Blots images/Group 1 n=3/N-cadherin/1.Tif]

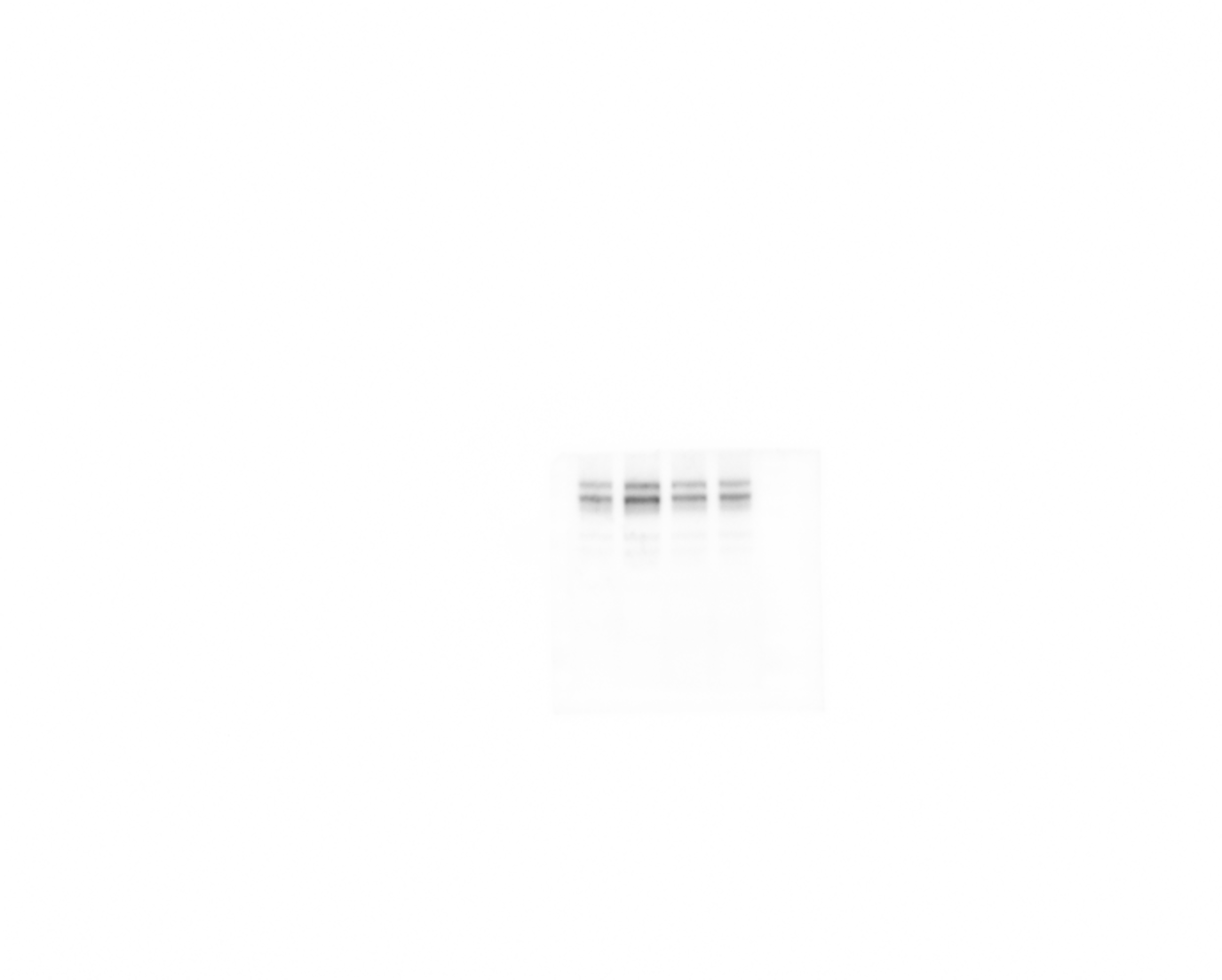

Supplement: Supplementary file 1 [file Data_Sheet_1.zip › the full uncropped Gels and Blots images/Group 1 n=3/N-cadherin/2-3s.Tif]

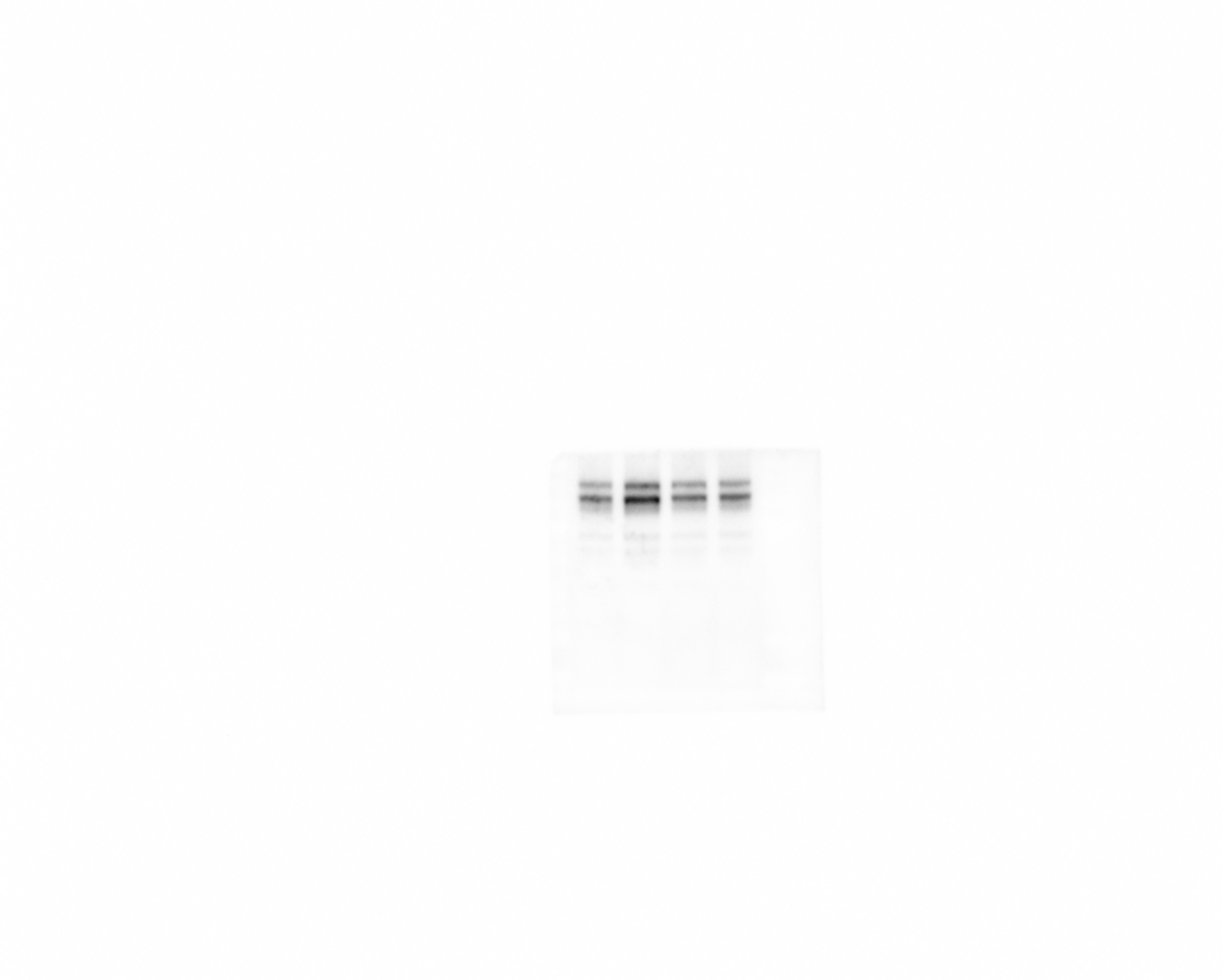

Supplement: Supplementary file 1 [file Data_Sheet_1.zip › the full uncropped Gels and Blots images/Group 1 n=3/N-cadherin/2-5s.Tif]

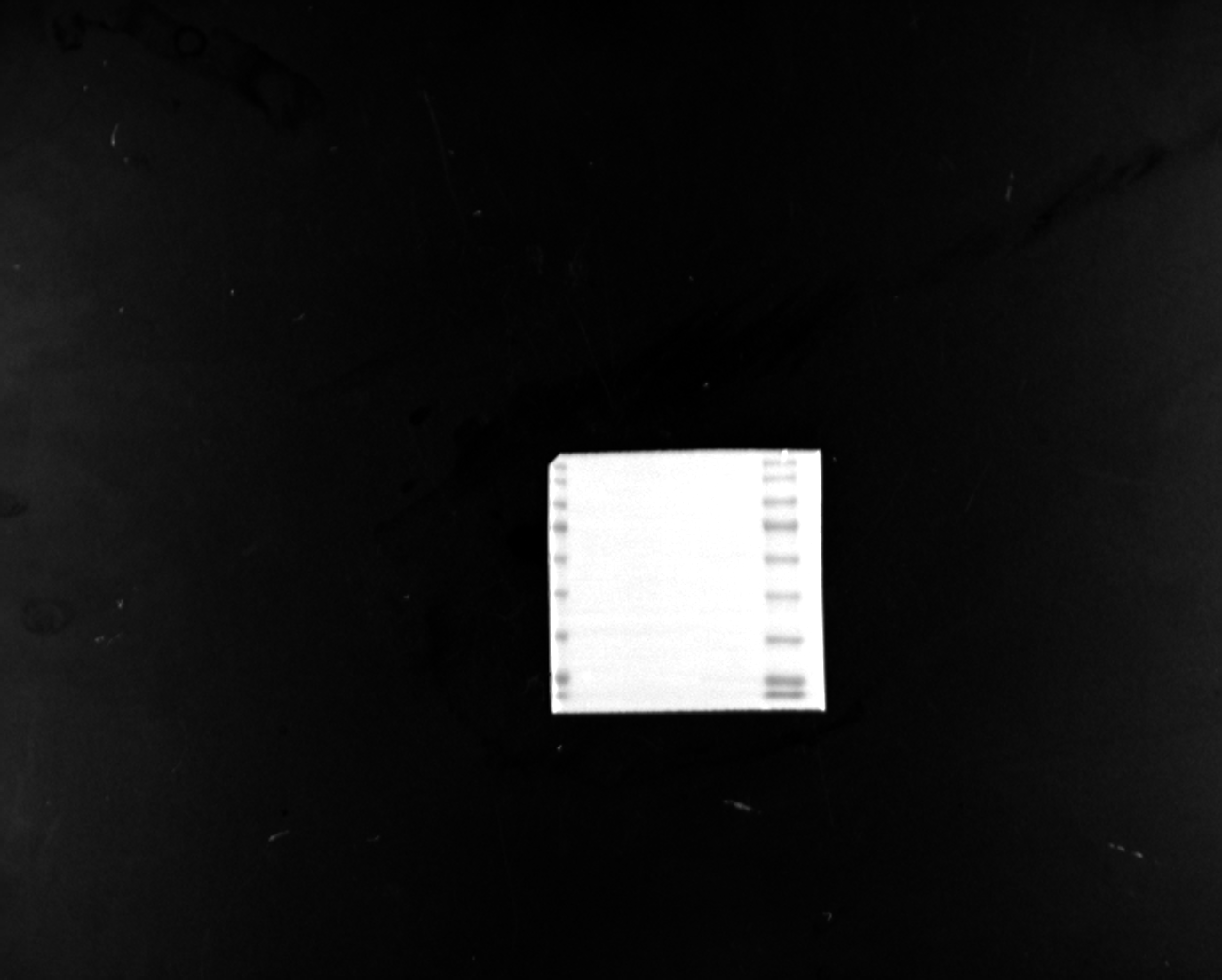

Supplement: Supplementary file 1 [file Data_Sheet_1.zip › the full uncropped Gels and Blots images/Group 1 n=3/N-cadherin/2-t.Tif]

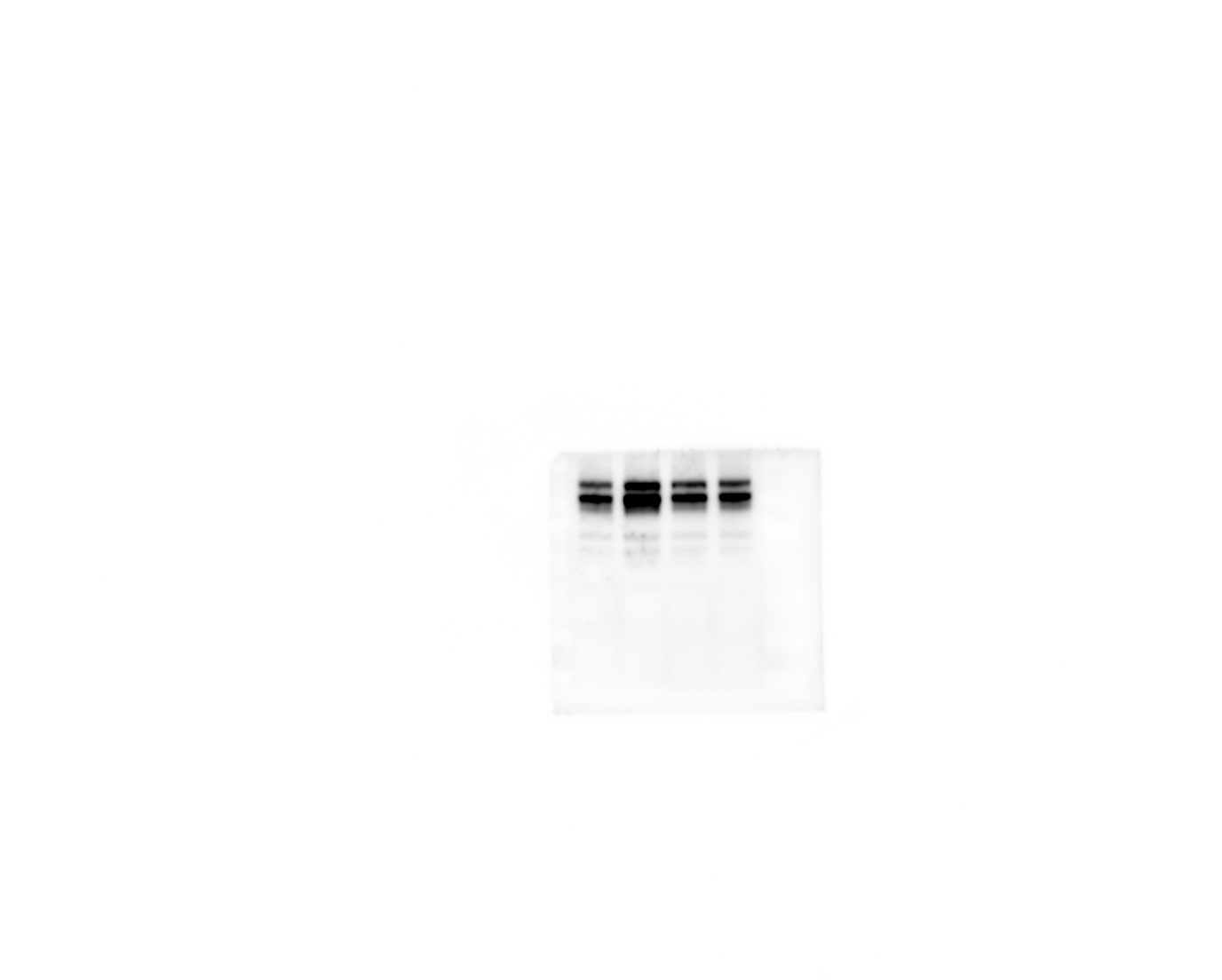

Supplement: Supplementary file 1 [file Data_Sheet_1.zip › the full uncropped Gels and Blots images/Group 1 n=3/N-cadherin/2.Tif]

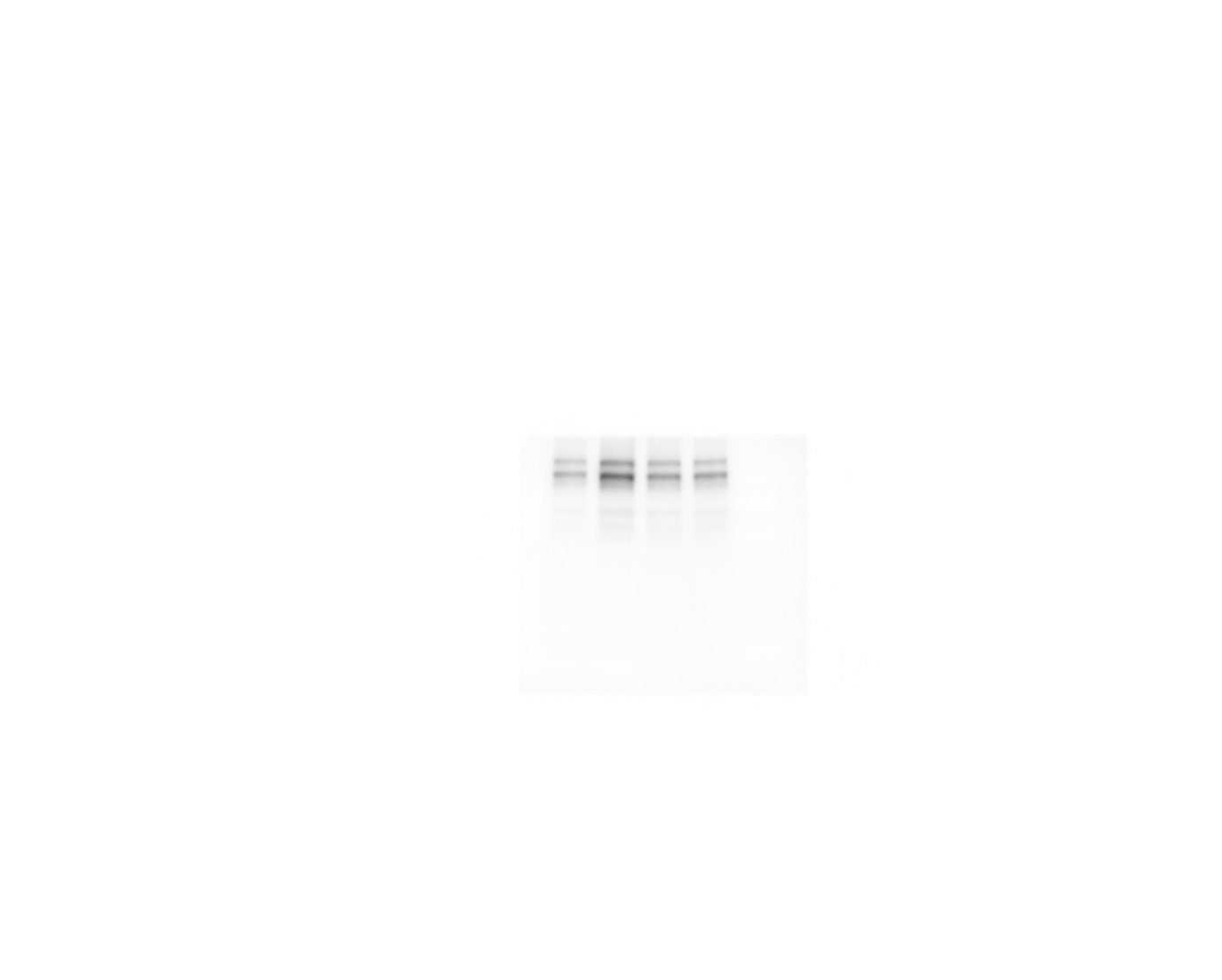

Supplement: Supplementary file 1 [file Data_Sheet_1.zip › the full uncropped Gels and Blots images/Group 1 n=3/N-cadherin/3-2s.Tif]

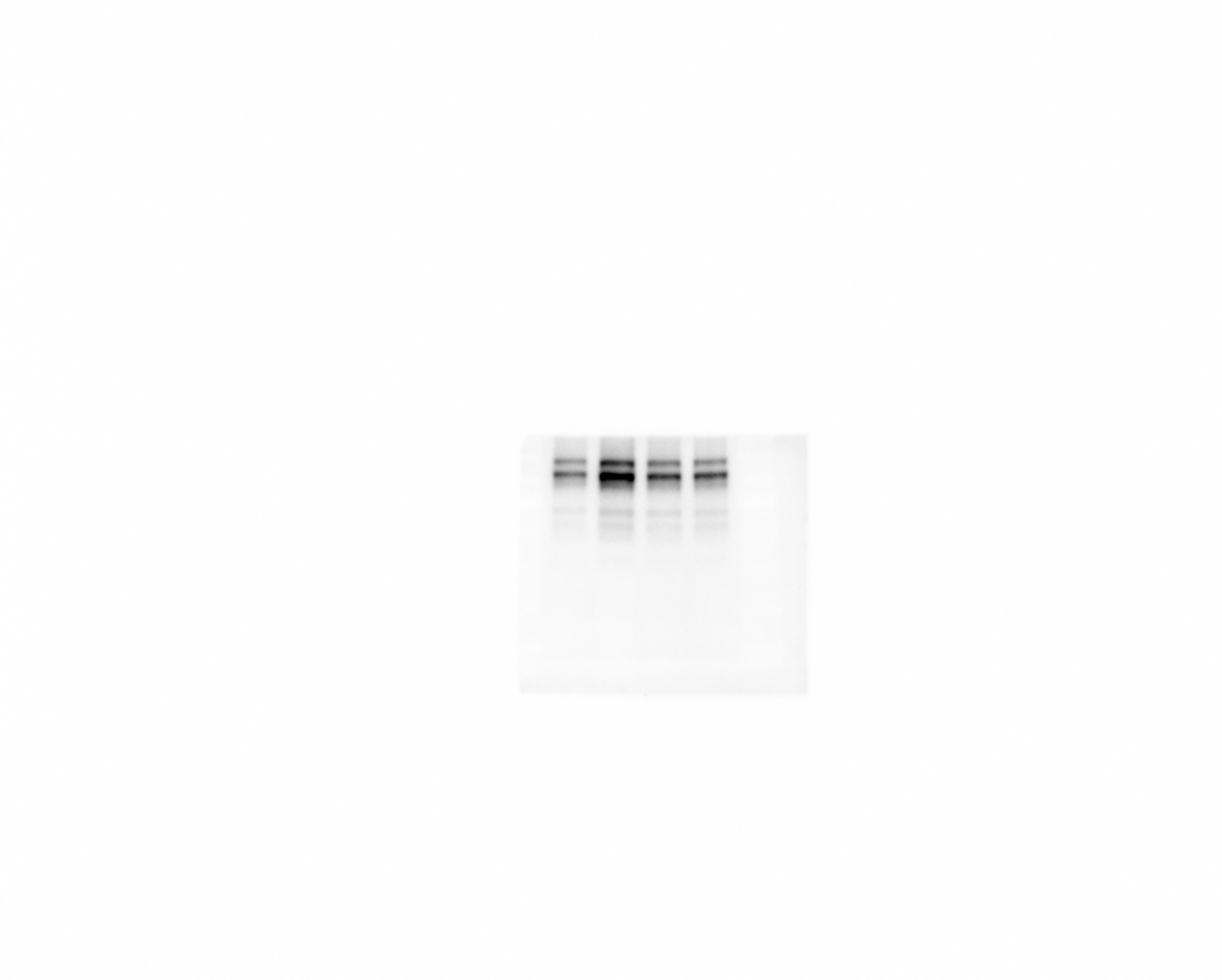

Supplement: Supplementary file 1 [file Data_Sheet_1.zip › the full uncropped Gels and Blots images/Group 1 n=3/N-cadherin/3-4s.Tif]

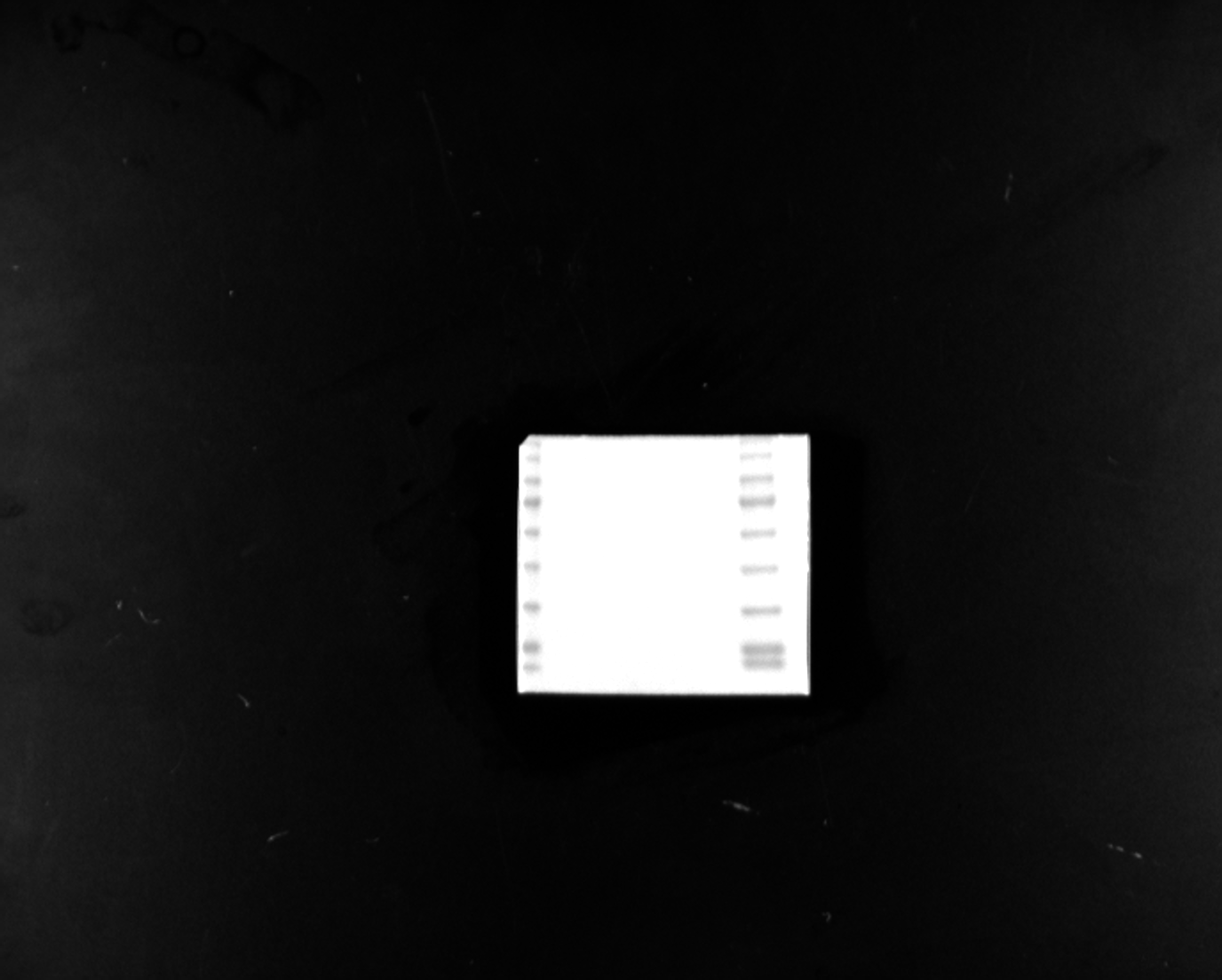

Supplement: Supplementary file 1 [file Data_Sheet_1.zip › the full uncropped Gels and Blots images/Group 1 n=3/N-cadherin/3-t.Tif]

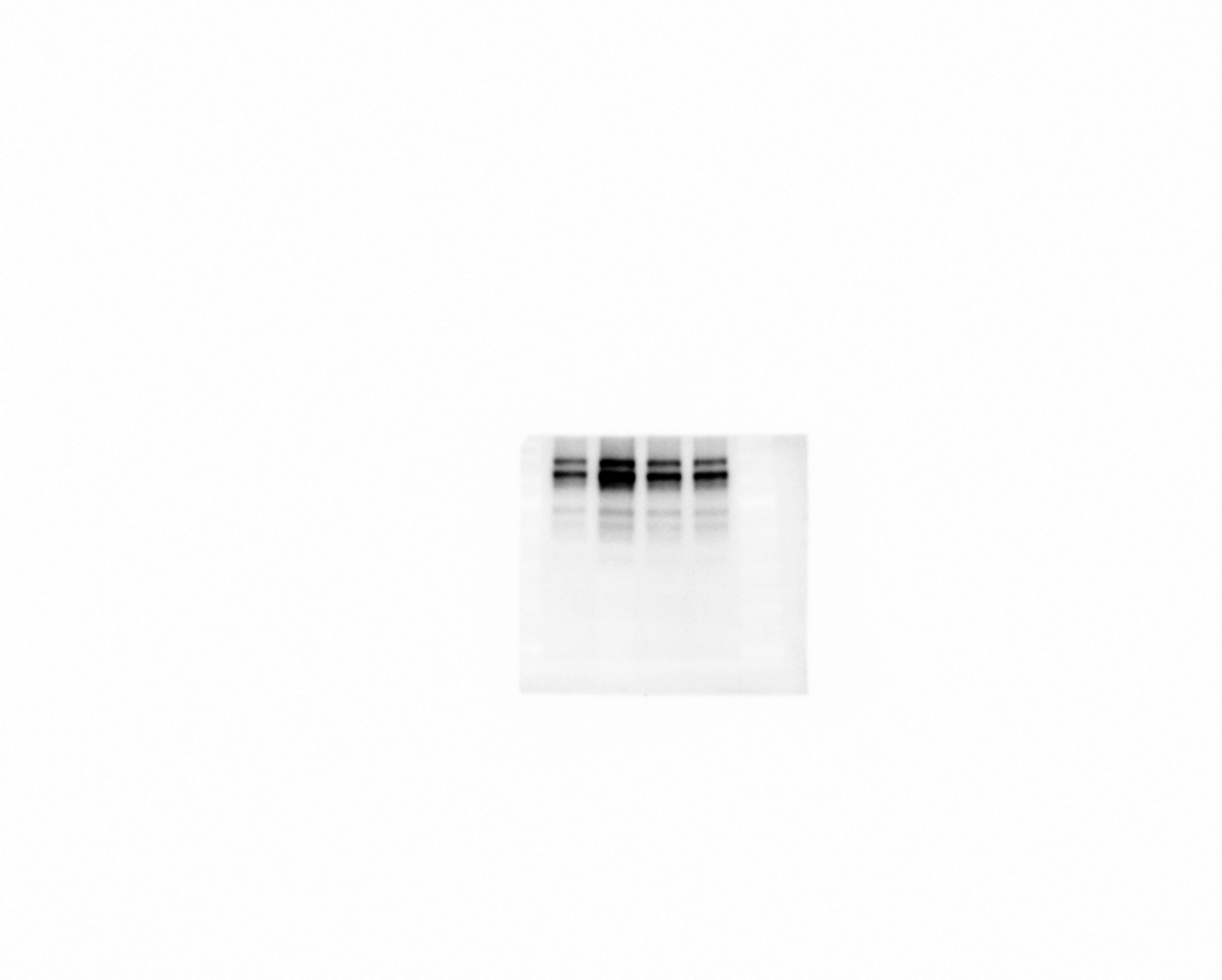

Supplement: Supplementary file 1 [file Data_Sheet_1.zip › the full uncropped Gels and Blots images/Group 1 n=3/N-cadherin/3.Tif]

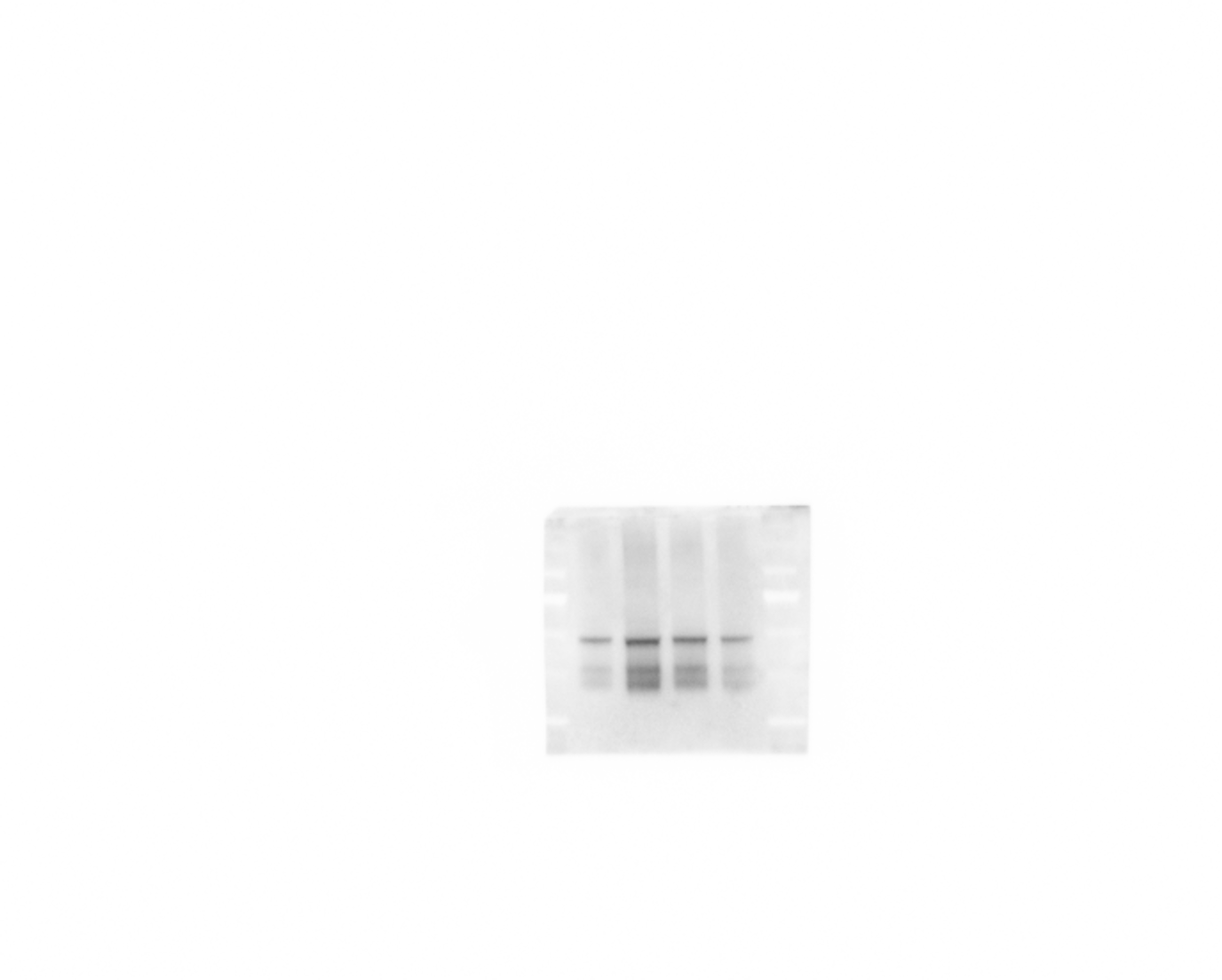

Supplement: Supplementary file 1 [file Data_Sheet_1.zip › the full uncropped Gels and Blots images/Group 1 n=3/Vimentin/1-3s.Tif]

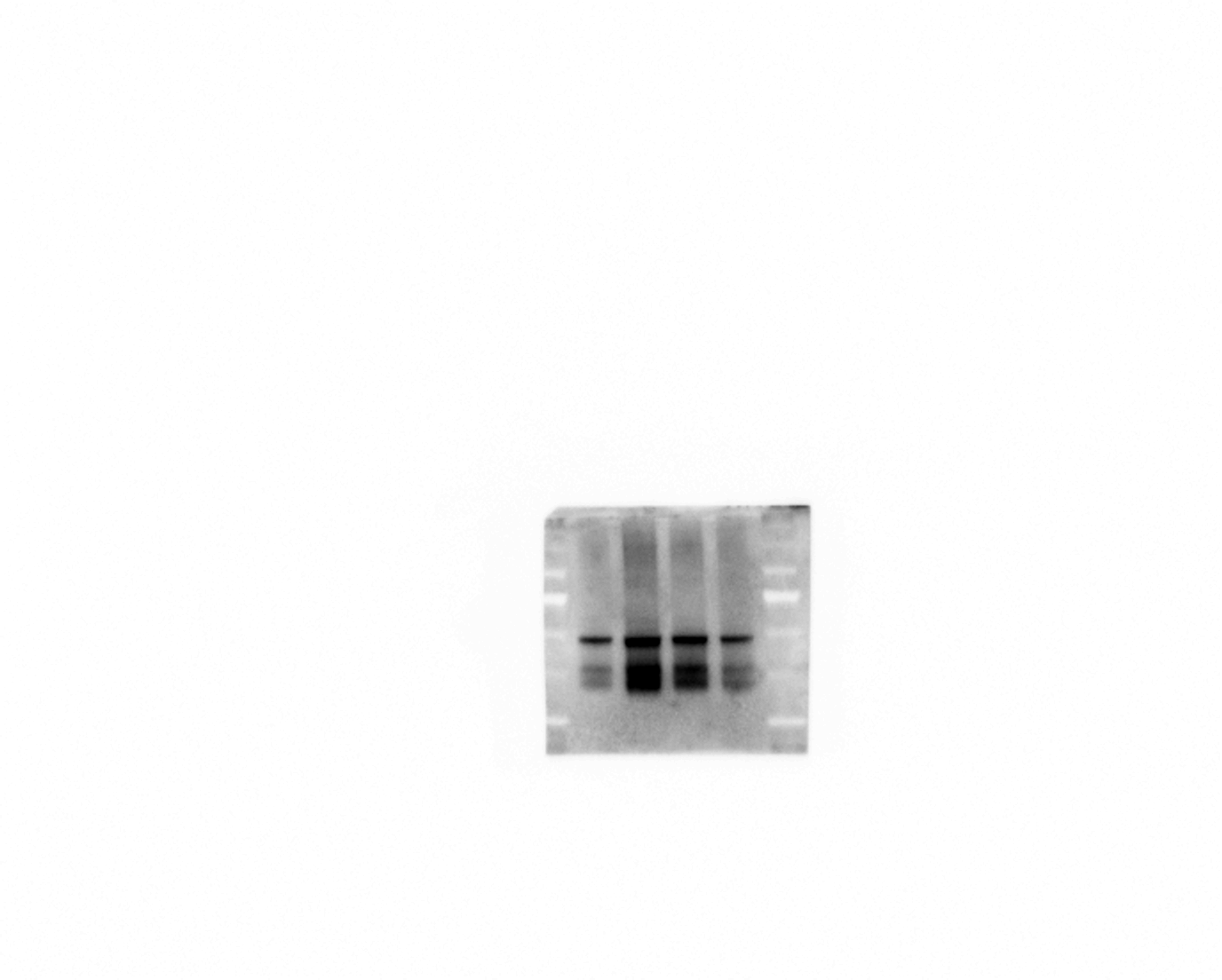

Supplement: Supplementary file 1 [file Data_Sheet_1.zip › the full uncropped Gels and Blots images/Group 1 n=3/Vimentin/1-5s.Tif]

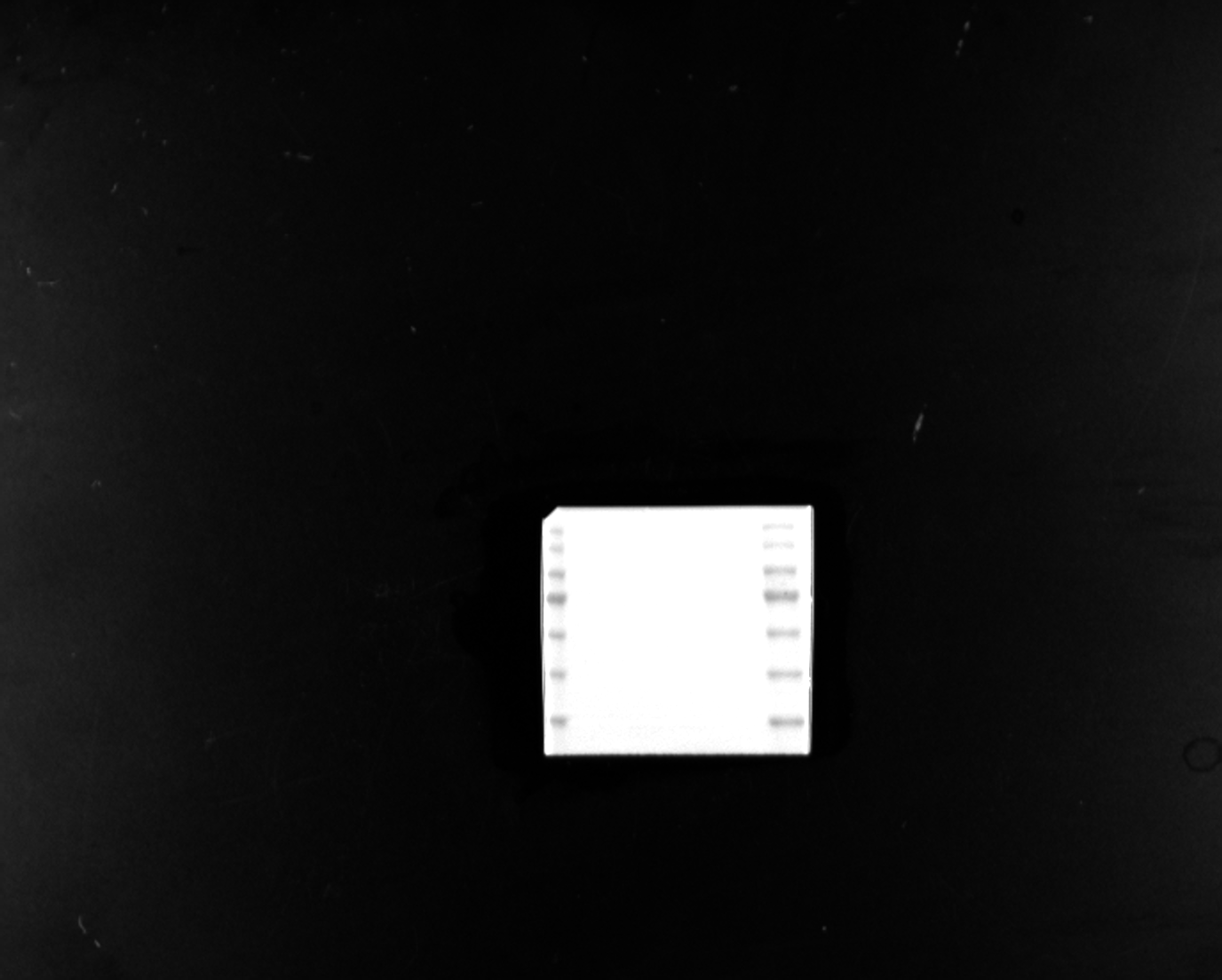

Supplement: Supplementary file 1 [file Data_Sheet_1.zip › the full uncropped Gels and Blots images/Group 1 n=3/Vimentin/1-t.Tif]

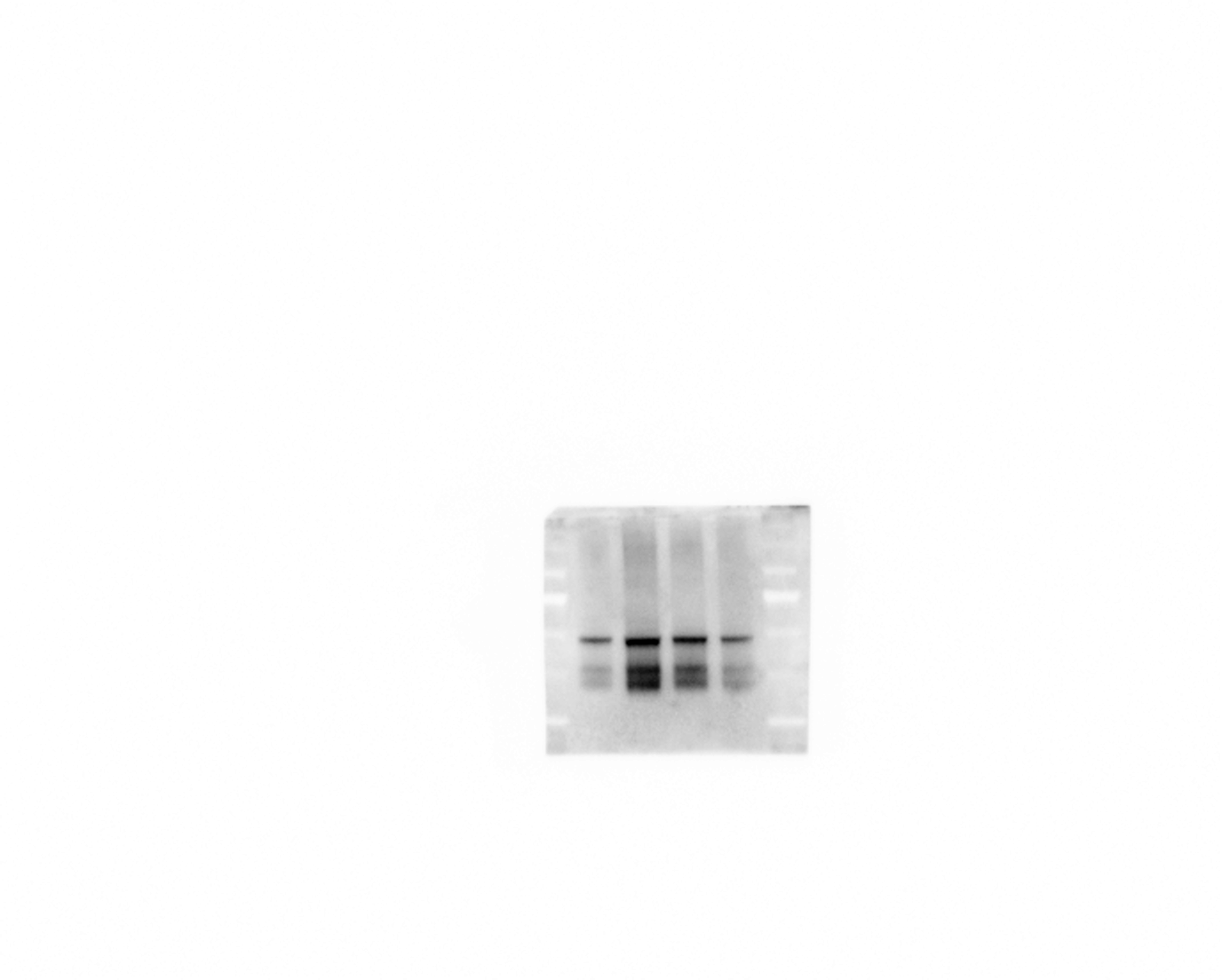

Supplement: Supplementary file 1 [file Data_Sheet_1.zip › the full uncropped Gels and Blots images/Group 1 n=3/Vimentin/1.Tif]

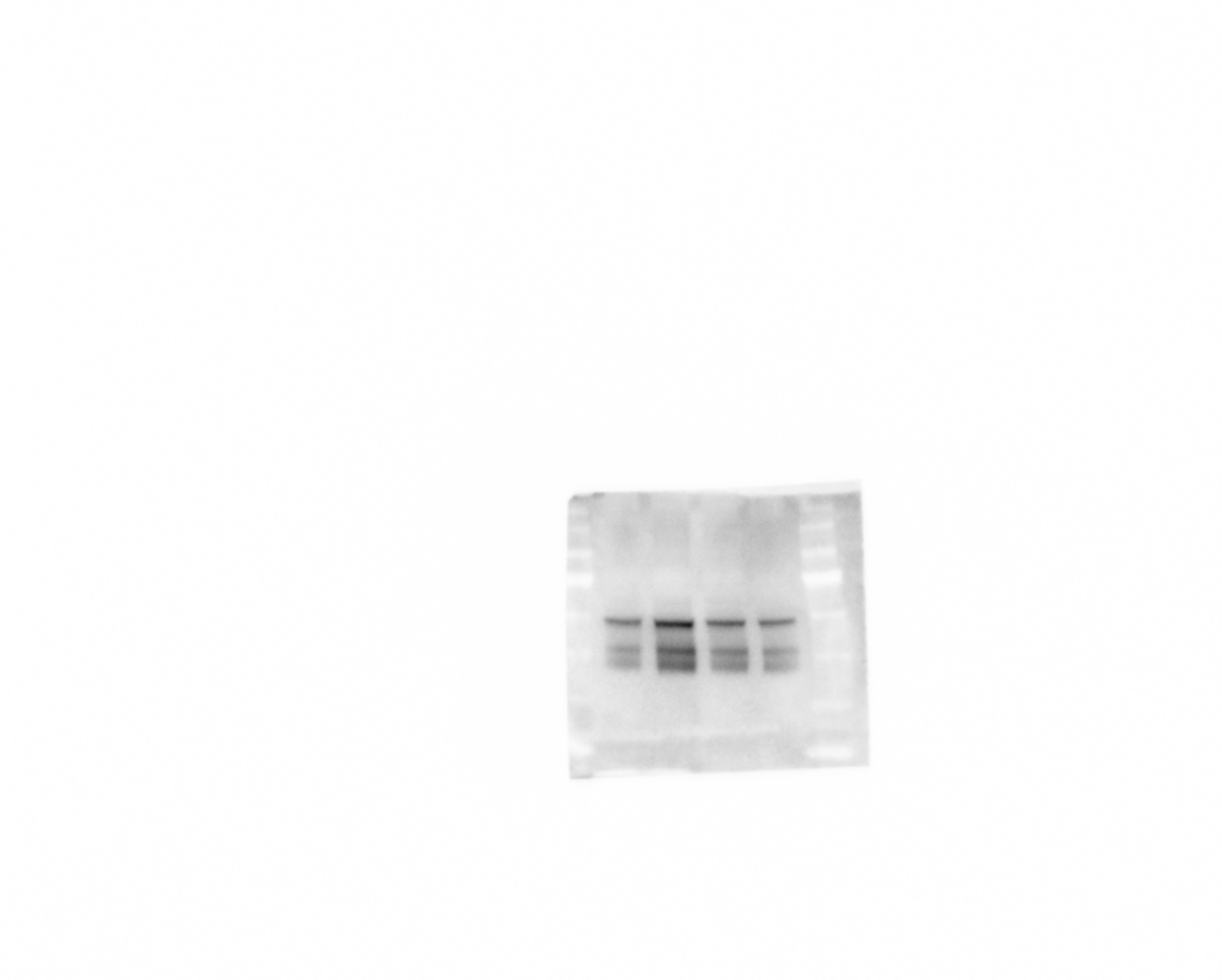

Supplement: Supplementary file 1 [file Data_Sheet_1.zip › the full uncropped Gels and Blots images/Group 1 n=3/Vimentin/2-3s.Tif]

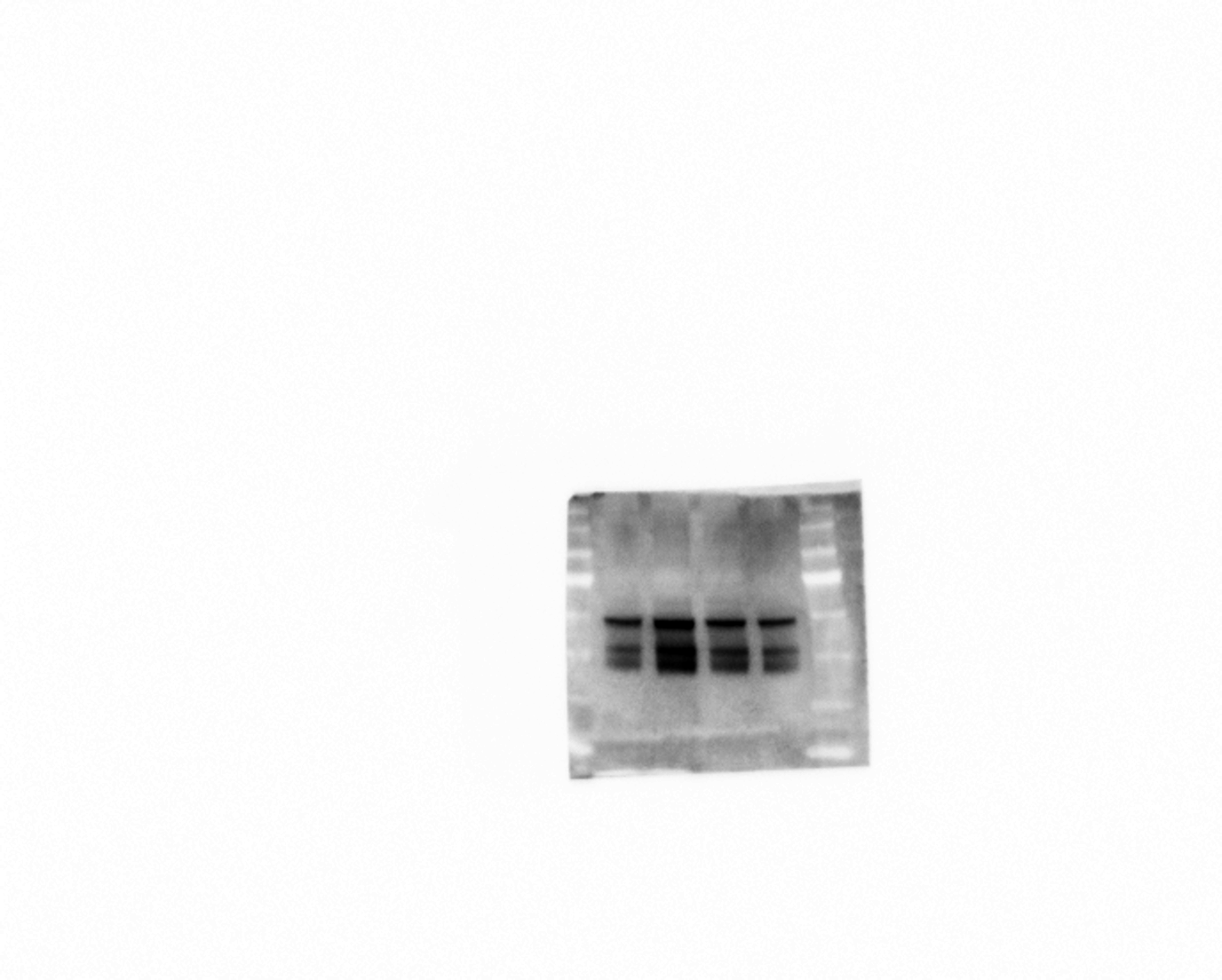

Supplement: Supplementary file 1 [file Data_Sheet_1.zip › the full uncropped Gels and Blots images/Group 1 n=3/Vimentin/2-8s.Tif]

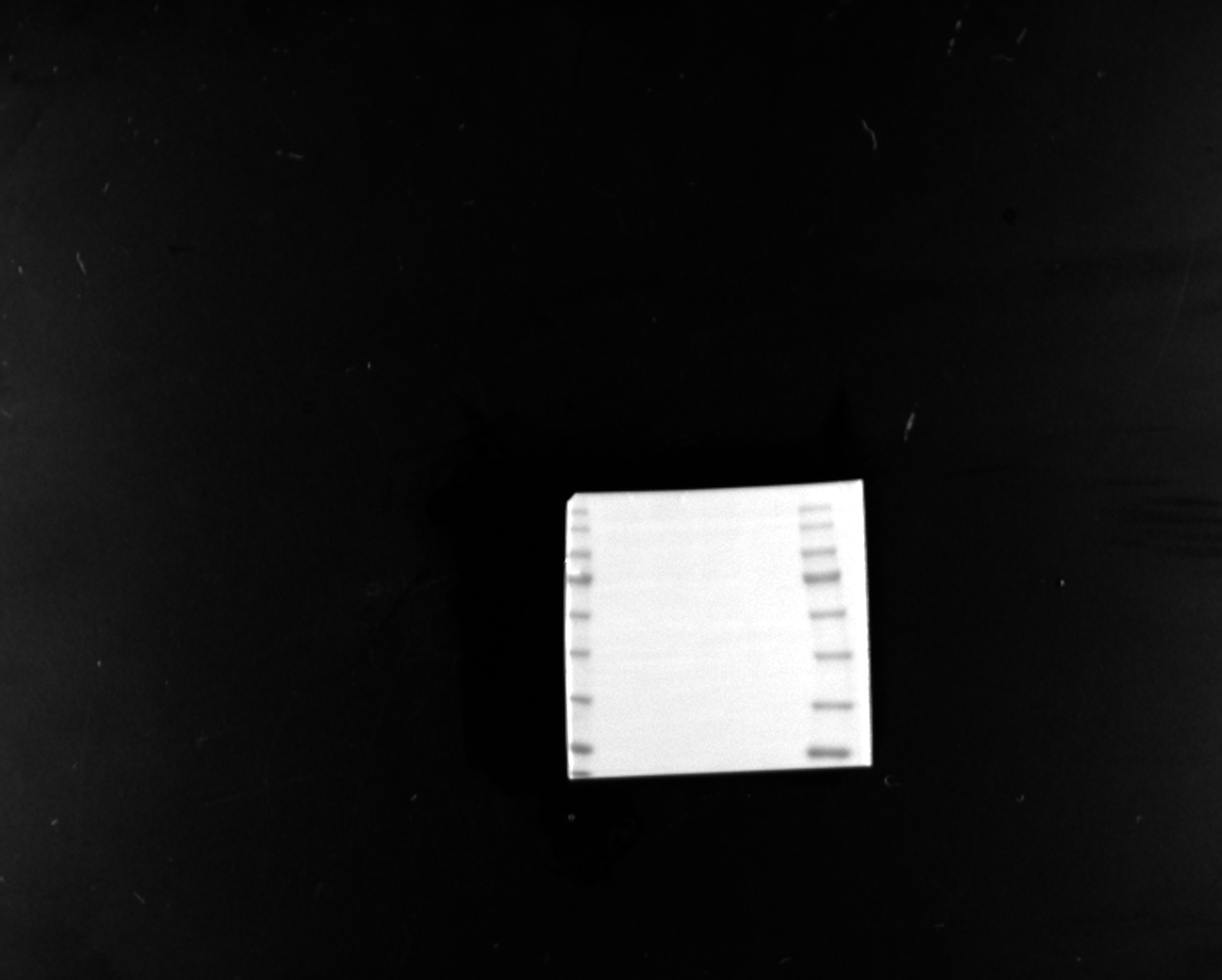

Supplement: Supplementary file 1 [file Data_Sheet_1.zip › the full uncropped Gels and Blots images/Group 1 n=3/Vimentin/2-t.Tif]

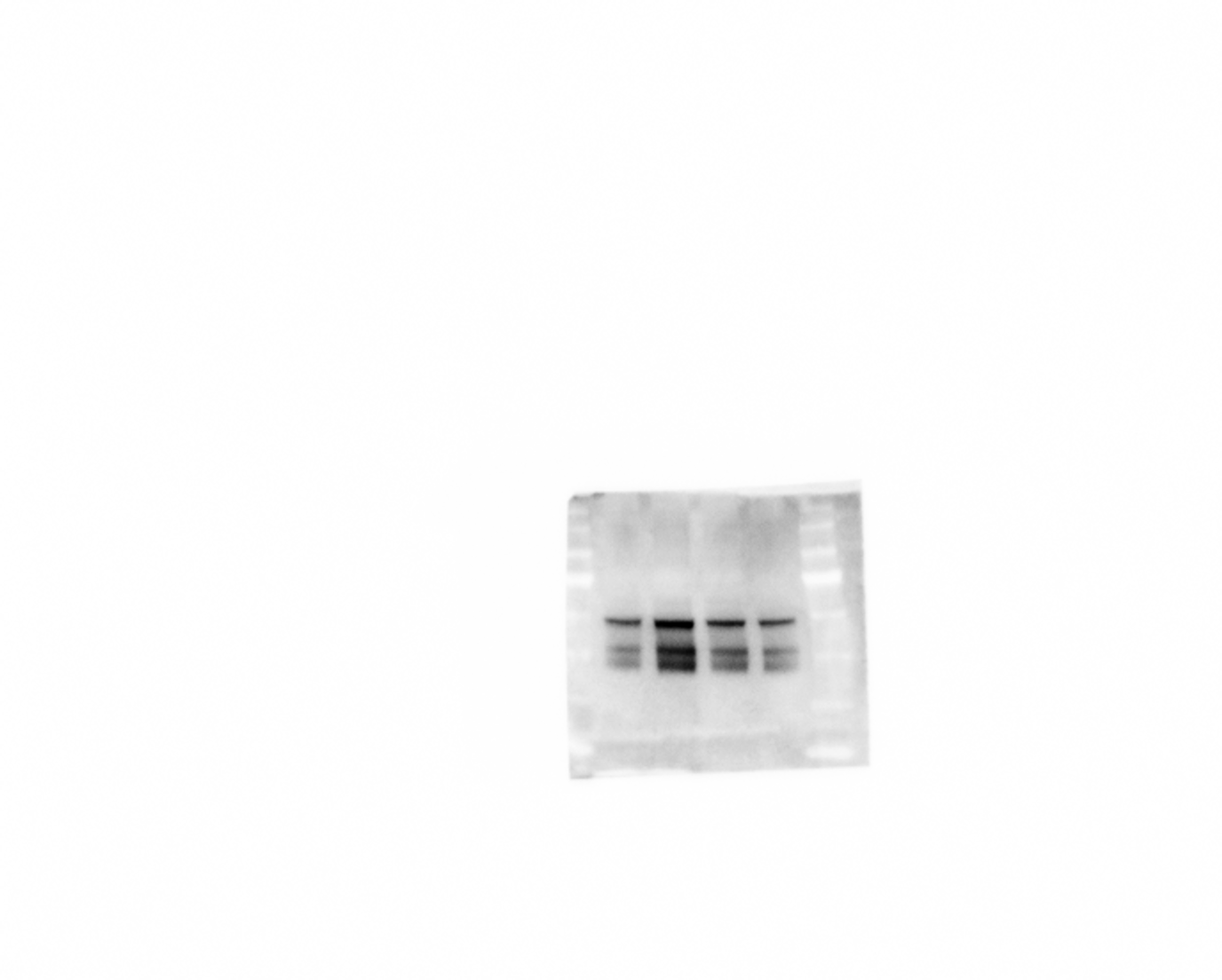

Supplement: Supplementary file 1 [file Data_Sheet_1.zip › the full uncropped Gels and Blots images/Group 1 n=3/Vimentin/2.Tif]

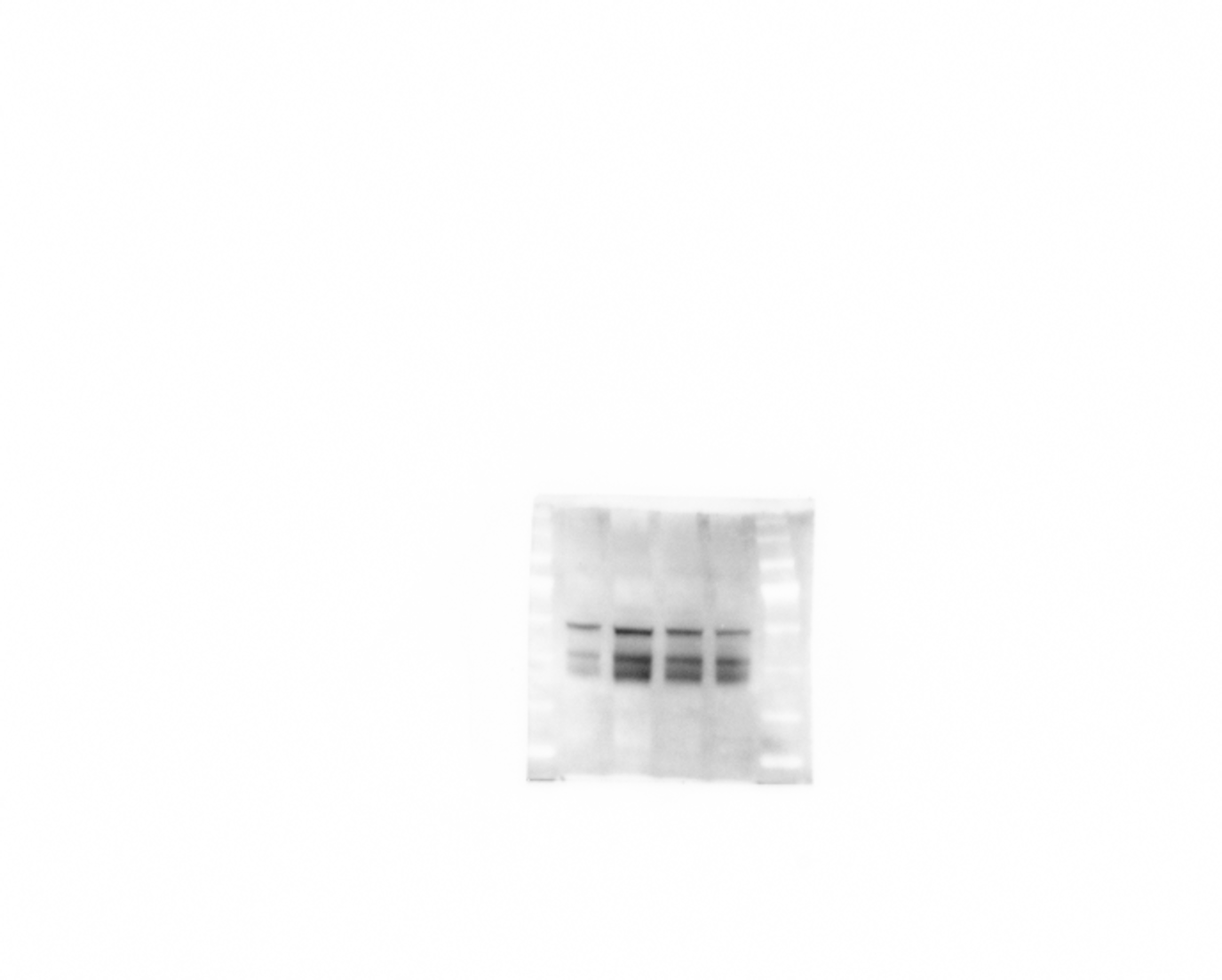

Supplement: Supplementary file 1 [file Data_Sheet_1.zip › the full uncropped Gels and Blots images/Group 1 n=3/Vimentin/3-3s.Tif]

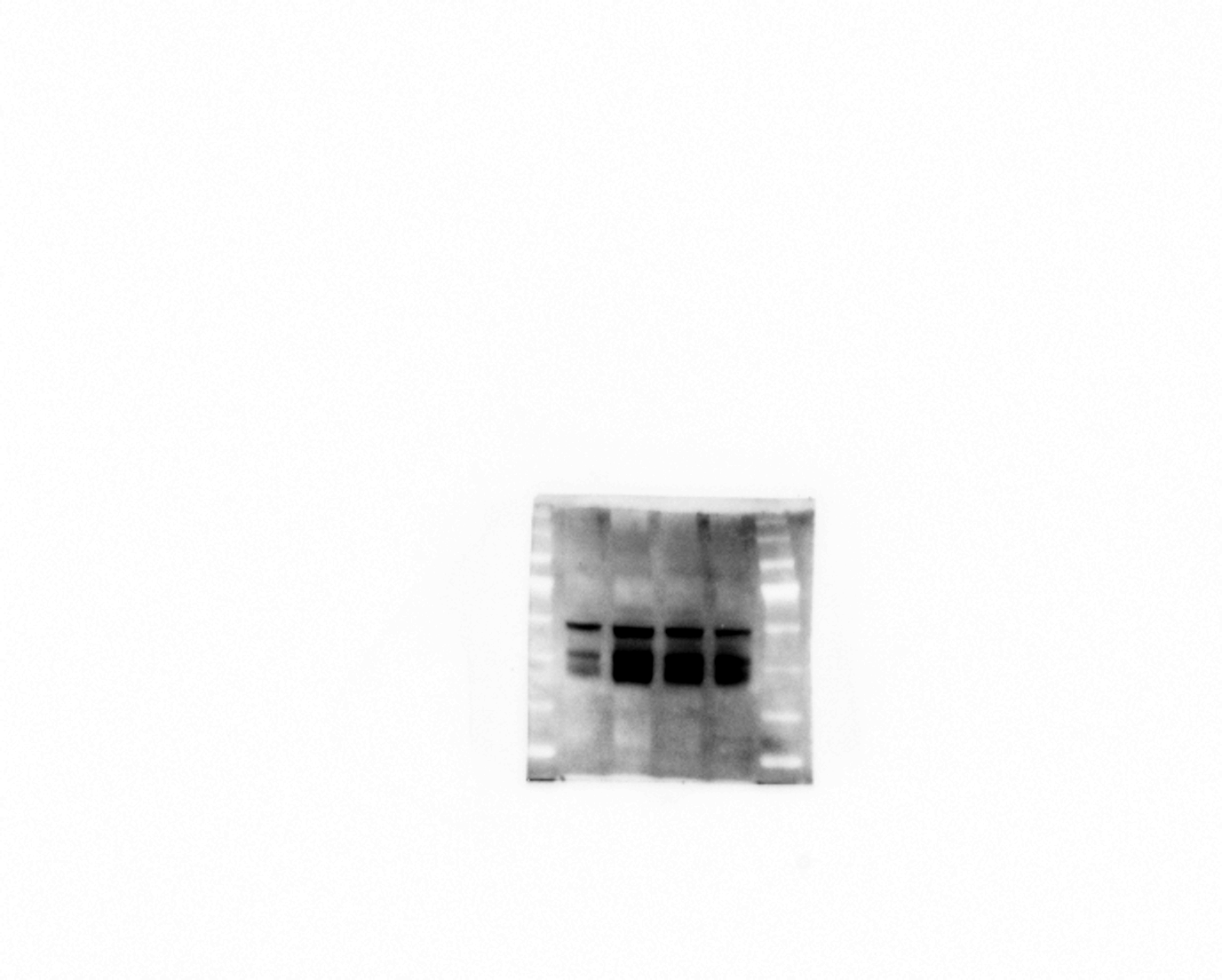

Supplement: Supplementary file 1 [file Data_Sheet_1.zip › the full uncropped Gels and Blots images/Group 1 n=3/Vimentin/3-9s.Tif]

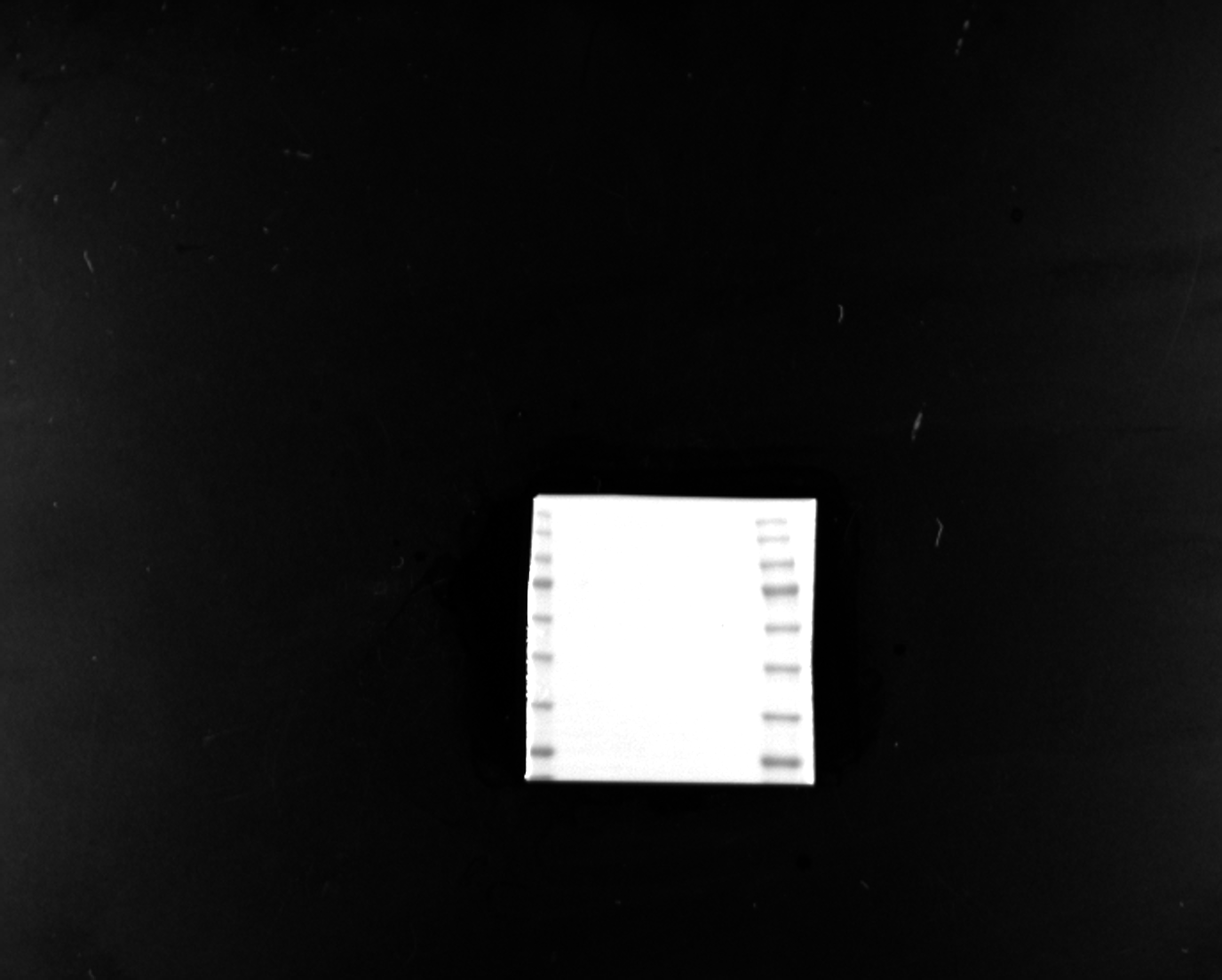

Supplement: Supplementary file 1 [file Data_Sheet_1.zip › the full uncropped Gels and Blots images/Group 1 n=3/Vimentin/3-t.Tif]

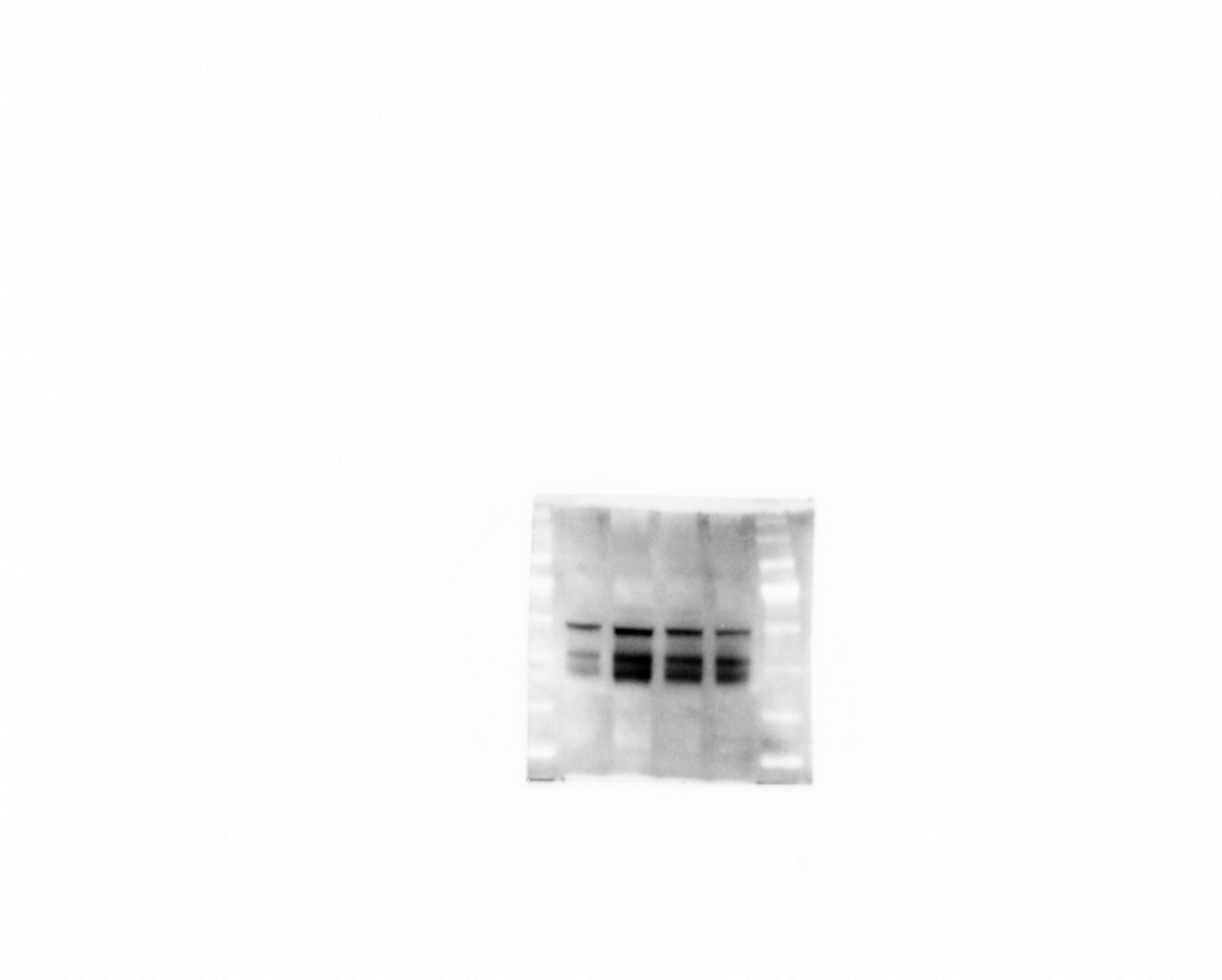

Supplement: Supplementary file 1 [file Data_Sheet_1.zip › the full uncropped Gels and Blots images/Group 1 n=3/Vimentin/3.Tif]

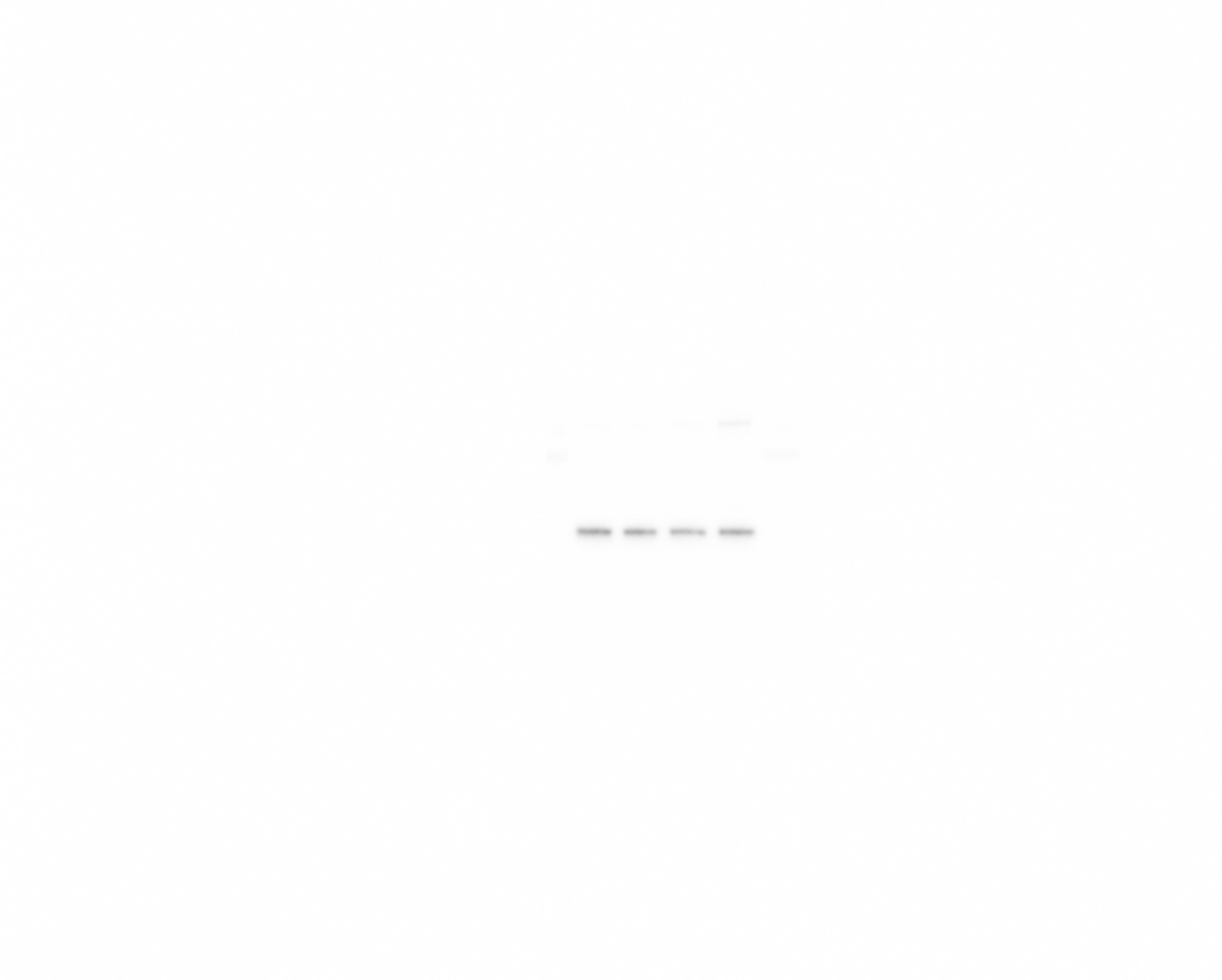

Supplement: Supplementary file 1 [file Data_Sheet_1.zip › the full uncropped Gels and Blots images/Group 1 n=3/β-actin/1-2s.Tif]

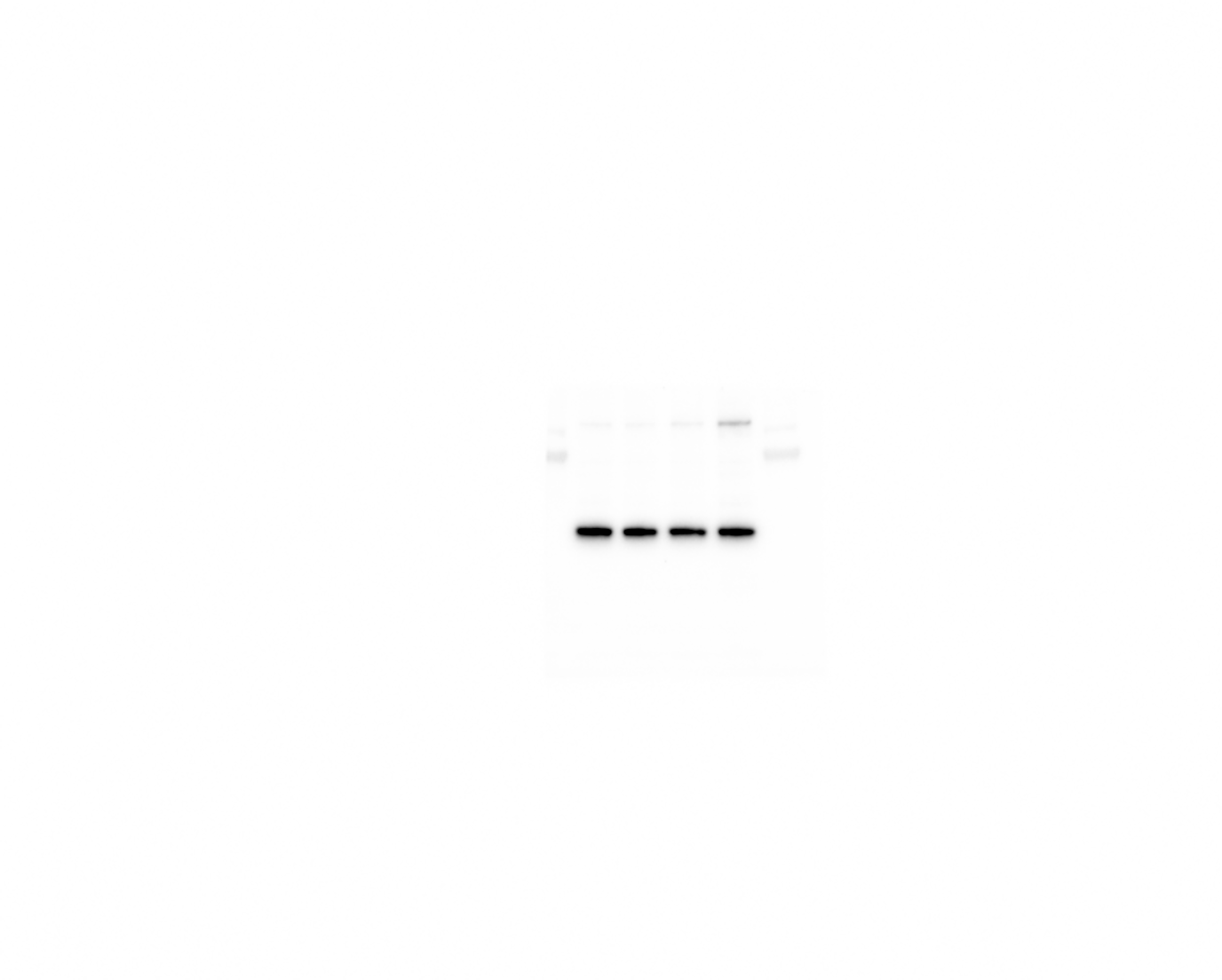

Supplement: Supplementary file 1 [file Data_Sheet_1.zip › the full uncropped Gels and Blots images/Group 1 n=3/β-actin/1-3s.Tif]

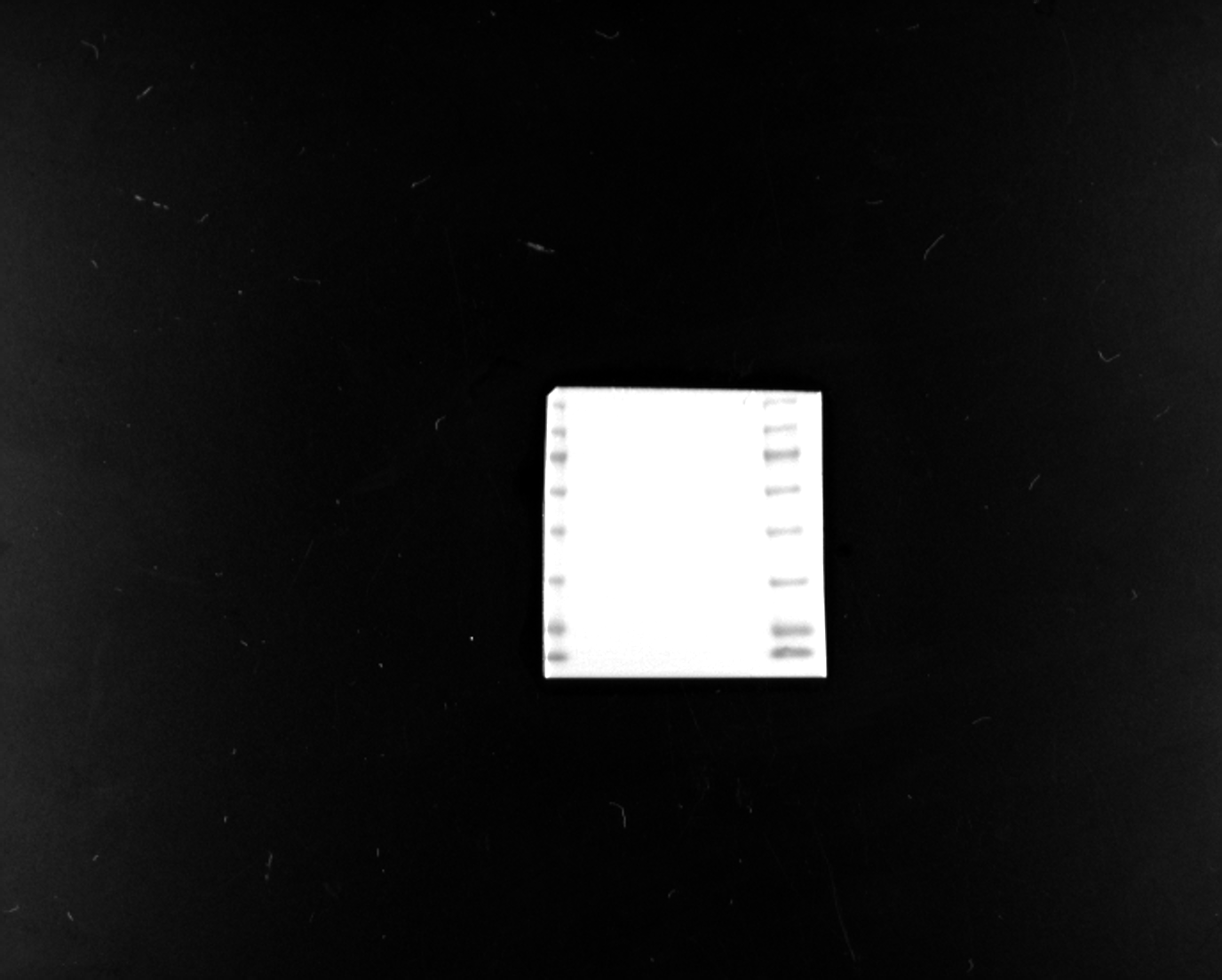

Supplement: Supplementary file 1 [file Data_Sheet_1.zip › the full uncropped Gels and Blots images/Group 1 n=3/β-actin/1-t.Tif]

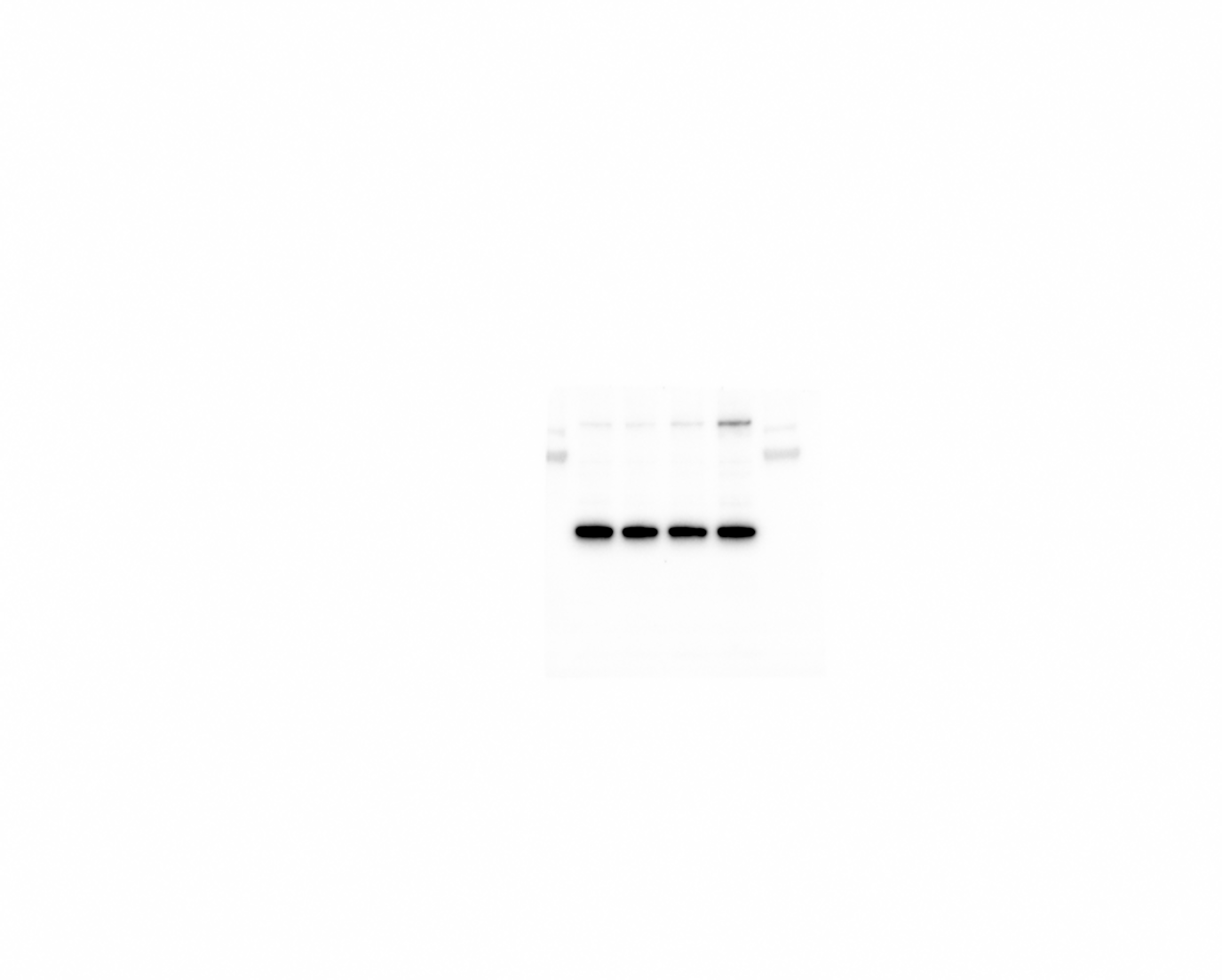

Supplement: Supplementary file 1 [file Data_Sheet_1.zip › the full uncropped Gels and Blots images/Group 1 n=3/β-actin/1.Tif]

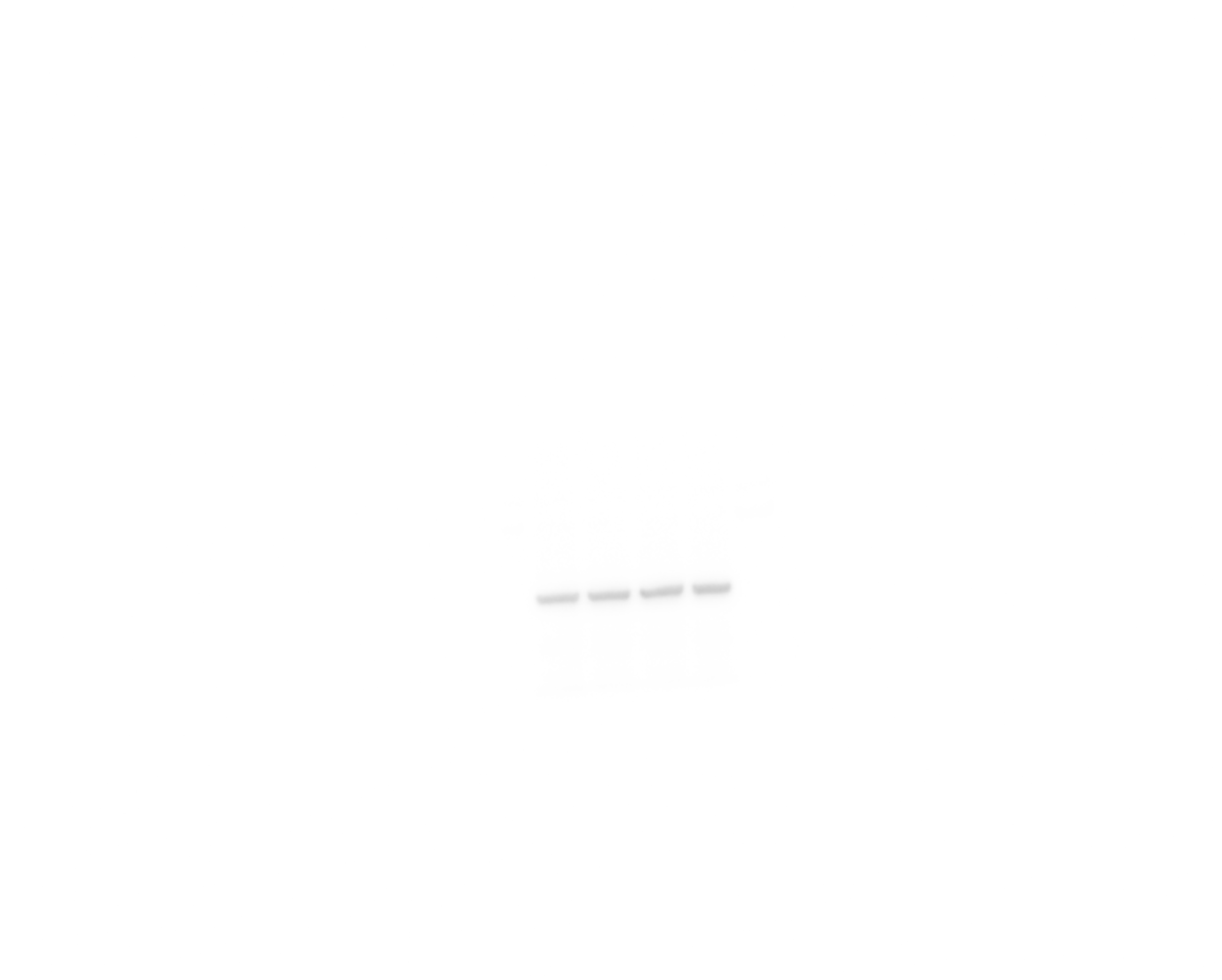

Supplement: Supplementary file 1 [file Data_Sheet_1.zip › the full uncropped Gels and Blots images/Group 1 n=3/β-actin/2-2s.Tif]

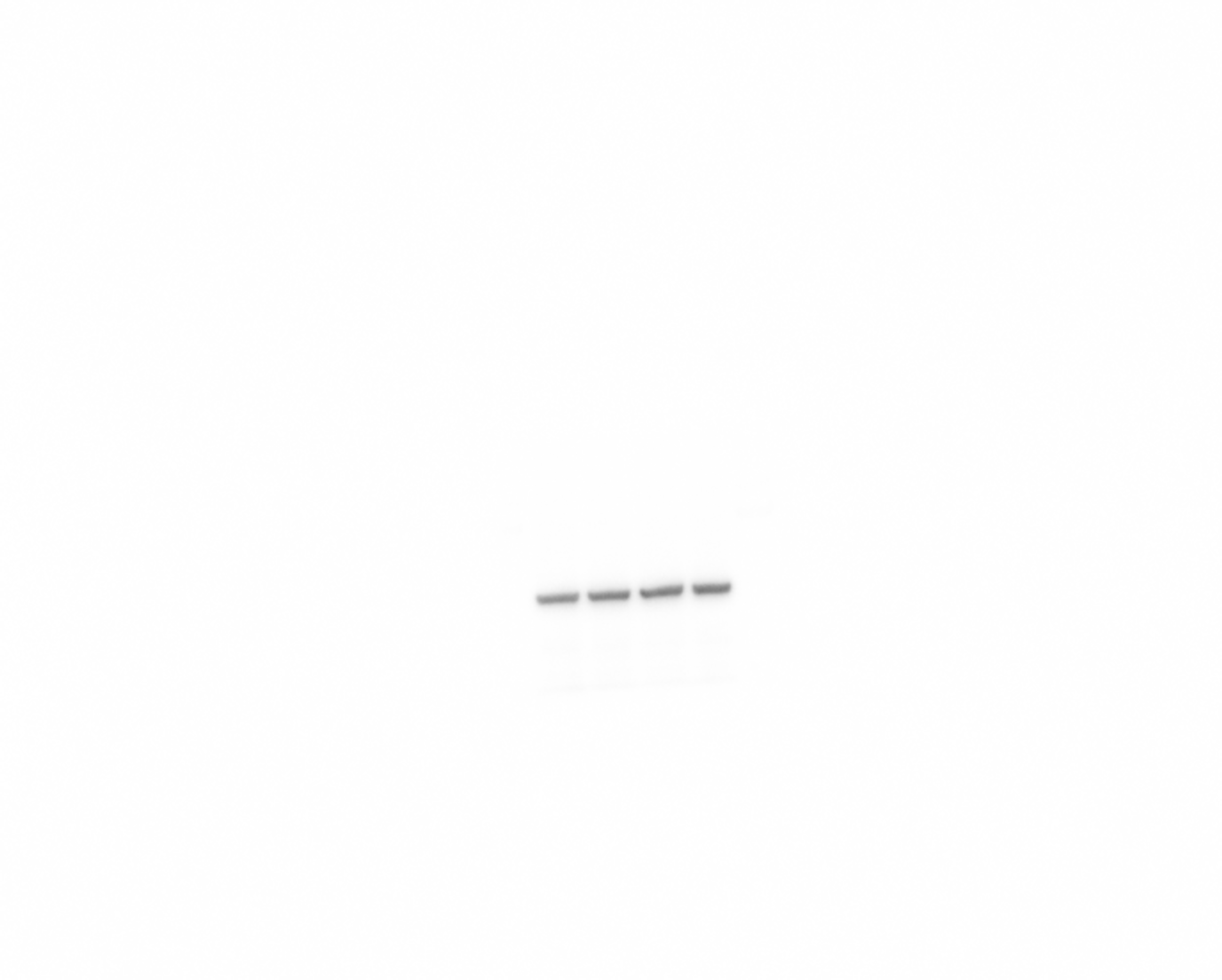

Supplement: Supplementary file 1 [file Data_Sheet_1.zip › the full uncropped Gels and Blots images/Group 1 n=3/β-actin/2-3s.Tif]

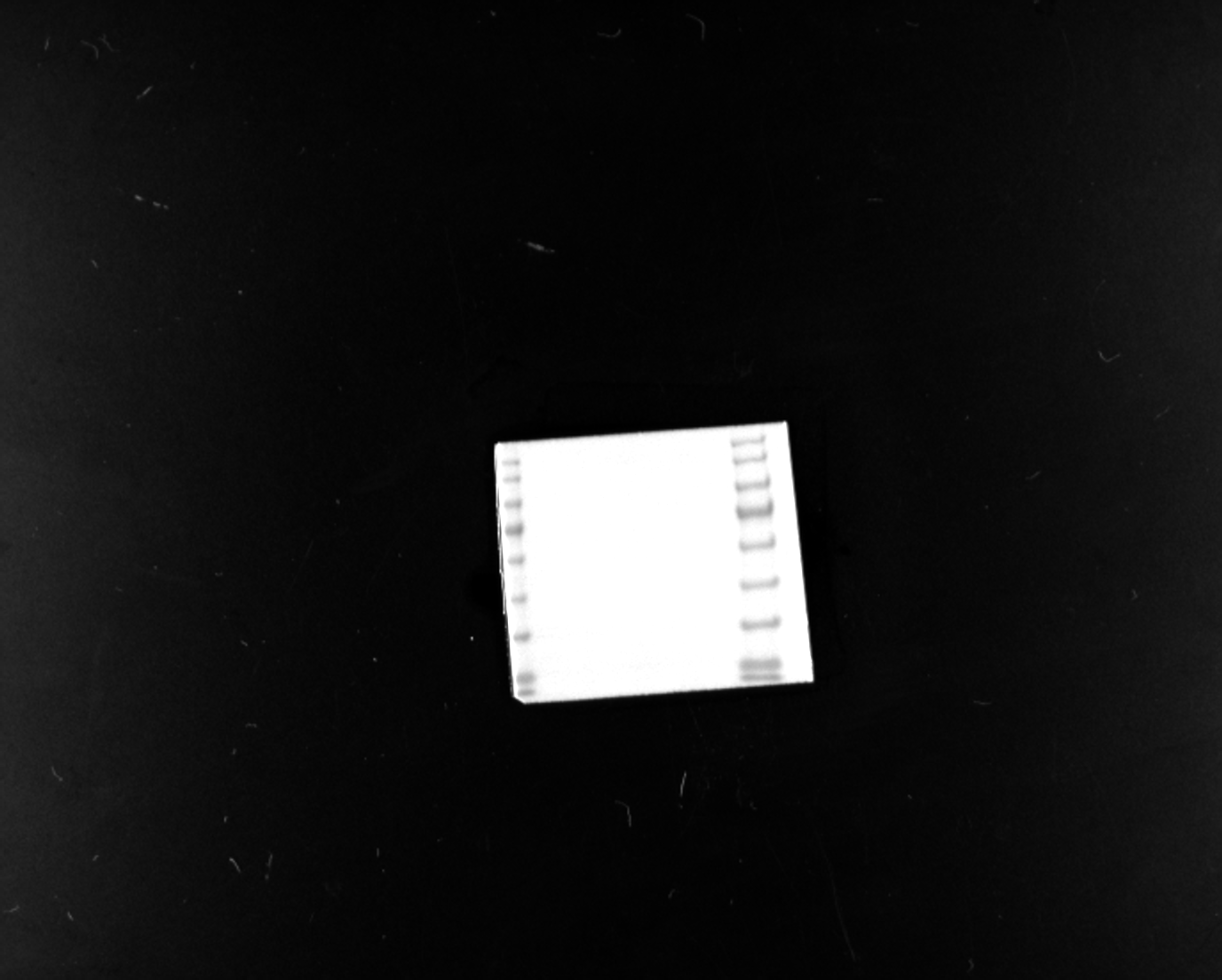

Supplement: Supplementary file 1 [file Data_Sheet_1.zip › the full uncropped Gels and Blots images/Group 1 n=3/β-actin/2-t.Tif]

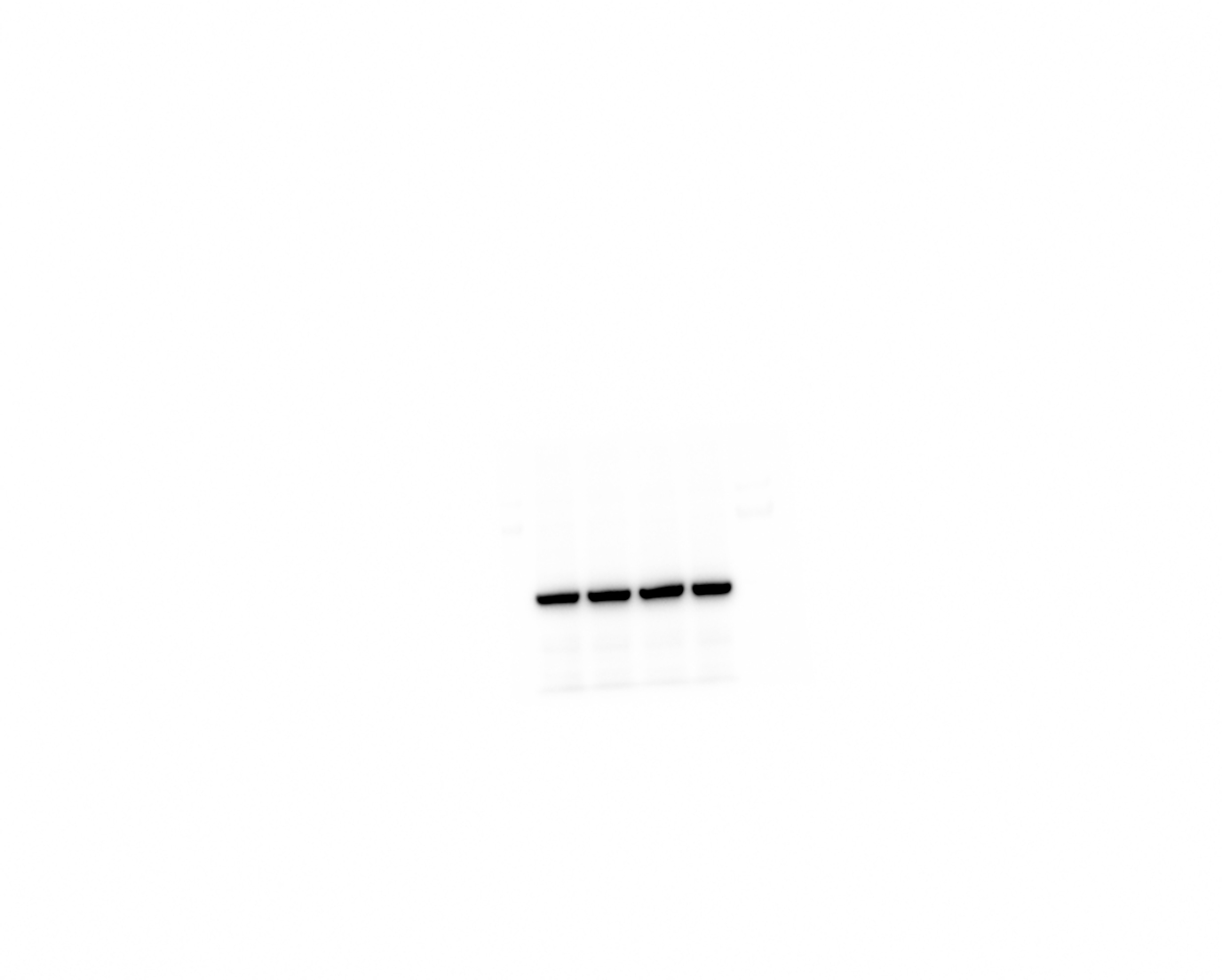

Supplement: Supplementary file 1 [file Data_Sheet_1.zip › the full uncropped Gels and Blots images/Group 1 n=3/β-actin/2.Tif]

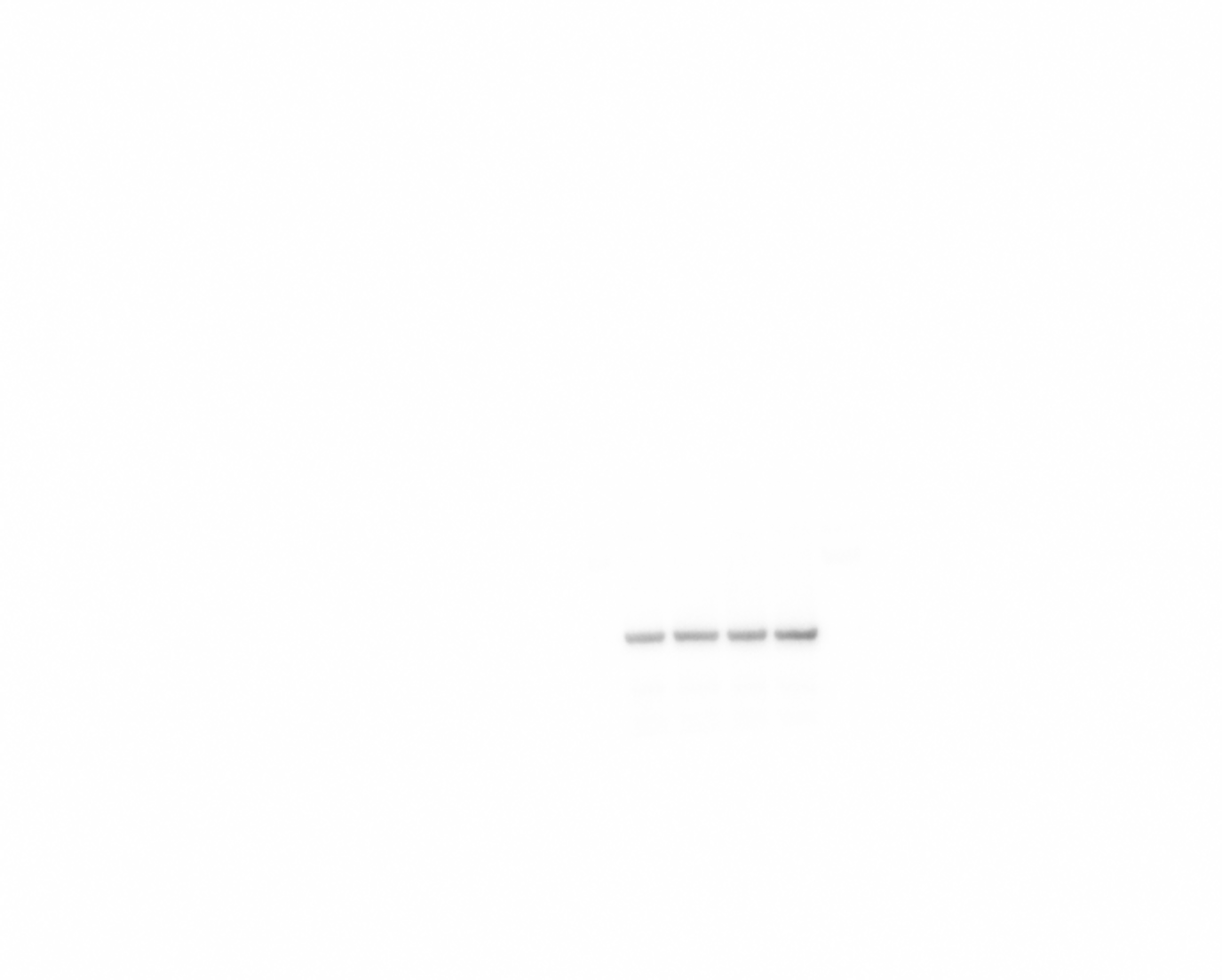

Supplement: Supplementary file 1 [file Data_Sheet_1.zip › the full uncropped Gels and Blots images/Group 1 n=3/β-actin/3-2s.Tif]

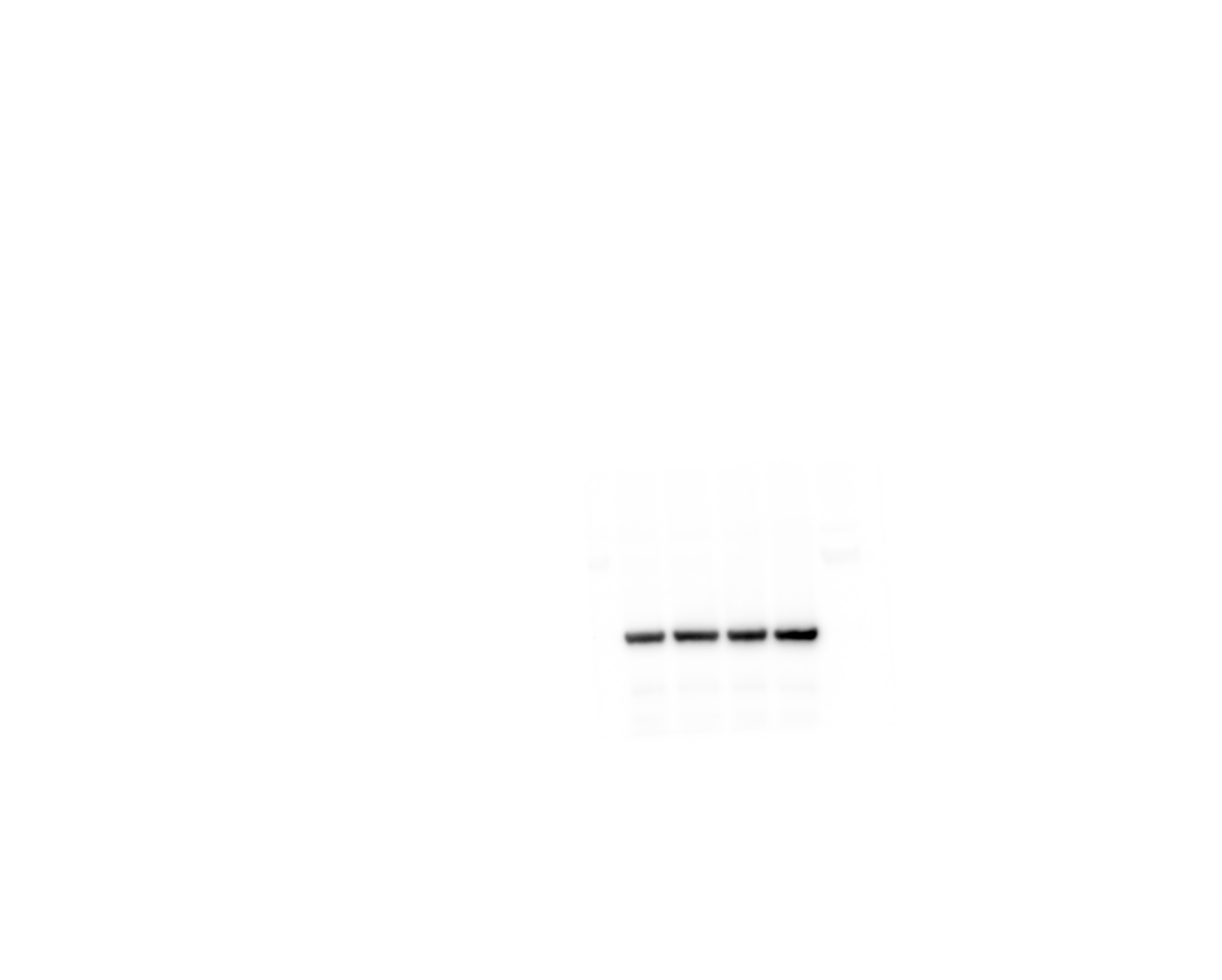

Supplement: Supplementary file 1 [file Data_Sheet_1.zip › the full uncropped Gels and Blots images/Group 1 n=3/β-actin/3-3s.Tif]

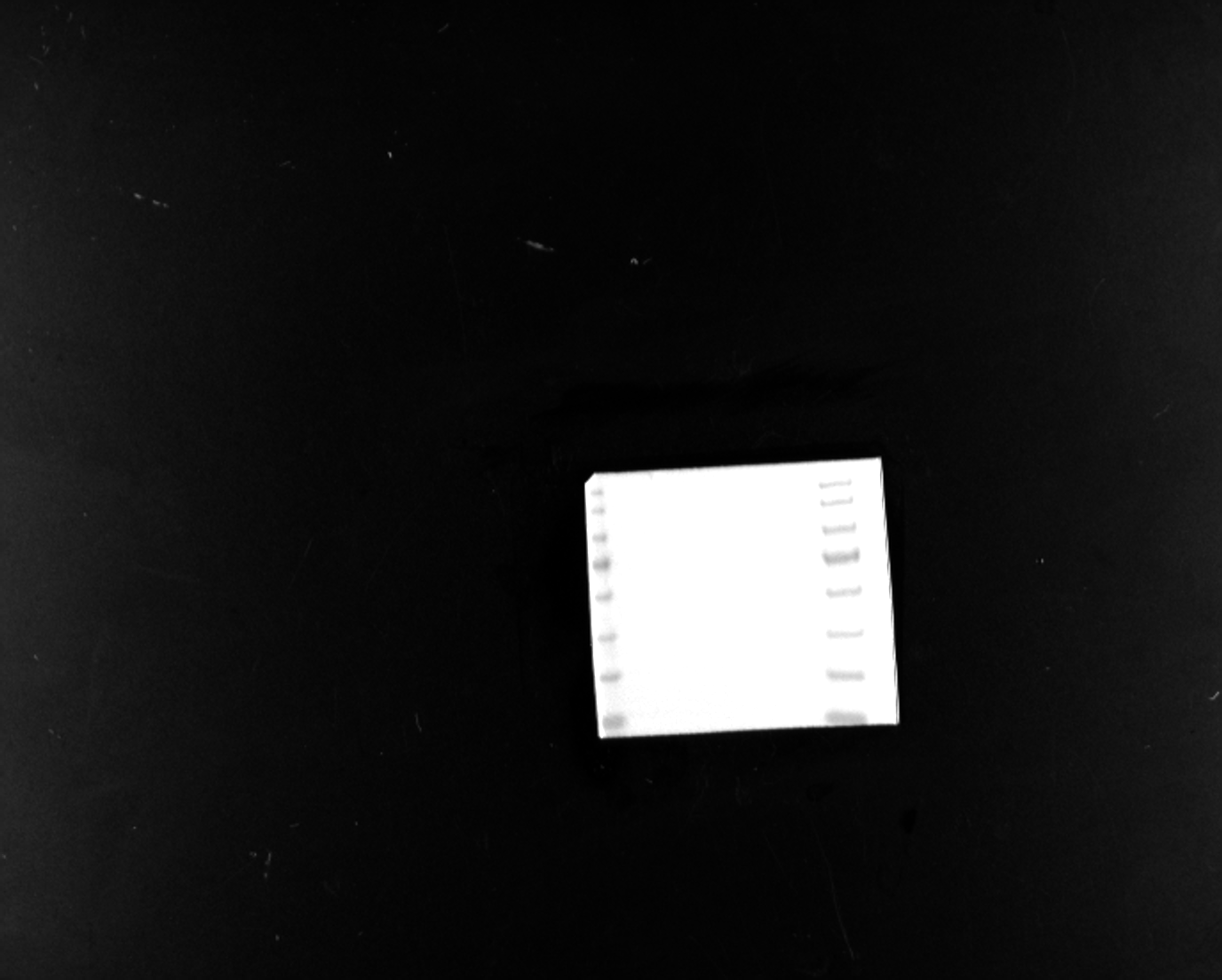

Supplement: Supplementary file 1 [file Data_Sheet_1.zip › the full uncropped Gels and Blots images/Group 1 n=3/β-actin/3-t.Tif]

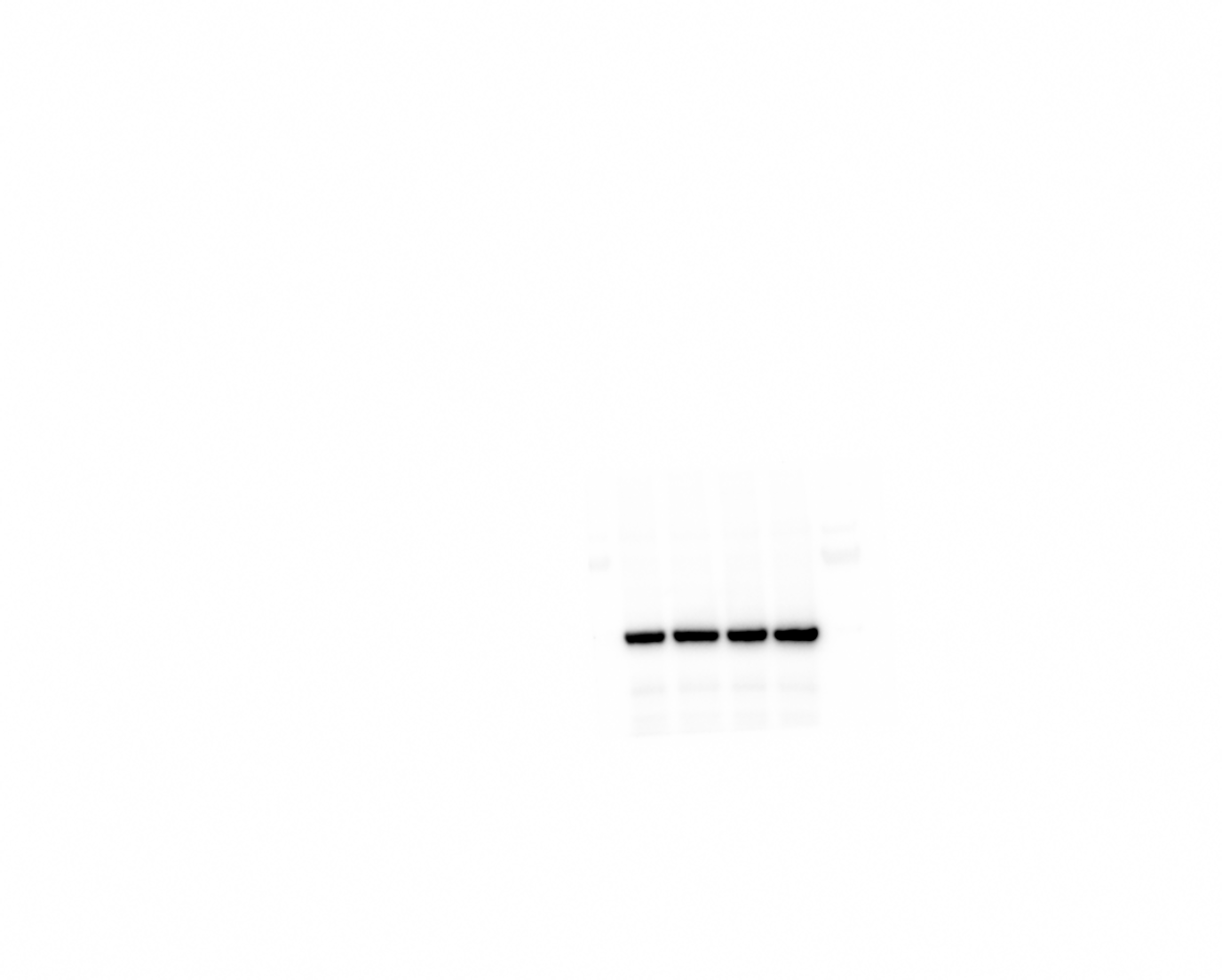

Supplement: Supplementary file 1 [file Data_Sheet_1.zip › the full uncropped Gels and Blots images/Group 1 n=3/β-actin/3.Tif]

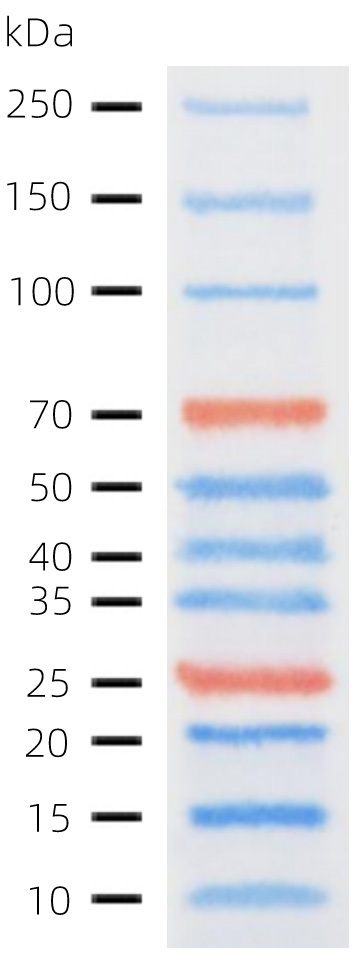

Supplement: Supplementary file 1 [file Data_Sheet_1.zip › the full uncropped Gels and Blots images/Group 2 n=3/ColorMixed Protein Marker 250 (10-250 kDa).jpg]

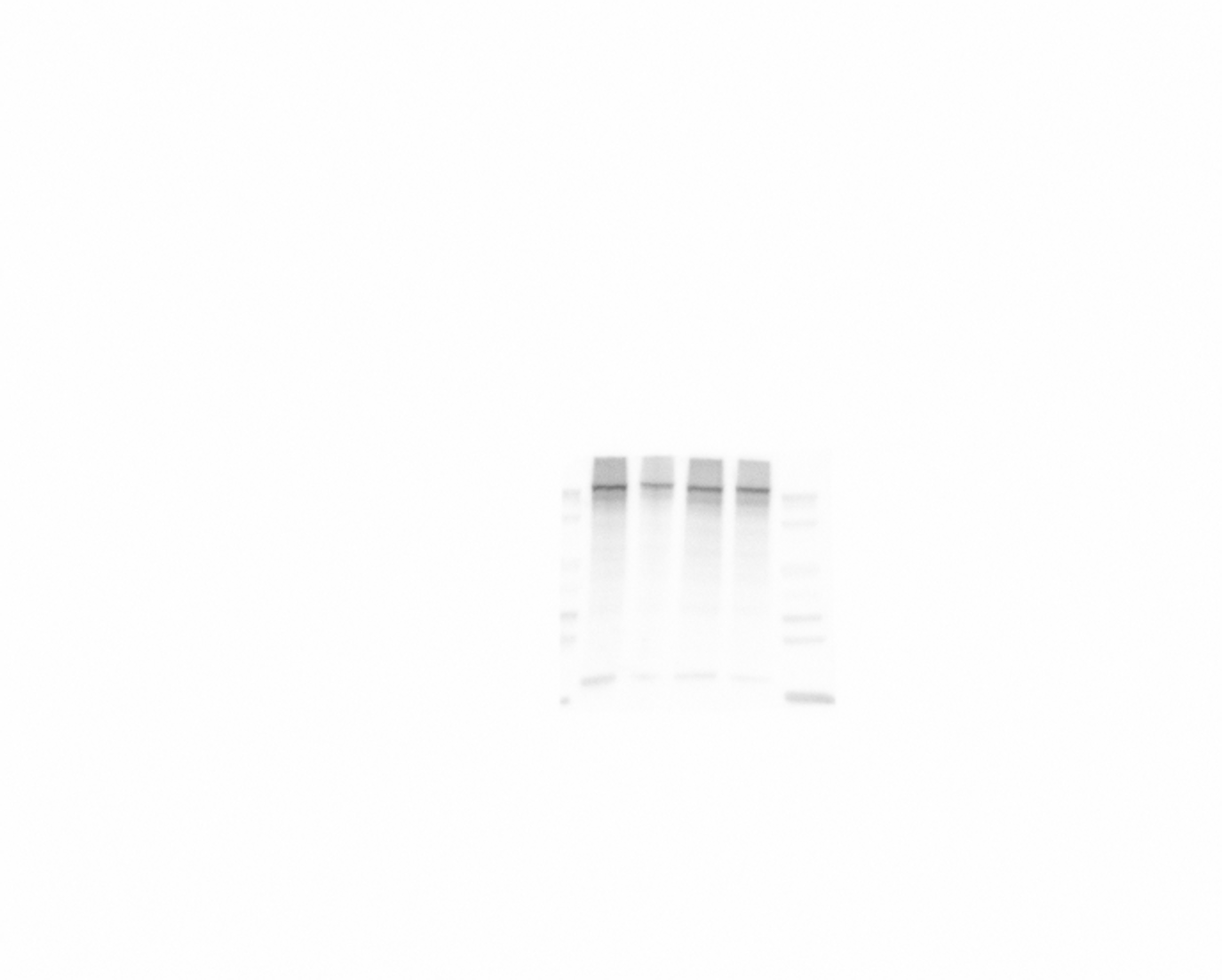

Supplement: Supplementary file 1 [file Data_Sheet_1.zip › the full uncropped Gels and Blots images/Group 2 n=3/ZO-1/1-2s.Tif]

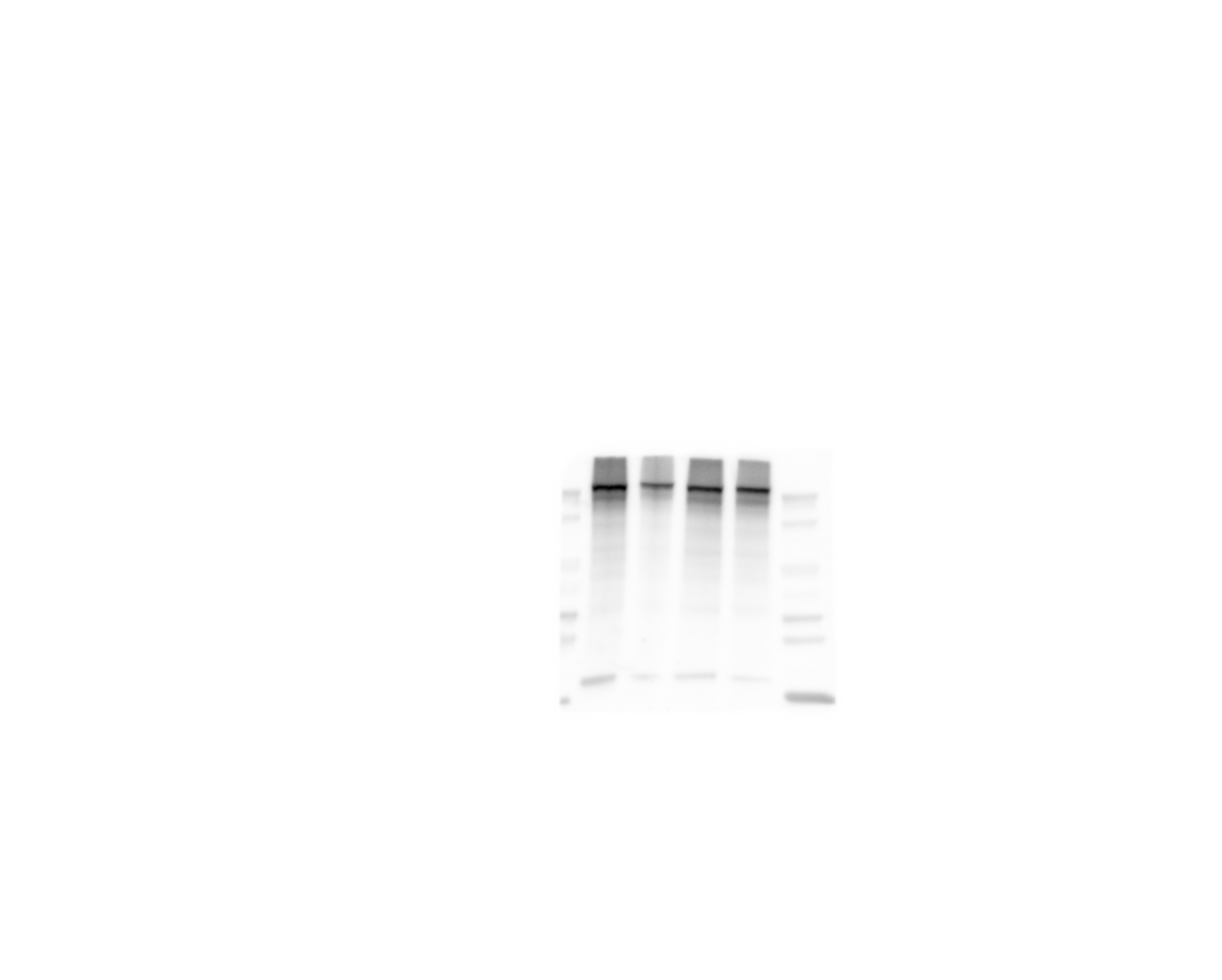

Supplement: Supplementary file 1 [file Data_Sheet_1.zip › the full uncropped Gels and Blots images/Group 2 n=3/ZO-1/1-3s.Tif]

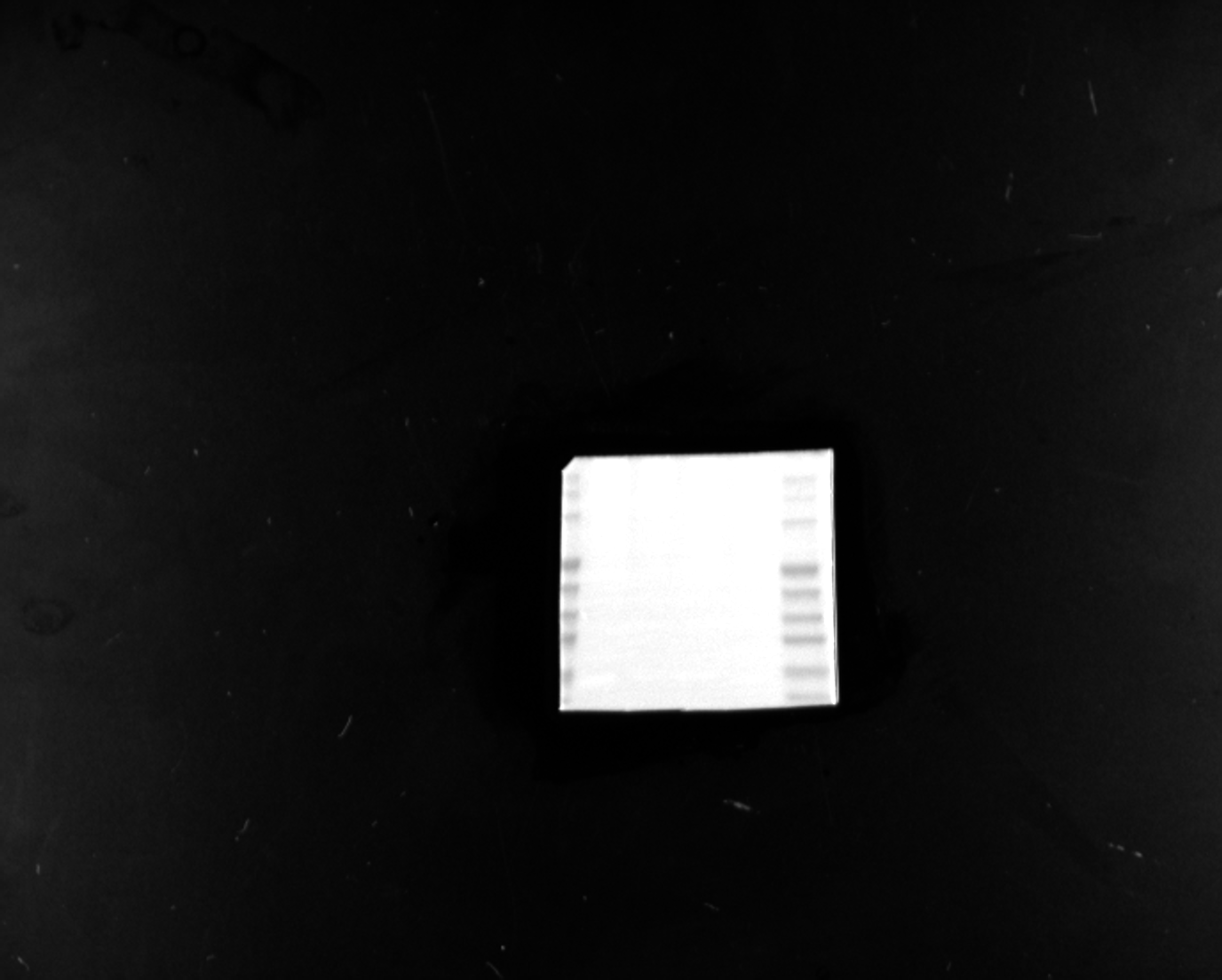

Supplement: Supplementary file 1 [file Data_Sheet_1.zip › the full uncropped Gels and Blots images/Group 2 n=3/ZO-1/1-t.Tif]

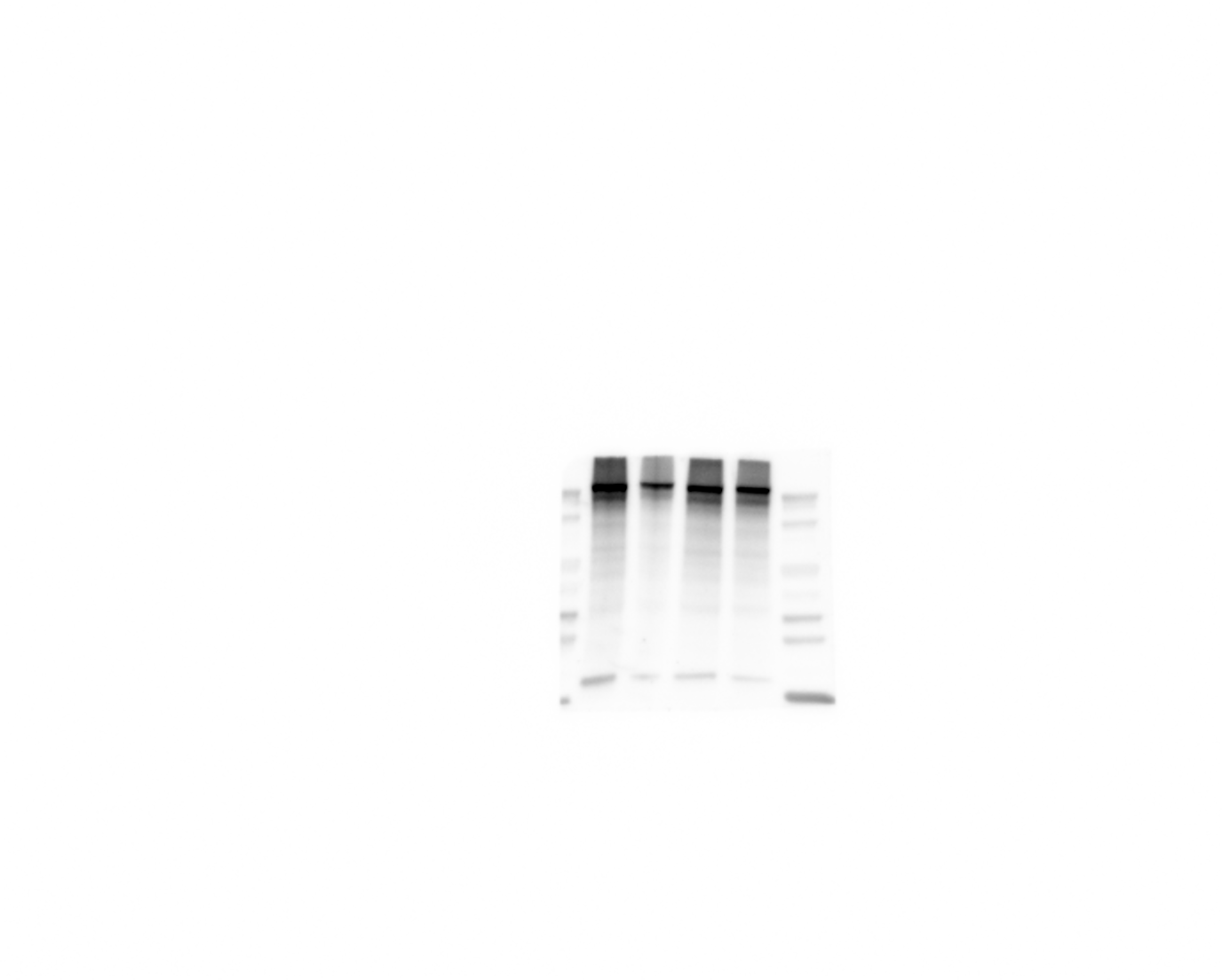

Supplement: Supplementary file 1 [file Data_Sheet_1.zip › the full uncropped Gels and Blots images/Group 2 n=3/ZO-1/1.Tif]

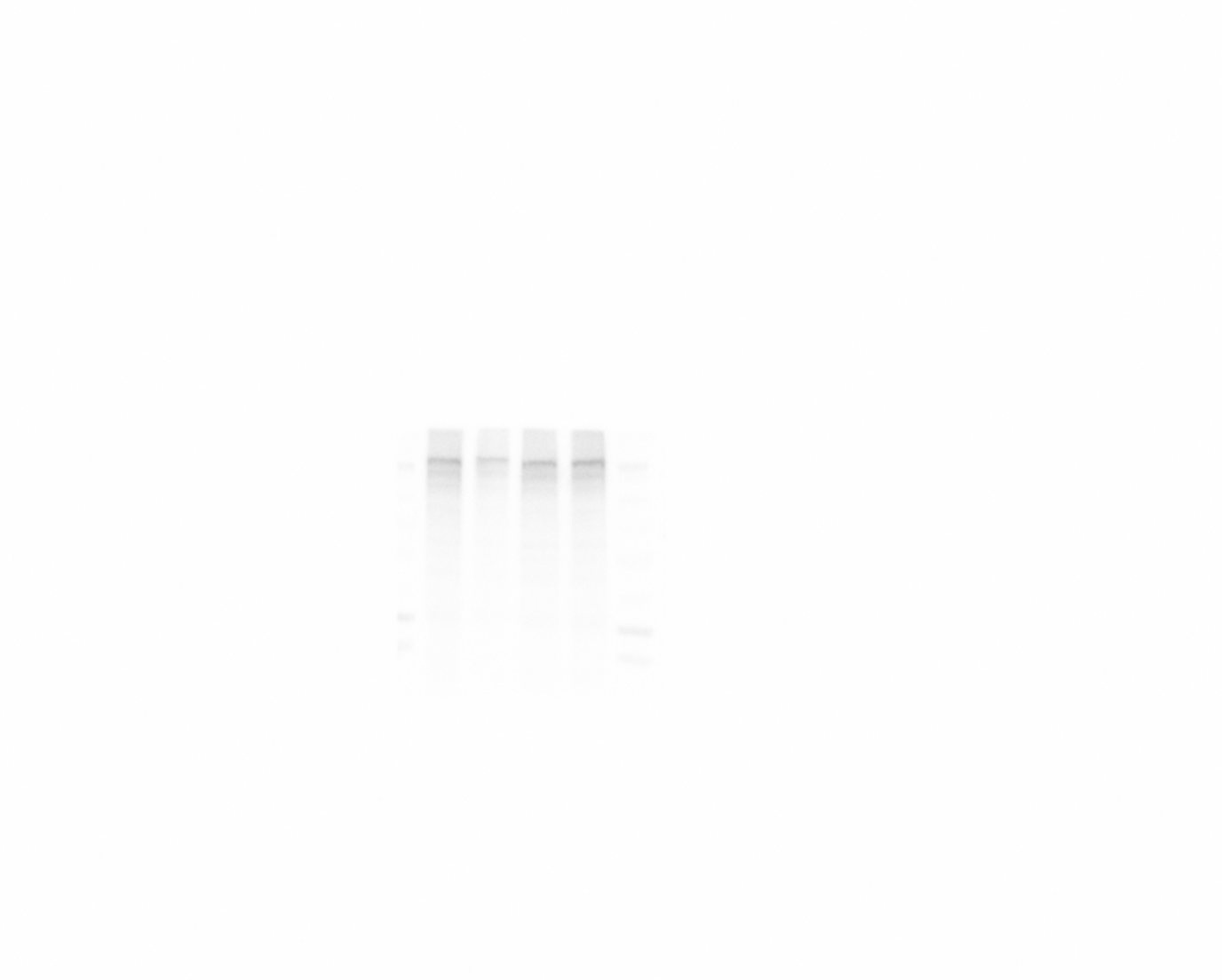

Supplement: Supplementary file 1 [file Data_Sheet_1.zip › the full uncropped Gels and Blots images/Group 2 n=3/ZO-1/2-2s.Tif]

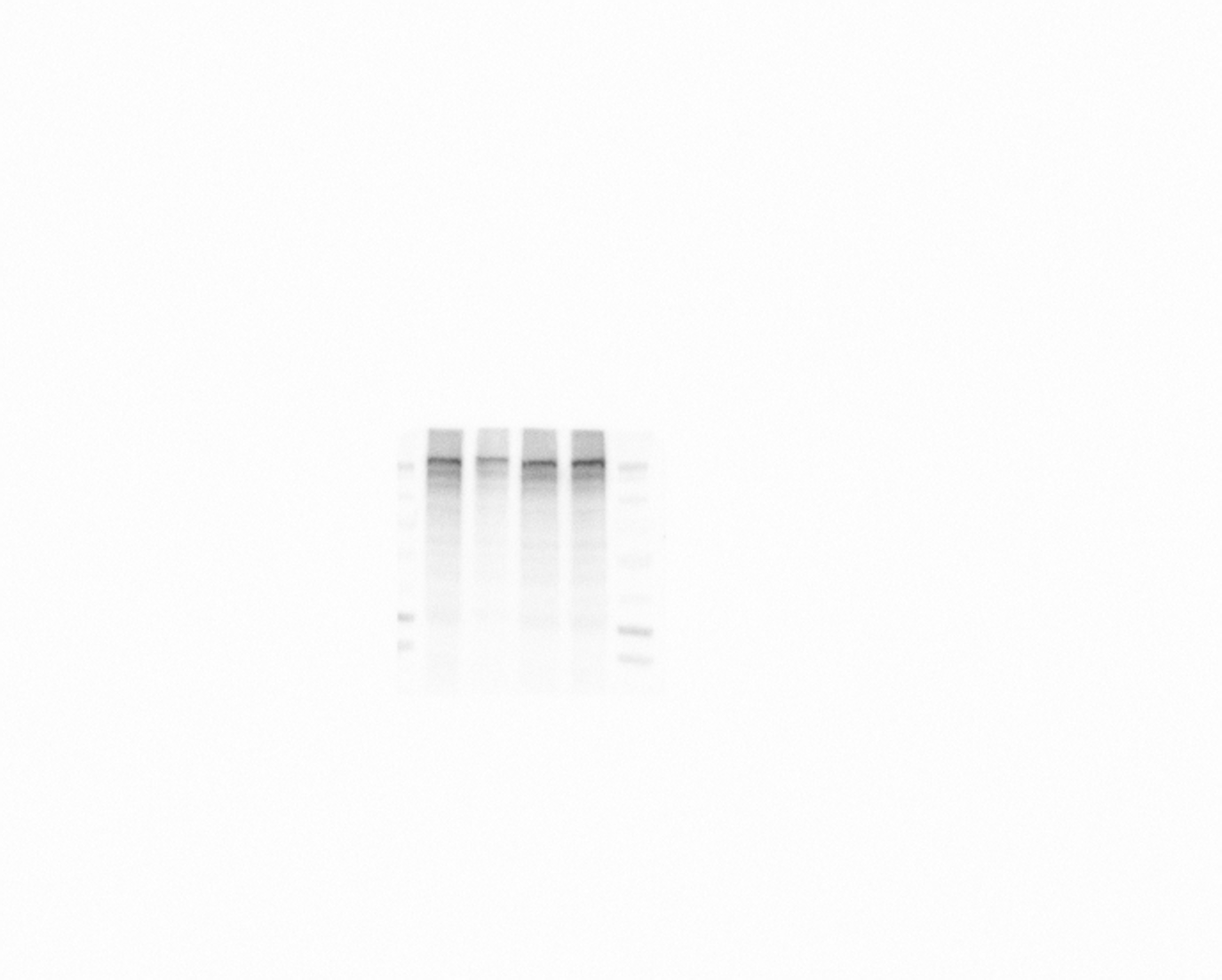

Supplement: Supplementary file 1 [file Data_Sheet_1.zip › the full uncropped Gels and Blots images/Group 2 n=3/ZO-1/2-3s.Tif]

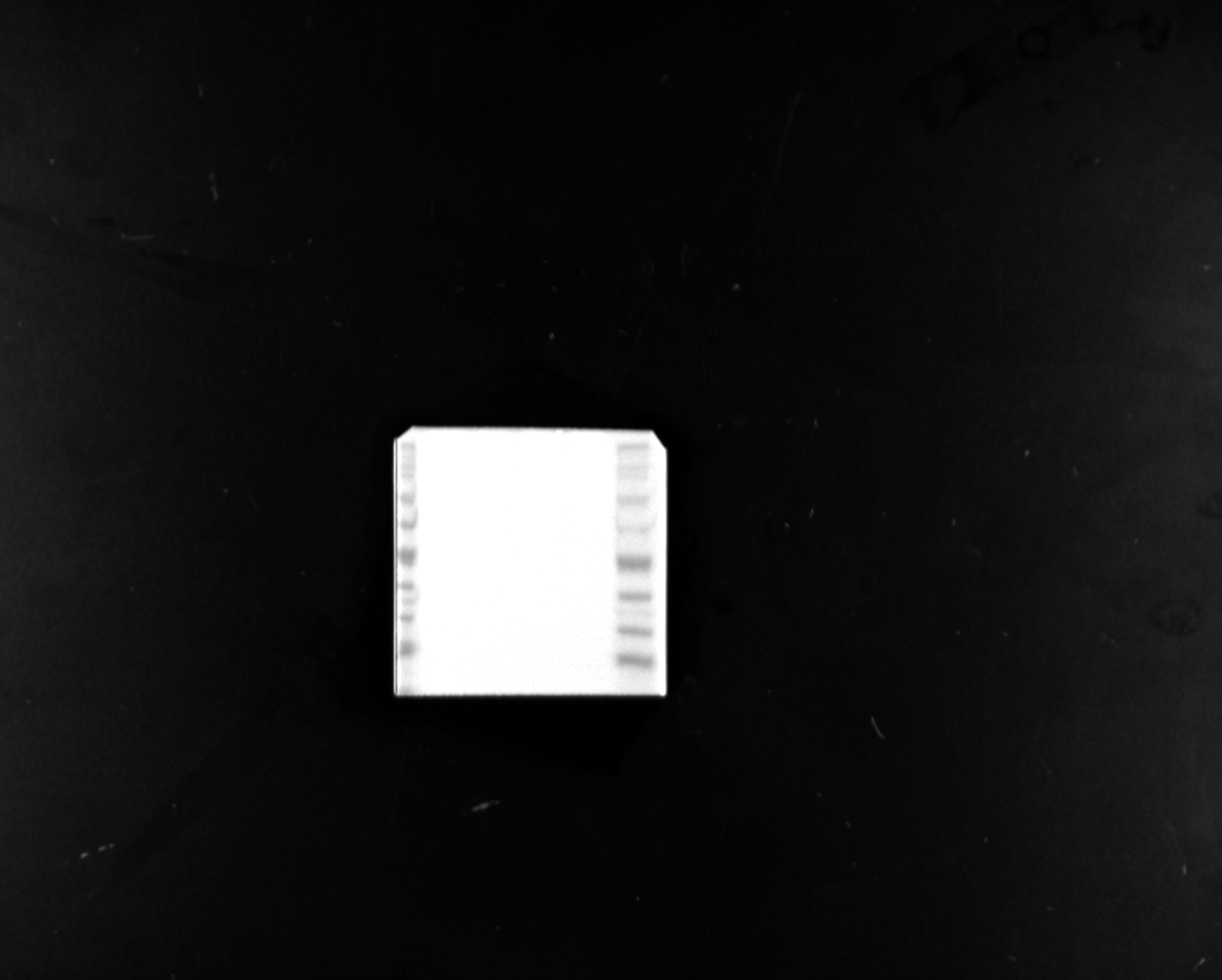

Supplement: Supplementary file 1 [file Data_Sheet_1.zip › the full uncropped Gels and Blots images/Group 2 n=3/ZO-1/2-t.Tif]

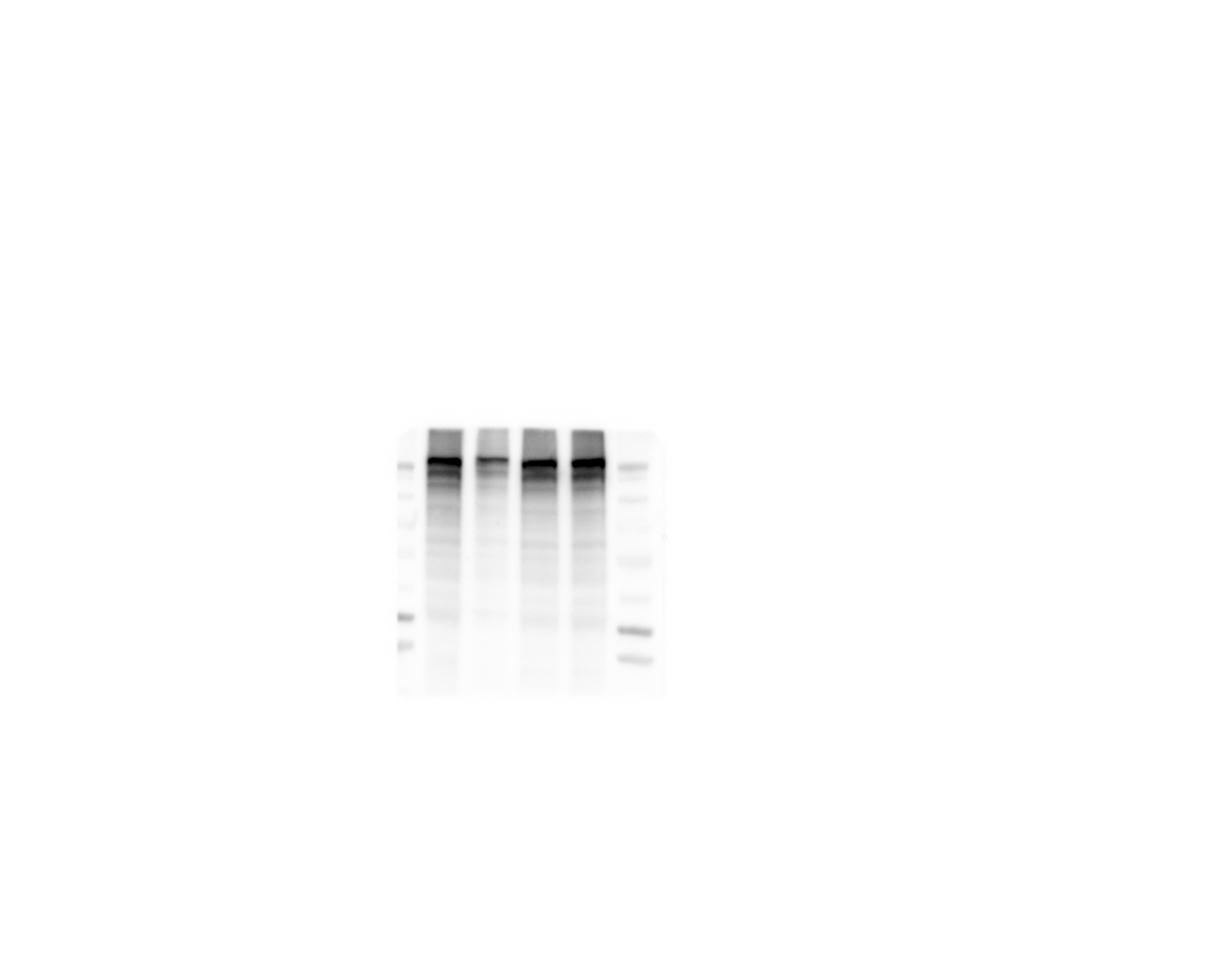

Supplement: Supplementary file 1 [file Data_Sheet_1.zip › the full uncropped Gels and Blots images/Group 2 n=3/ZO-1/2.Tif]

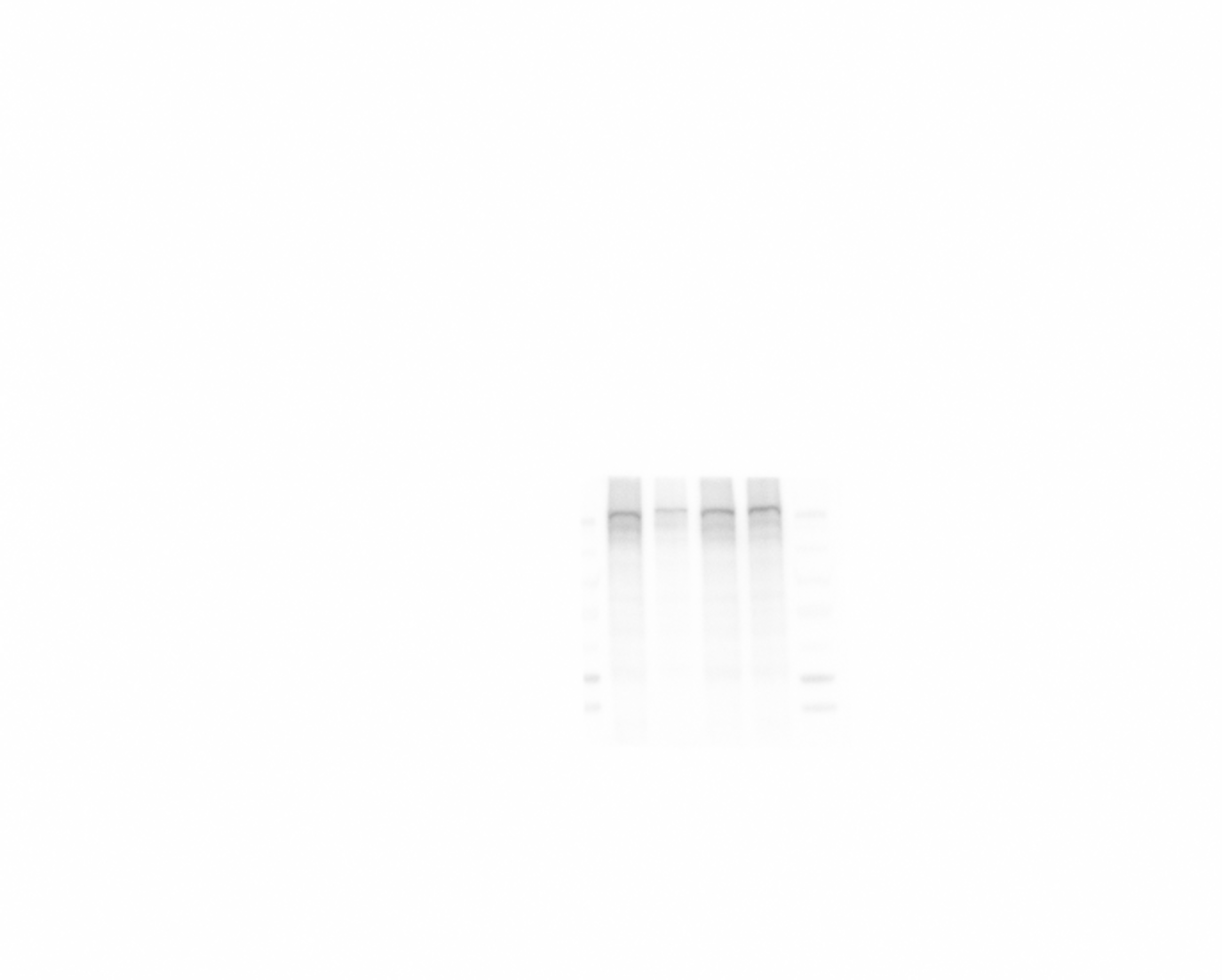

Supplement: Supplementary file 1 [file Data_Sheet_1.zip › the full uncropped Gels and Blots images/Group 2 n=3/ZO-1/3-2s.Tif]

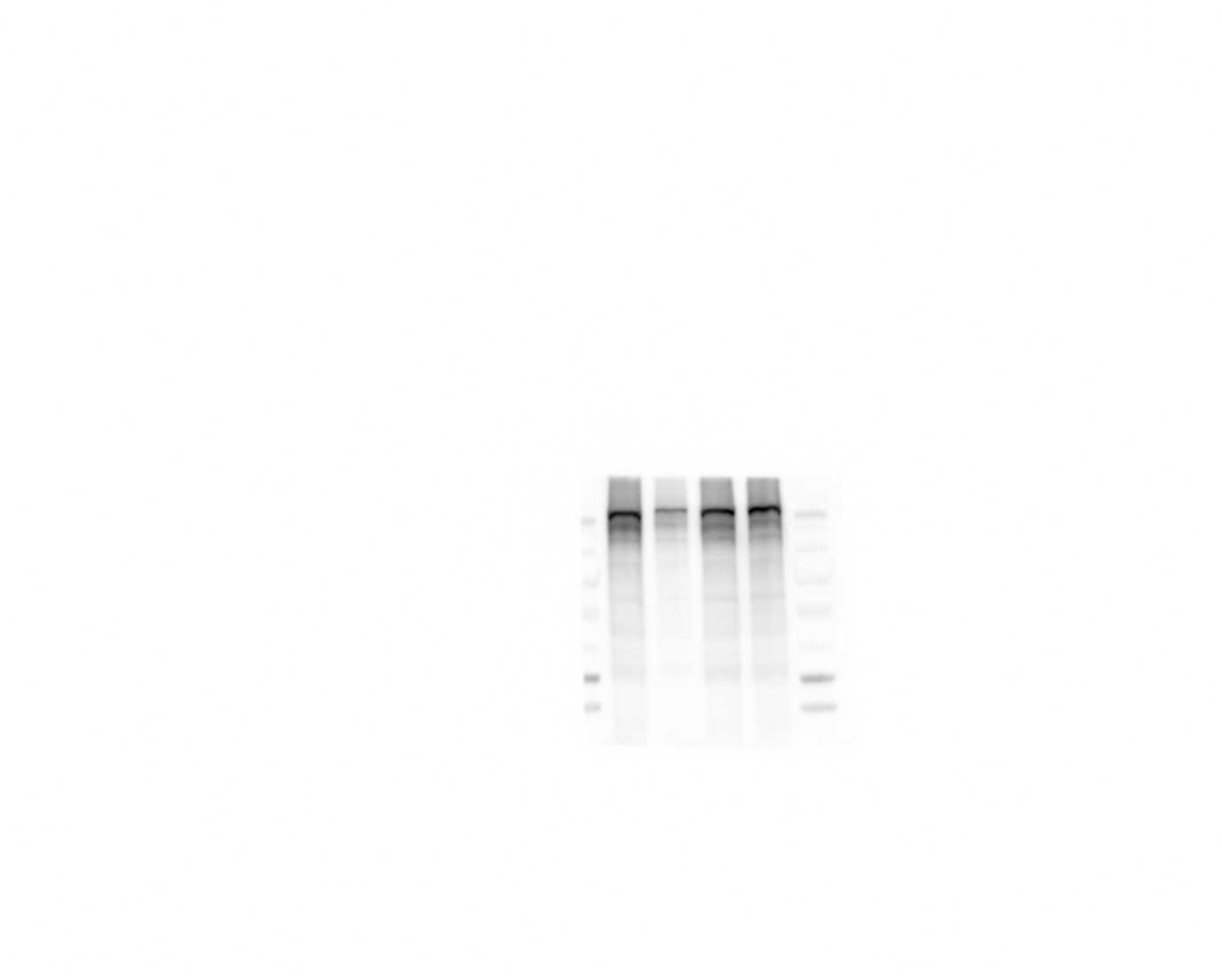

Supplement: Supplementary file 1 [file Data_Sheet_1.zip › the full uncropped Gels and Blots images/Group 2 n=3/ZO-1/3-3s.Tif]

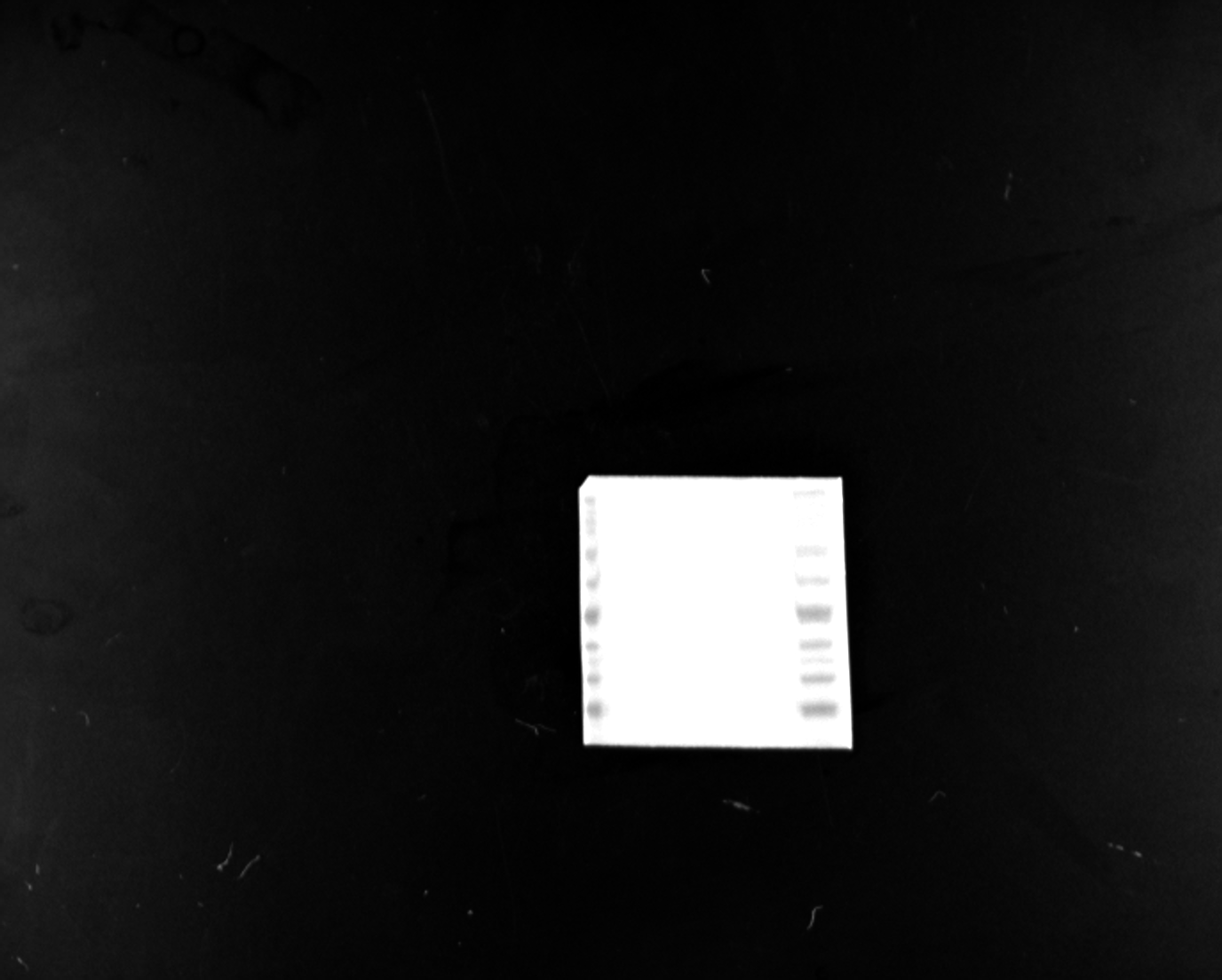

Supplement: Supplementary file 1 [file Data_Sheet_1.zip › the full uncropped Gels and Blots images/Group 2 n=3/ZO-1/3-t.Tif]

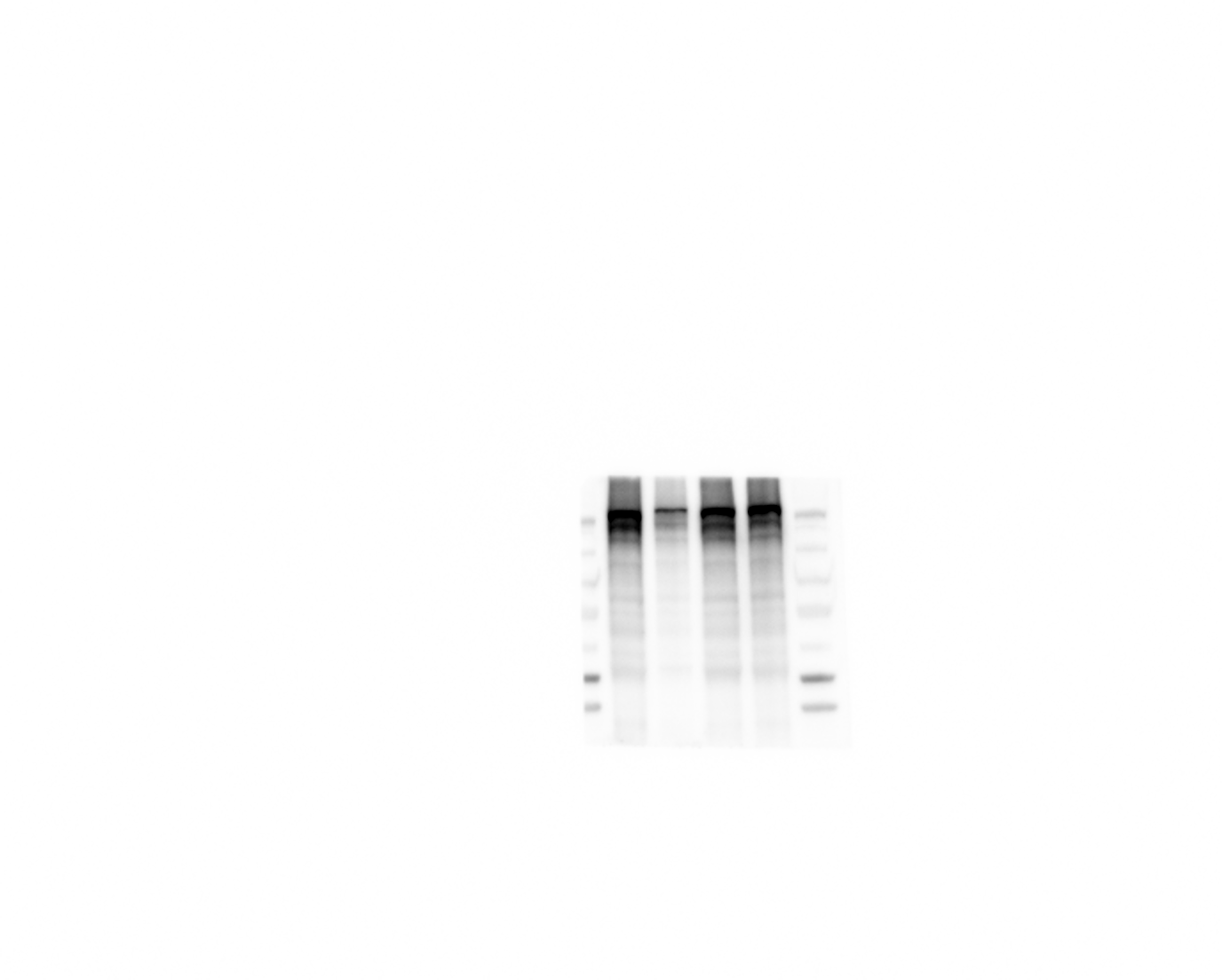

Supplement: Supplementary file 1 [file Data_Sheet_1.zip › the full uncropped Gels and Blots images/Group 2 n=3/ZO-1/3.Tif]

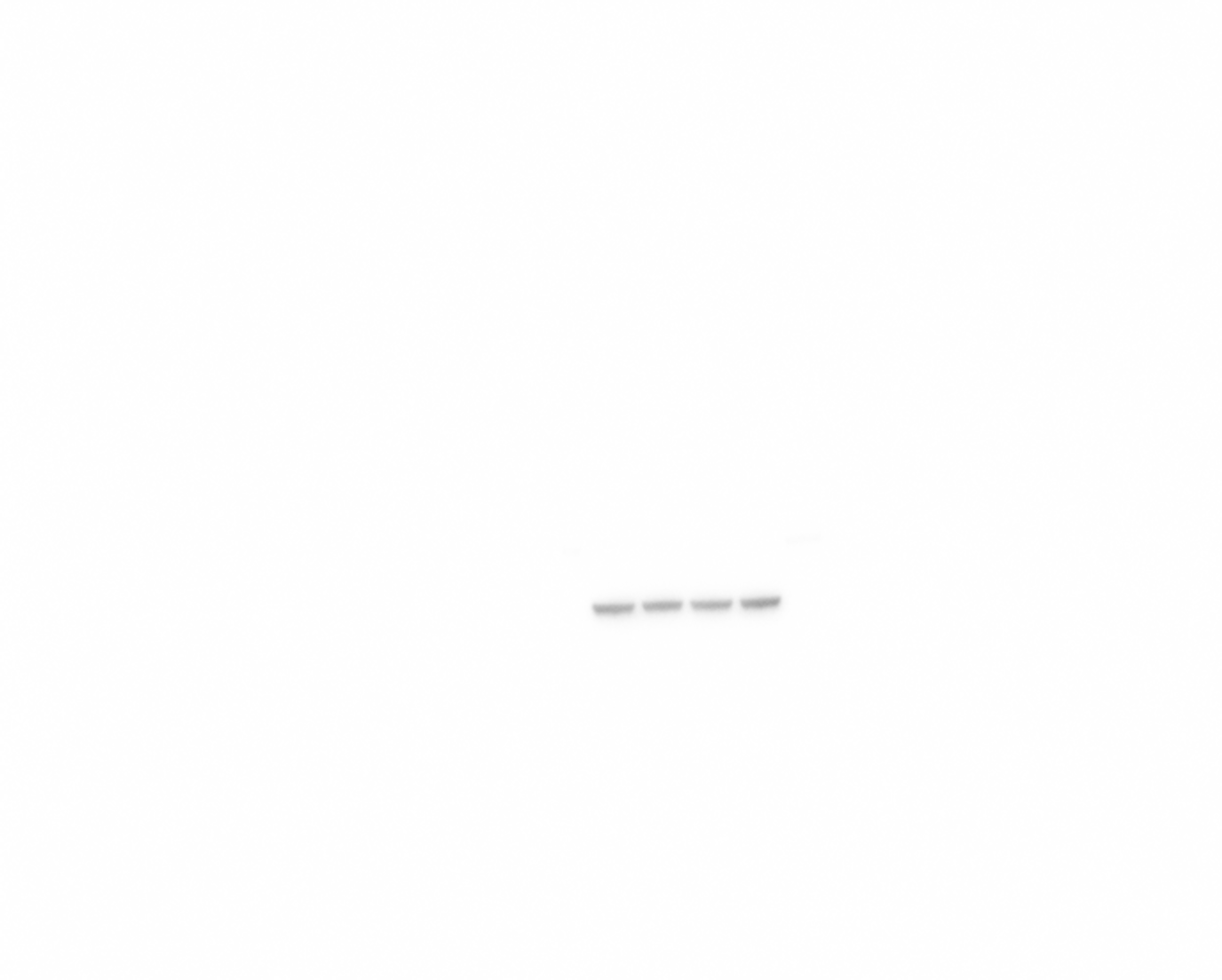

Supplement: Supplementary file 1 [file Data_Sheet_1.zip › the full uncropped Gels and Blots images/Group 2 n=3/β-actin/1-2s.Tif]

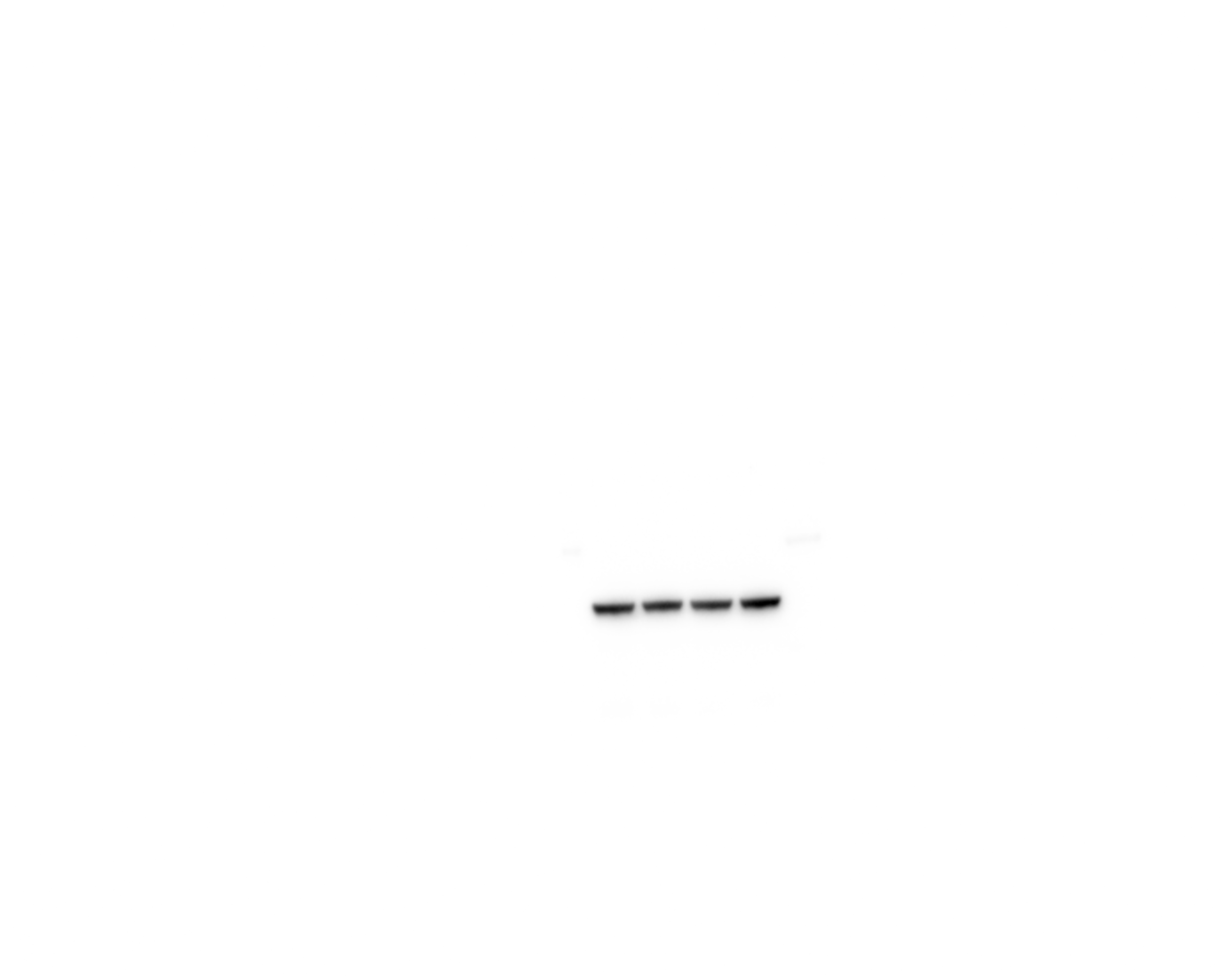

Supplement: Supplementary file 1 [file Data_Sheet_1.zip › the full uncropped Gels and Blots images/Group 2 n=3/β-actin/1-5s.Tif]

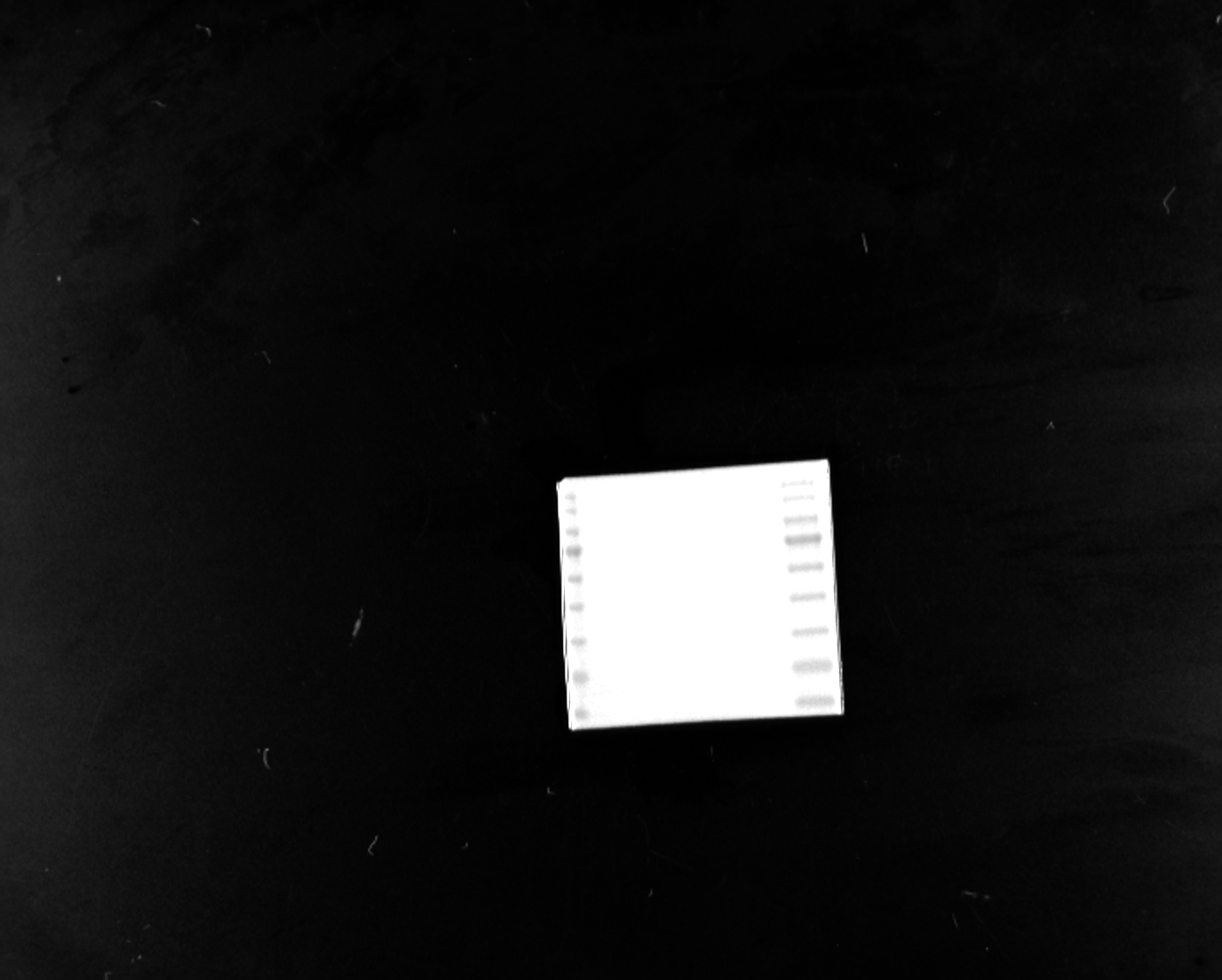

Supplement: Supplementary file 1 [file Data_Sheet_1.zip › the full uncropped Gels and Blots images/Group 2 n=3/β-actin/1-t.Tif]

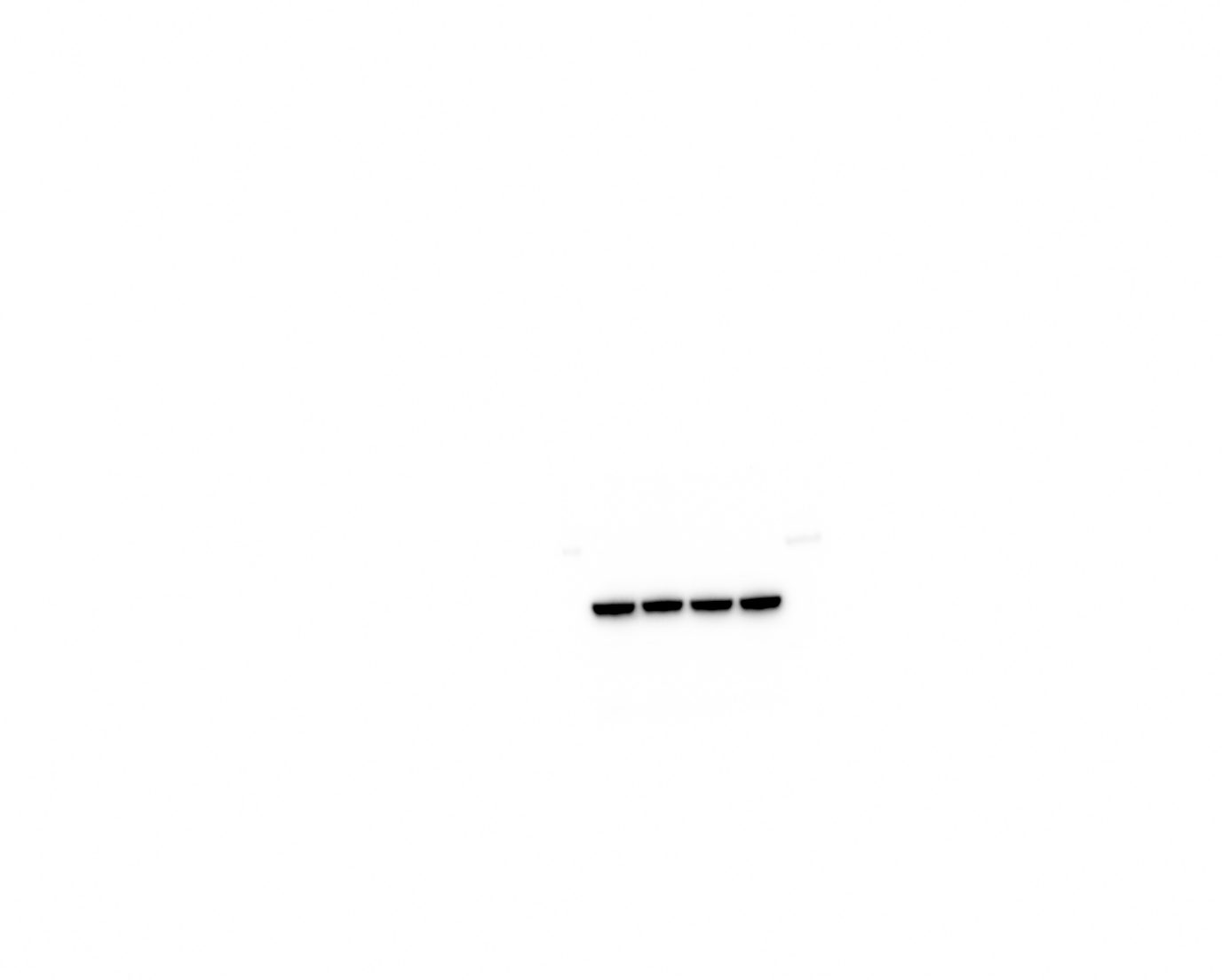

Supplement: Supplementary file 1 [file Data_Sheet_1.zip › the full uncropped Gels and Blots images/Group 2 n=3/β-actin/1.Tif]

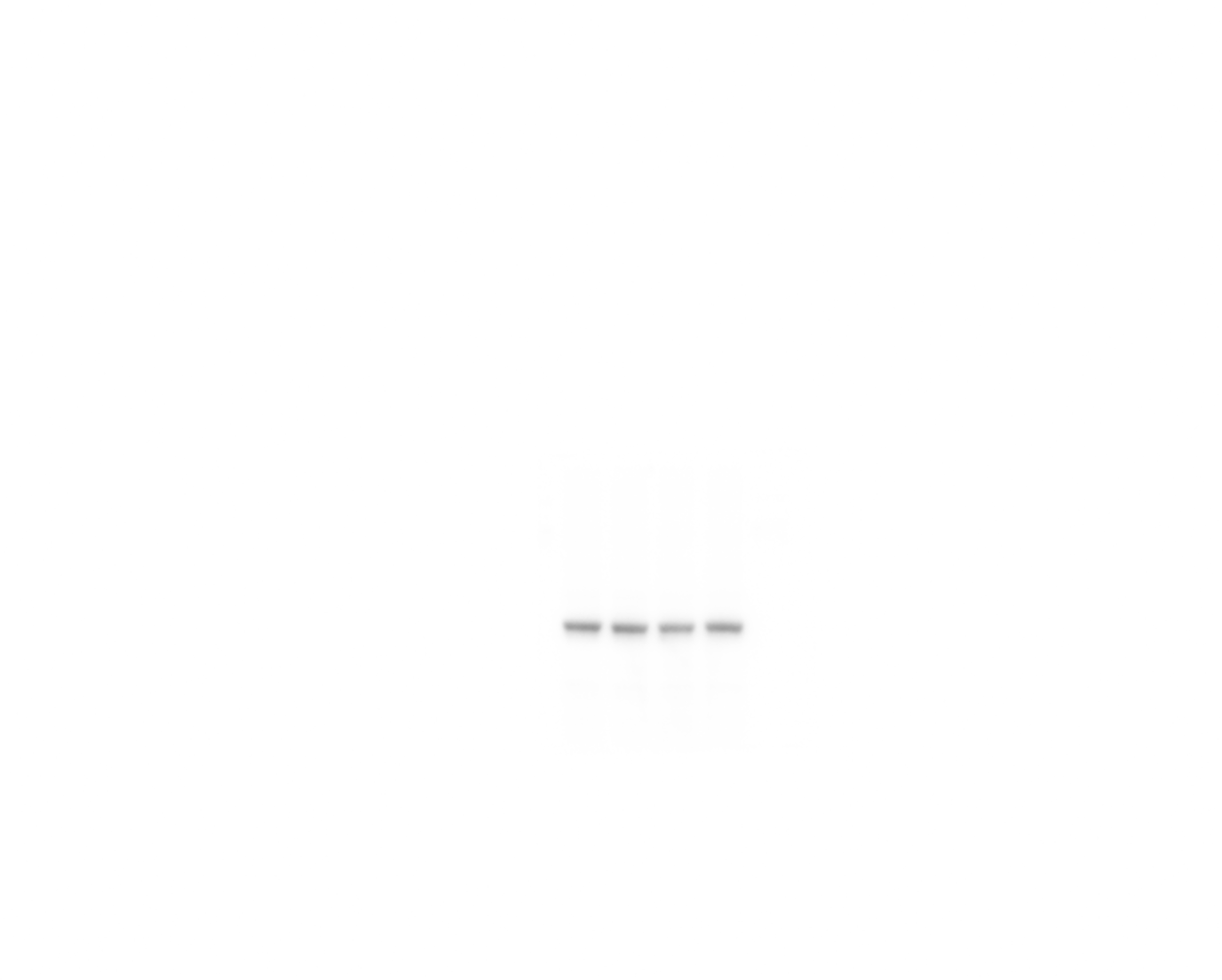

Supplement: Supplementary file 1 [file Data_Sheet_1.zip › the full uncropped Gels and Blots images/Group 2 n=3/β-actin/2-2s.Tif]

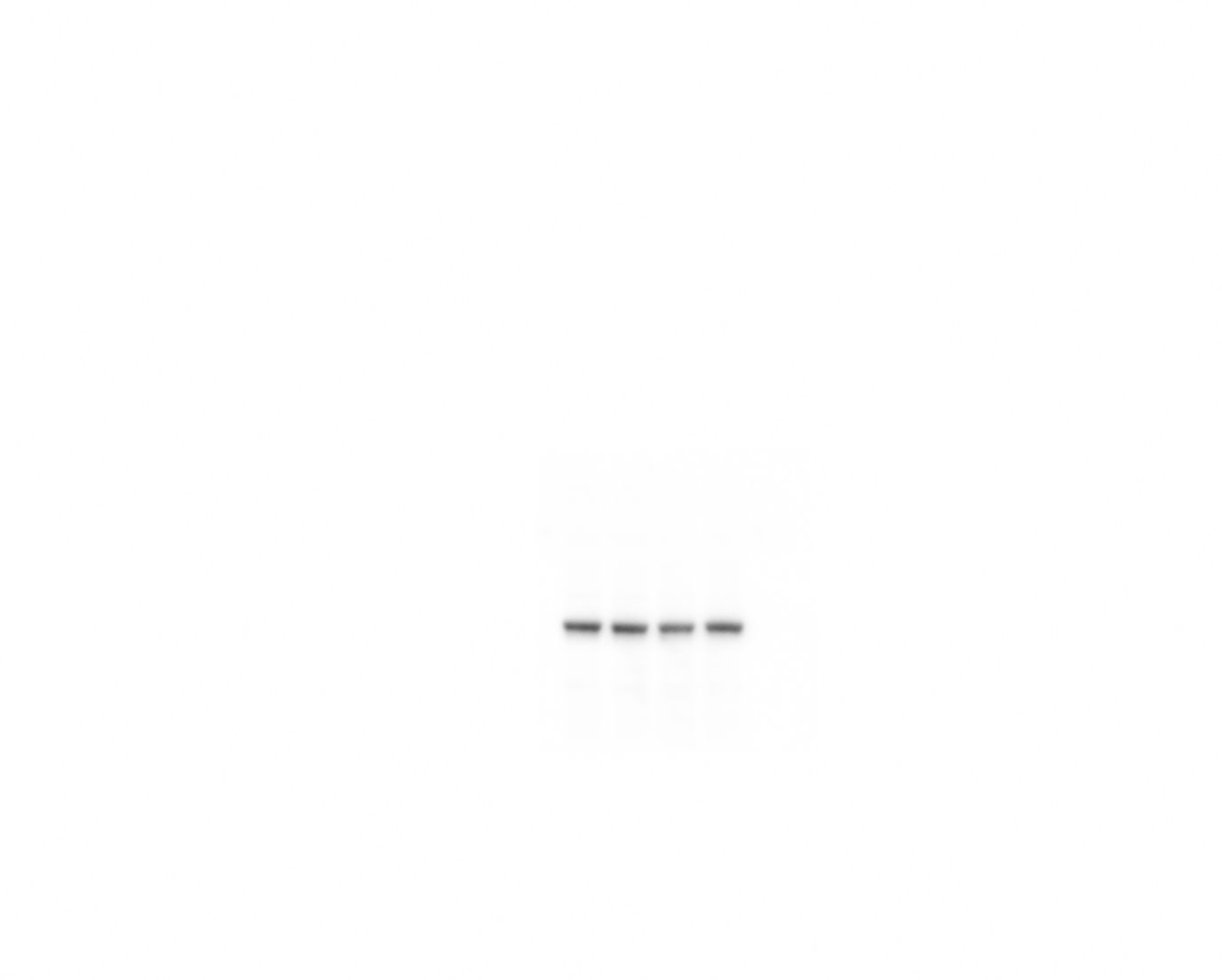

Supplement: Supplementary file 1 [file Data_Sheet_1.zip › the full uncropped Gels and Blots images/Group 2 n=3/β-actin/2-3s.Tif]

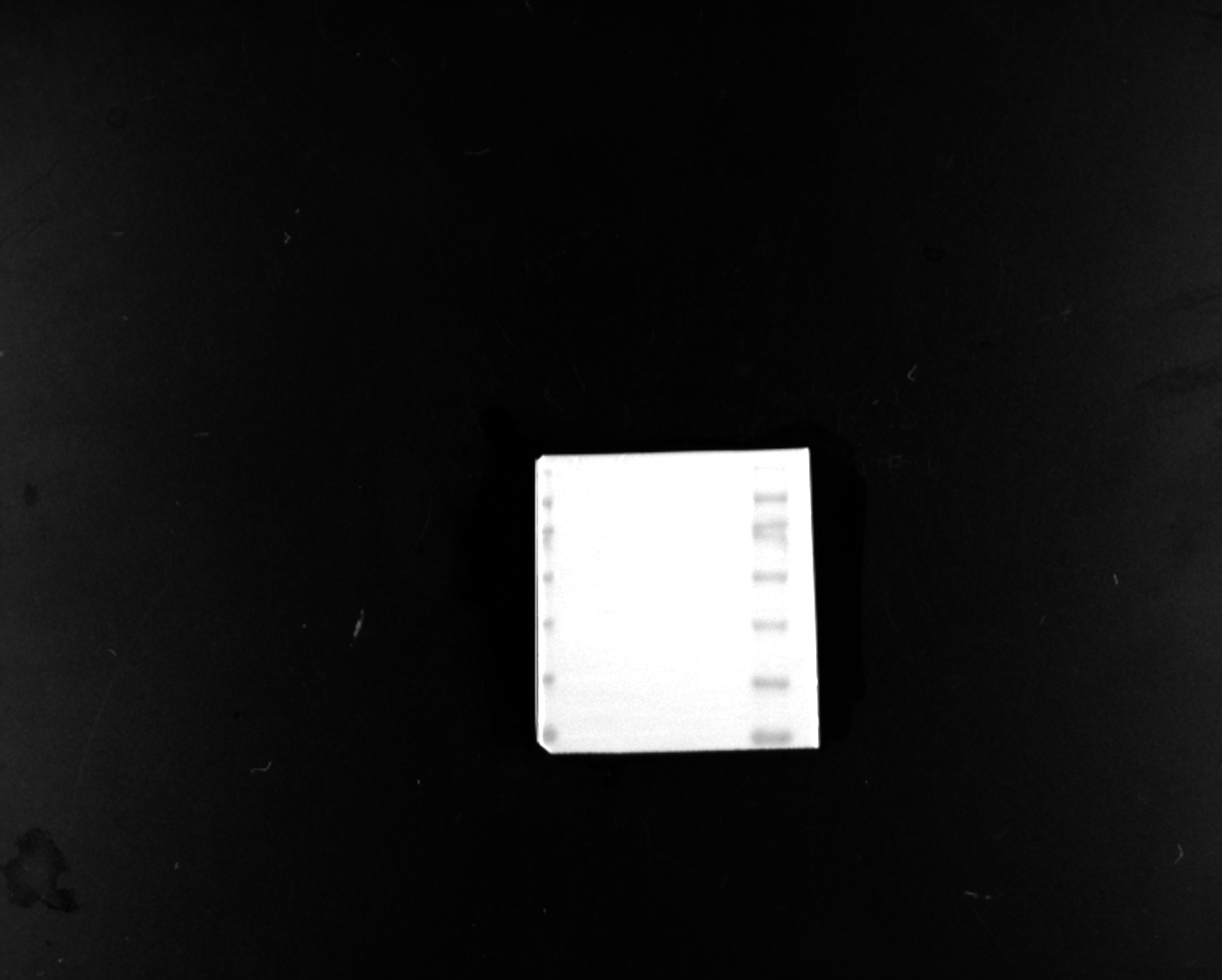

Supplement: Supplementary file 1 [file Data_Sheet_1.zip › the full uncropped Gels and Blots images/Group 2 n=3/β-actin/2-t.Tif]

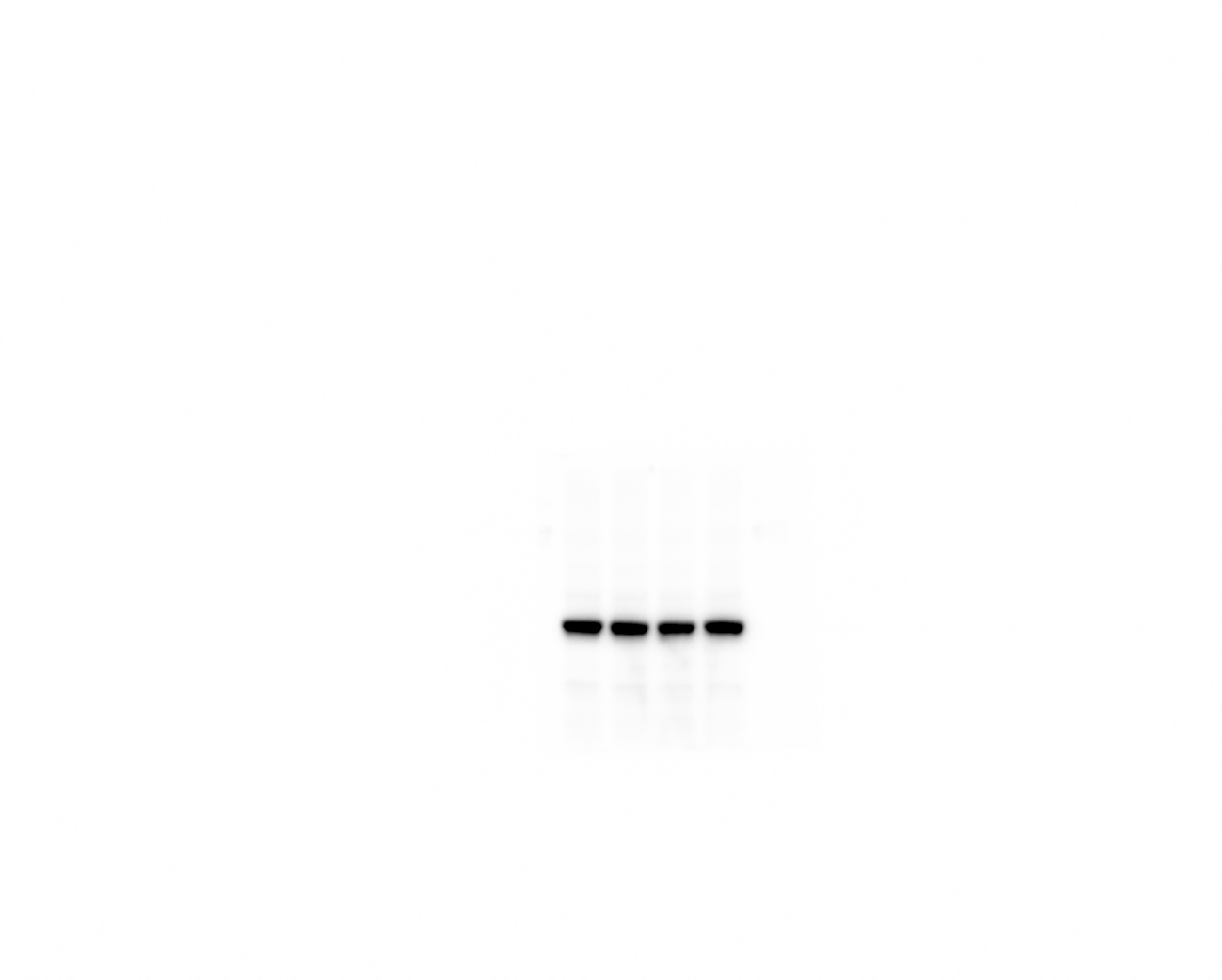

Supplement: Supplementary file 1 [file Data_Sheet_1.zip › the full uncropped Gels and Blots images/Group 2 n=3/β-actin/2.Tif]

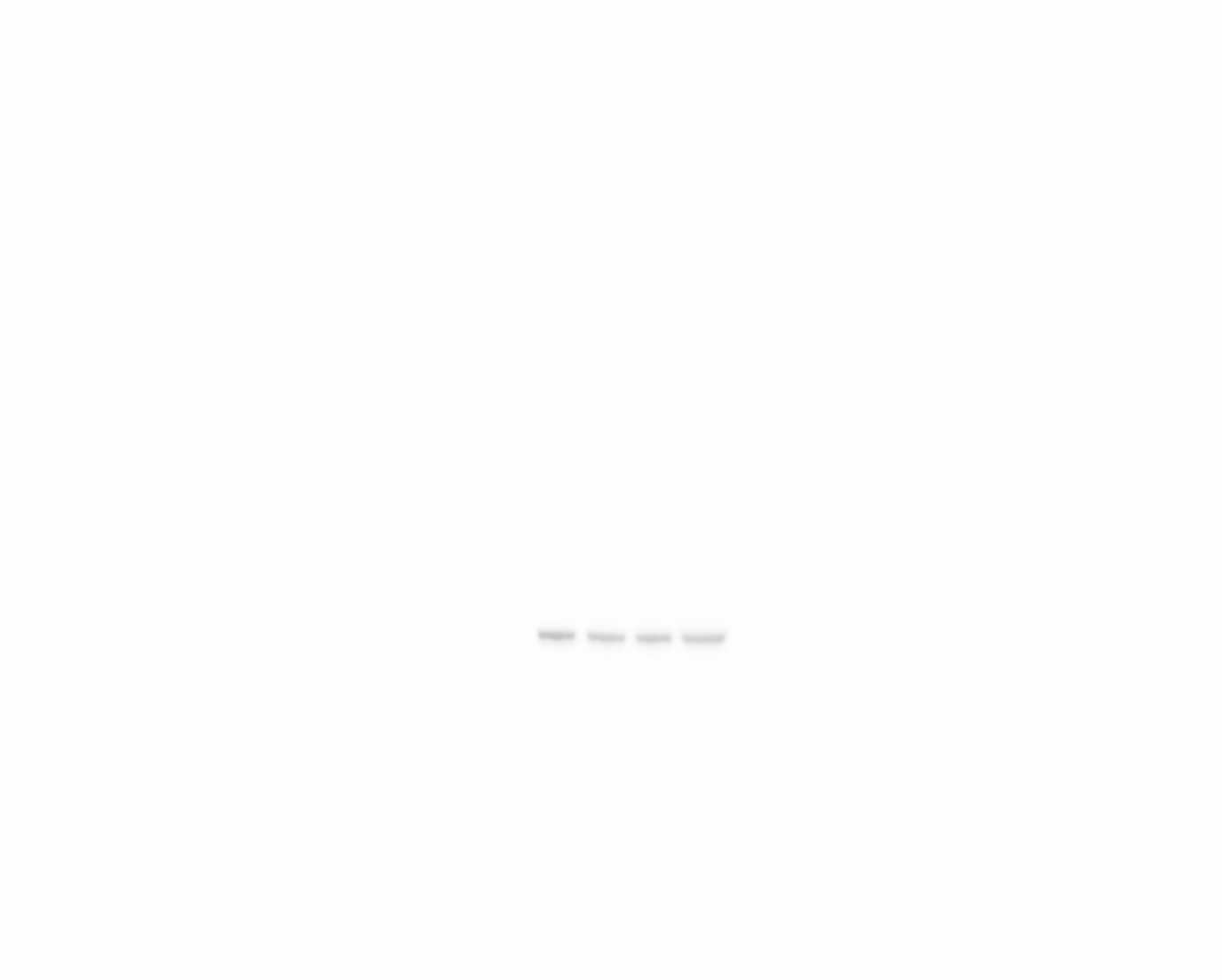

Supplement: Supplementary file 1 [file Data_Sheet_1.zip › the full uncropped Gels and Blots images/Group 2 n=3/β-actin/3-2s.Tif]

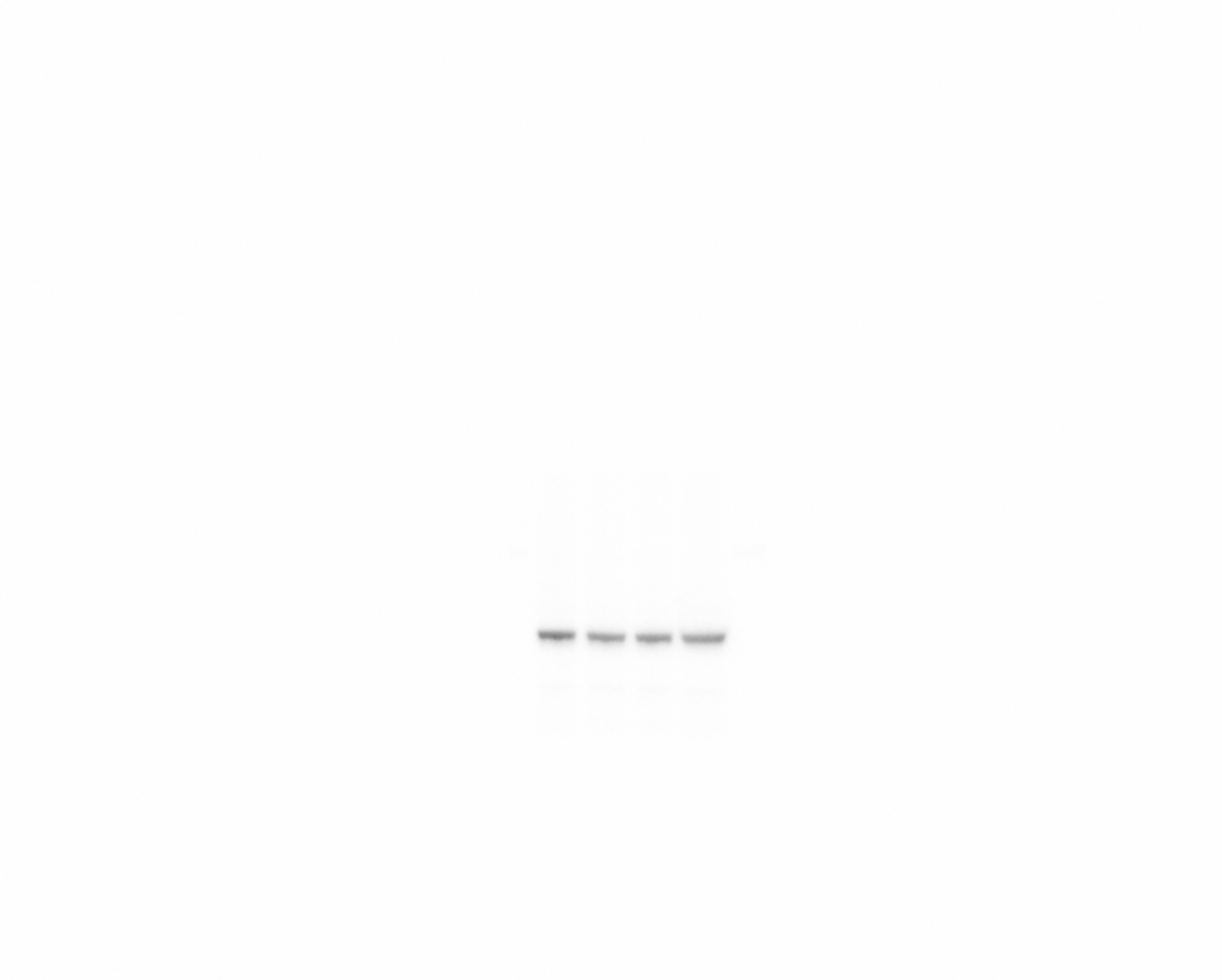

Supplement: Supplementary file 1 [file Data_Sheet_1.zip › the full uncropped Gels and Blots images/Group 2 n=3/β-actin/3-3s.Tif]

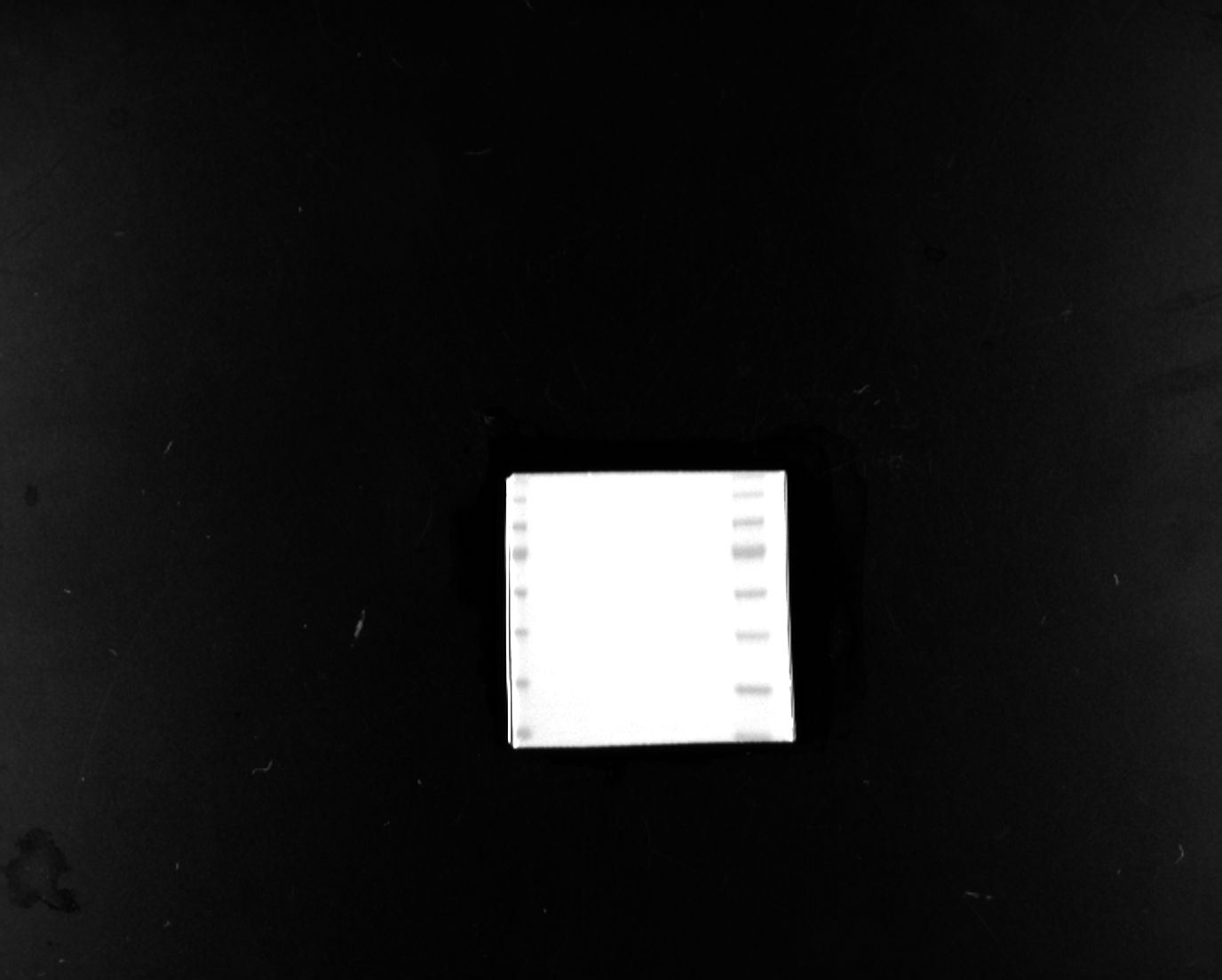

Supplement: Supplementary file 1 [file Data_Sheet_1.zip › the full uncropped Gels and Blots images/Group 2 n=3/β-actin/3-t.Tif]

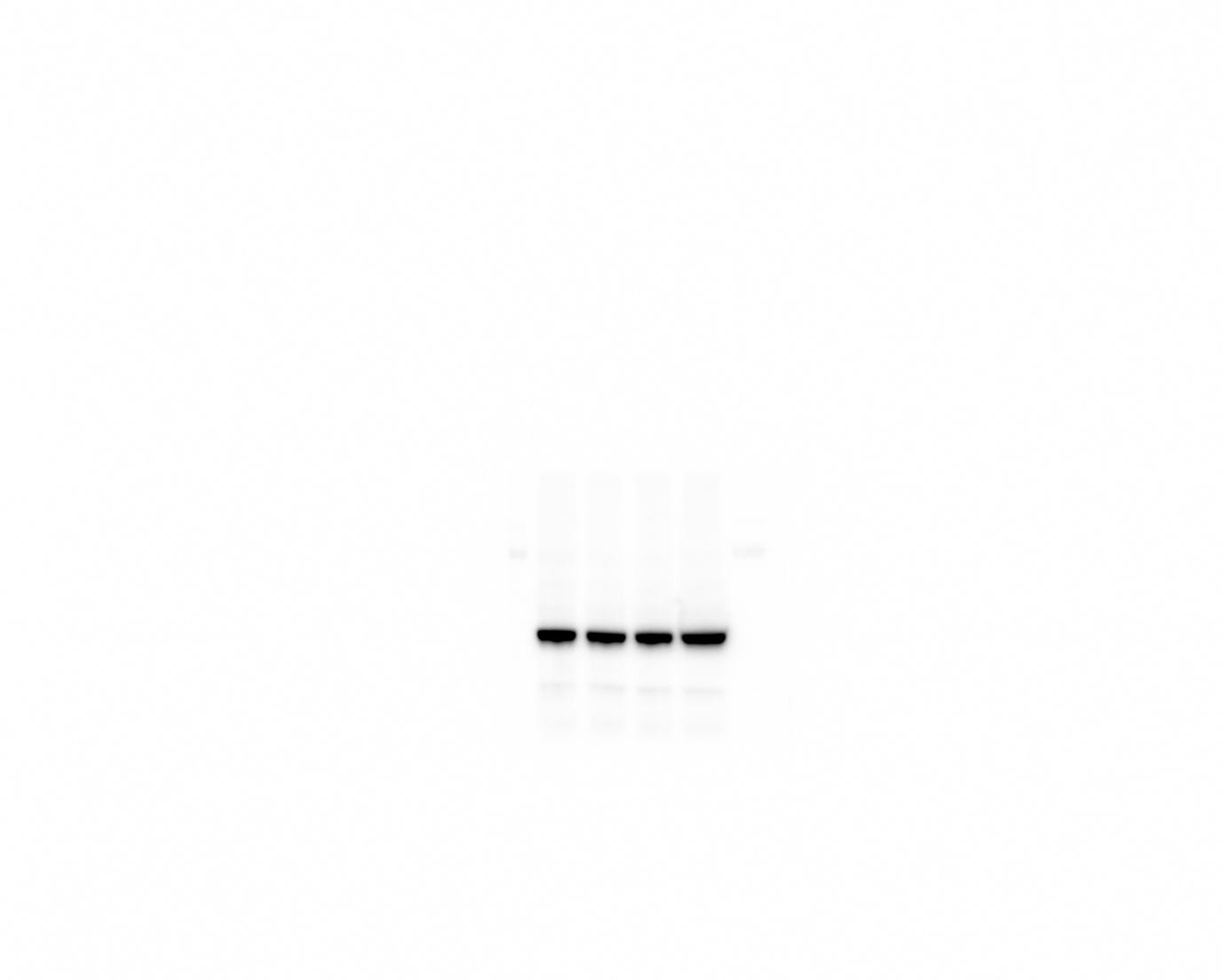

Supplement: Supplementary file 1 [file Data_Sheet_1.zip › the full uncropped Gels and Blots images/Group 2 n=3/β-actin/3.Tif]
